# Sirolimus reduces T cell cycling, immune checkpoint marker expression, and HIV-1 DNA in people with HIV

**DOI:** 10.1016/j.xcrm.2024.101745

**Published:** 2024-09-24

**Authors:** Timothy J. Henrich, Ronald J. Bosch, Catherine Godfrey, Hanna Mar, Apsara Nair, Michael Keefer, Carl Fichtenbaum, Daniela Moisi, Brian Clagett, Amanda M. Buck, Amelia N. Deitchman, Francesca Aweeka, Jonathan Z. Li, Daniel R. Kuritzkes, Michael M. Lederman, Priscilla Y. Hsue, Steven G. Deeks, the ACTG A5337 Team, Danielle Campbell, Corey Cutler, Michael Dorosh, Belinda Ha, Elizabeth Hawkins, Christopher Hensel, Nayri Khairalla, Kevin Knowles, Sulggi A. Lee, Susan Pedersen, Justin Ritz, Dylan Ryder, Rafick Sekaly, David L. Shugarts, Becky Straub, Andrew Zolopa

**Affiliations:** 1 Department of Medicine, University of California San Francisco, San Francisco, CA 94110, USA; 2 Center for Biostatistics in AIDS Research, Harvard T.H. Chan School of Public Health, Boston, MA 02115, USA; 3 Division of AIDS, National Institute of Allergy and Infectious Diseases, Bethesda, MD 20892, USA; 4 Frontier Science and Technology Research Foundation, Amherst, NY 14226, USA; 5 Department of Medicine, University of Rochester School of Medicine, Rochester, NY 14642, USA; 6 Department of Medicine, University of Cincinnati College of Medicine, Cincinnati, OH 45267, USA; 7 Department of Medicine, Case Western Reserve University School of Medicine, Cleveland, OH 44106, USA; 8 San Francisco State University, San Francisco, CA 94132, USA; 9 Department of Clinical Pharmacology, University of California San Francisco, San Francisco, CA 94110, USA; 10 Division of Infectious Diseases, Brigham and Women’s Hospital, Harvard Medical School, Boston, MA 02115, USA

**Keywords:** HIV-1, sirolimus, mTOR inhibition, HIV persistence, HIV reservoirs, immunotherapy, HIV curative strategies, HIV immunology, intact proviral HIV-1 DNA, antiproliferative medications

## Abstract

Key HIV cure strategies involve reversing immune dysfunction and limiting the proliferation of infected T cells. We evaluate the safety of sirolimus, a mammalian target of rapamycin (mTOR) inhibitor, in people with HIV (PWH) and study the impact of sirolimus on HIV-1 reservoir size and HIV-1-specific immunity in a single-arm study of 20 weeks of treatment in PWH on antiretroviral therapy (ART). Sirolimus treatment does not impact HIV-1-specific CD8 T cell responses but leads to a significant decrease in CD4^+^ T cell-associated HIV-1 DNA levels at 20 weeks of therapy in the primary efficacy population (*n* = 16; 31% decline, *p* = 0.008). This decline persists for at least 12 weeks following cessation of the study drug. Sirolimus treatment also leads to a significant reduction in CD4^+^ T cell cycling and PD-1 expression on CD8^+^ lymphocytes. These data suggest that homeostatic proliferation of infected cells, an important mechanism for HIV persistence, is an intriguing therapeutic target.

## Highlights

• Sirolimus treatment decreases cell-associated HIV-1 levels in people with HIV on ART
• The reduction in HIV DNA persists for at least 12 weeks following sirolimus treatment
• Sirolimus reduces CD4^+^ T cell cycling and CD8^+^ T cell PD-1 expression
• Sirolimus does not impact HIV-1-specific CD8 T cell responses or HIV-1 RNA levels

## Graphical abstract

**Figure:**
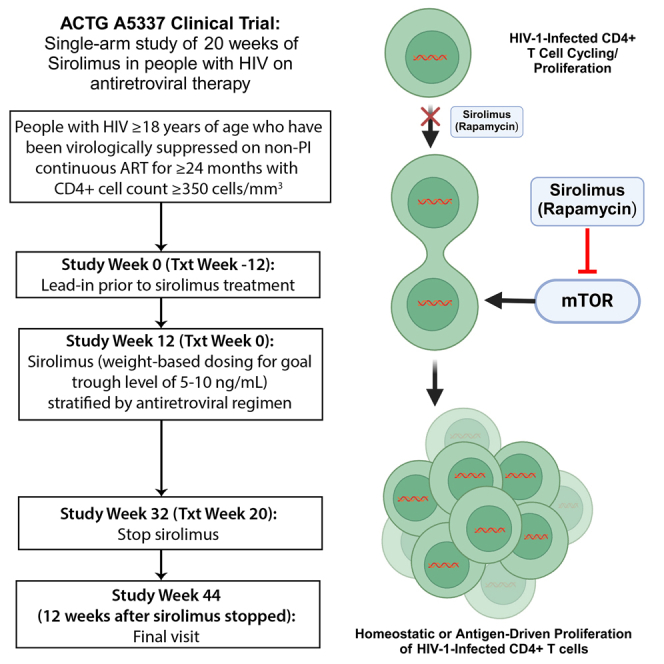

## Teaser

Henrich et al. show that sirolimus (rapamycin) reduces HIV-1 proviral DNA, perhaps through decreased cell cycling that limits the homeostatic proliferation of infected CD4^+^ T cells. Conceptually, these data suggest that homeostatic proliferation of infected cells, an important mechanism for HIV persistence, might be targeted therapeutically.

## Introduction

Despite sustained inhibition of virus replication by antiretroviral therapy (ART), HIV persists indefinitely. Chronic viral-associated inflammation and immune dysfunction also persist in most people with HIV (PWH). Substantial evidence supports the concept that the chronic inflammatory environment associated with treated HIV disease results in a dysfunctional virus-specific immune response and inability of the host immune response to clear the persistent viral reservoir.^1^^,^^2^^,^^3^ Furthermore, the proliferation of infected CD4^+^ T cells leads to the indefinite persistence of HIV reservoirs that escape immune targeting and clearance.^4^ To date, few prospective immunotherapy trials, including those that target cell proliferation and immune modulation, have demonstrated significant decreases in HIV-1 DNA burden *in vivo*, particularly in those on long-term, stable ART.

The mammalian target of rapamycin (mTOR) is a regulatory kinase that controls cell cycle progression.^5^^,^^6^^,^^7^^,^^8^^,^^9^^,^^10^ Despite its immunosuppressive consequences, pharmacologic mTOR inhibition leads to changes in several immune regulatory pathways that may enhance antiviral activity and limit CD4^+^ T cell homeostatic proliferation and cell cycling.^11^^,^^12^^,^^13^^,^^14^^,^^15^^,^^16^^,^^17^^,^^18^^,^^19^^,^^20^^,^^21^^,^^22^^,^^23^^,^^24^^,^^25^^,^^26^^,^^27^ As homeostatic proliferation of infected CD4^+^ T cells is a key mechanism for the maintenance of the latent HIV reservoir,^28^^,^^29^^,^^30^^,^^31^^,^^32^^,^^33^^,^^34^^,^^35^ targeting the heightened and dysregulated CD4^+^ T cell cycling is a plausible strategy to limit reservoir persistence.^4^ Sirolimus (rapamycin) is a selective TORC1 inhibitor with suppressive effects on cell cycle progression that predominately targets lymphocytes activated by cytokines rather than by antigen-T cell receptor engagement.^36^^,^^37^ In treated and untreated HIV infection, increased CD4^+^ and CD8^+^ T cell cycling and proliferation appear to be bystander effects largely driven by cytokine exposure.^38^^,^^39^^,^^40^ Prior cross-sectional data suggest that the use of sirolimus in ART-suppressed renal transplant recipients with HIV may be associated with lower peripheral total CD4^+^ T cell HIV-1 DNA levels.^14^ Prospective trials designed to investigate the impact of mTOR inhibition on HIV persistence and the mechanisms by which reservoir reduction may be achieved are lacking.

We therefore conducted a phase 1/2, single-arm clinical trial to evaluate the effect of 20 weeks of continuous oral sirolimus use on HIV-specific immune responses, residual HIV-1 transcriptional activity, viral reservoir size, and immune phenotypes in PWH receiving long-term antiretroviral therapy (Advancing Clinical Therapeutics Globally [ACTG] protocol A5337; NCT02440789). Given that the main objective of this study was to assess the biological activity of the study drug, the protocol-defined, pre-specified primary analyses were based on the as-treated population, limited to participants who completed the full 20 weeks of study drug.

## Results

### Study overview and enrollment

**Figure 1:**
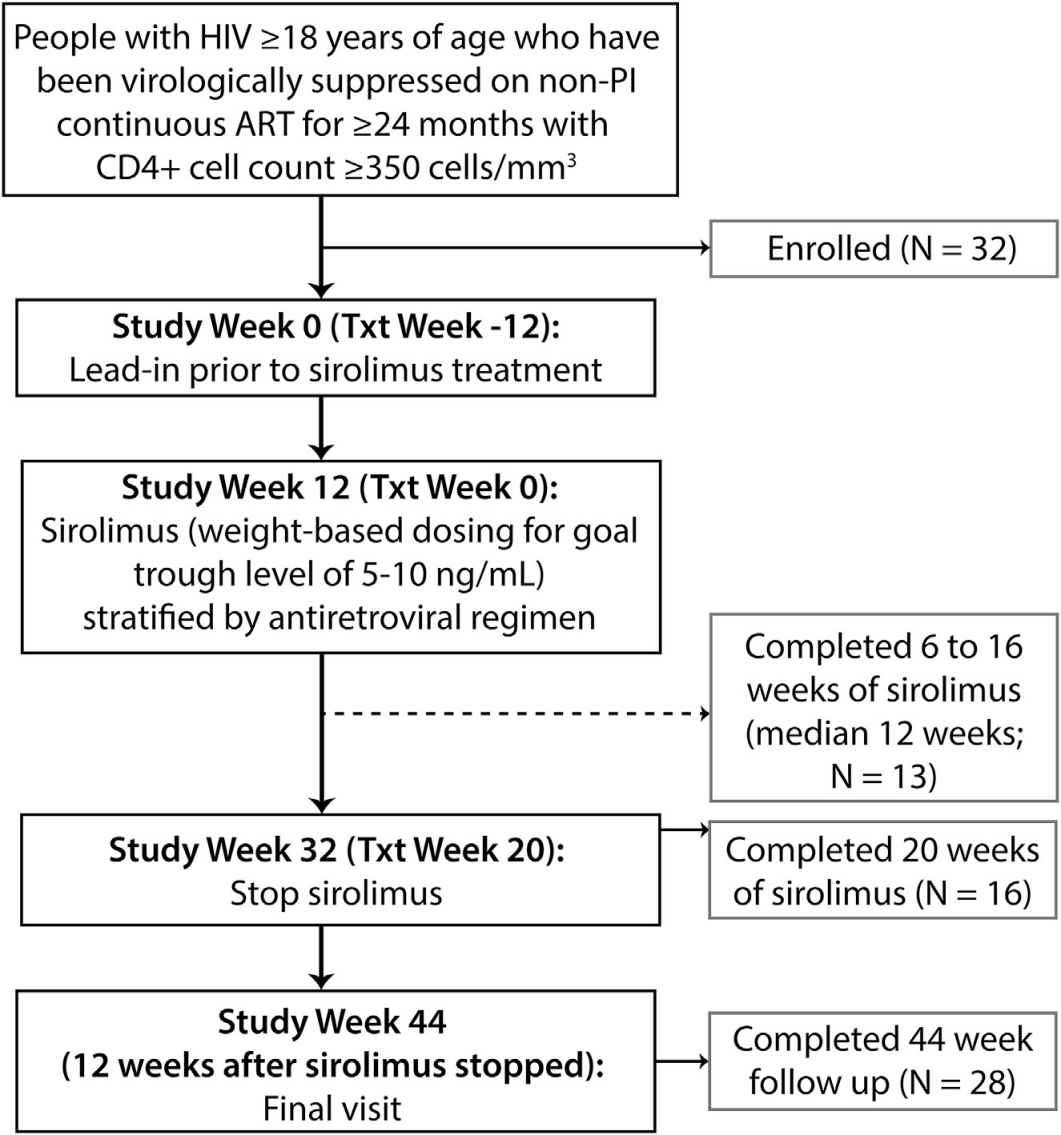
ACTG A5337 study schema and enrollment Study and treatment weeks are shown with the number of participants enrolled and completing trial milestones. Sixteen participants completed the full 20 weeks of planned oral sirolimus and are defined as the primary analysis population, and 13 participants completed 6 to 16 weeks of sirolimus (median 12 weeks) and are defined as the secondary analysis population. Twenty-nine participants completed at least 6 weeks of study drug and comprise the secondary analysis population.

Eligible persons were on ART for at least 24 months with CD4^+^ T cell counts ≥350 cells/uL and no history of systemic malignancy or recent immunomodulator use. Participants were followed for 12 weeks prior to initiating sirolimus to establish baseline levels of HIV reservoir and immunological stability. Participants then received oral sirolimus, dosed to achieve a trough plasma level of 5–10 ng/mL, which is within the range for the prevention of solid organ transplant rejection and prophylaxis against graft-versus-host disease following allogeneic stem cell transplantation (Figure 1). After treatment week 20, sirolimus was discontinued, and participants remained in the study for an additional 12 weeks to measure the longer-term impact of mTOR inhibition on HIV burden, immune responses, and inflammation.

The primary endpoints were: (1) safety, (2) changes in the frequency (%) of HIV-1 Gag-specific CD8^+^ cells after 20 weeks of sirolimus therapy, and (3) changes in CD4^+^ T cell-associated unspliced HIV-1 RNA and plasma HIV-1 RNA by the integrase single-copy assay (iSCA) from baseline to week 20 of sirolimus therapy. Secondary endpoints included changes in total cell-associated HIV-1 DNA levels, HIV-1-specific CD4^+^ T cell responses, and markers of T cell activation and cycling. As pre-defined in the study protocol, primary analyses involved the as-treated population, given that this early-phase study was conducted to assess the biological activity of mTOR inhibition in PWH on ART. Baseline participant characteristics, including demographics, ART regimen, baseline CD4^+^ T cell counts, weight, and plasma HIV-1 RNA measurements are listed in Table 1. Overall, 28% of enrolled participants were female and 72% were non-White. The median age was 52 and median CD4^+^ T cell count was 813 cells/μL. Forty-four percent of participants were on an integrase inhibitor-based regimen with the remainder on a non-nucleoside reverse transcriptase inhibitor (NNRTI)-based regimen. No participants were on protease inhibitor-based regimens as per protocol, given the significant pharmacological interactions with sirolimus.

**Table 1:** Entry and baseline characteristics of study populations

Characteristic	All enrolled (*N* = 32)	Included in safety analysis (*n* = 30)	Primary efficacy population^a^ (*n* = 16)	Secondary efficacy population^b^ (*n* = 13)
**Age at study entry**				

Median (Q1, Q3)	52 (46, 55)	52 (46, 55)	52 (43, 59)	52 (46, 53)

**Female gender**				

*N* (%)	9 (28%)	7 (23%)	3 (19%)	3 (23%)

**Race/Ethnicity**				

White Non-Hispanic	9 (28%)	8 (27%)	6 (38%)	2 (15%)
Black Non-Hispanic	18 (56%)	18 (60%)	7 (44%)	10 (77%)
Hispanic	4 (13%)	3 (10%)	2 (13%)	1 (8%)
More than one race	1 (3%)	1 (3%)	1 (6%)	0 (0%)

**IV drug history**				

Never used	29 (91%)	27 (90%)	14 (88%)	12 (92%)
Previously	3 (9%)	3 (10%)	2 (12%)	1 (8%)

**Entry HIV plasma RNA**				

≥40 copies/mL	0 (0%)	0 (0%)	0 (0%)	13 (100%)

**Txt week 0 HIV plasma RNA**				

≥40 copies/mL	1 (3%)	1 (3%)	0 (0%)	1 (8%)

**Baseline CD4^+^ T cell count (c/mm^3^)**				

Median (Q1, Q3)	813 (618, 1,014)	818 (635, 1,031)	846 (662, 1,065)	765 (635, 972)

**Baseline CD4/CD8^+^ ratio**				

Median (Q1, Q3)	0.9 (0.7, 1.3)	0.9 (0.7, 1.3)	0.9 (0.7, 1.4)	1.0 (0.8, 1.3)

**Baseline weight (kg)**				

Median (Q1, Q3)	83 (68, 99)	83 (68, 99)	79 (64, 93)	90 (77, 100)

**Baseline BMI (kg/m^2^)**				

Median (Q1, Q3)	27 (23, 31)	27 (23, 30)	24 (22, 29)	29 (26, 31)

**Entry ARV regimen**				

Non-PI, non-NNRTI (INSTI) based	14 (44%)	13 (43%)	9 (56%)	4 (31%)
Non-PI, RPV based	3 (9%)	2 (7%)	1 (6%)	1 (8%)
Non-PI, other NNRTI based	15 (47%)	15 (50%)	6 (38%)	8 (62%)^c^

IV, intravenous; BMI, body mass index; ARV, antiretroviral; PI, protease inhibitor; NNRTI, non-nucleoside reverse transcriptase inhibitor; INSTI, integrase strand transfer inhibitor; RPV, rilpivirine. Baseline is the average of pre-sirolimus measurements at study weeks 0 and 12.

a Includes participants who completed the full 20 weeks of sirolimus.

b Includes participants who completed 6 to 16 weeks of sirolimus.

c Participants in the primary efficacy population had lower efavirenz (NNRTI) use than in secondary efficacy population (38% versus 62%).

### Sirolimus therapy in participants with HIV on ART

A total of 32 participants across 10 sites were enrolled into A5337 between December 2015 and March 2017. Twenty-eight participants completed the full 44 weeks of study follow-up (Figure 1). Two participants withdrew from the study prior to the initiation of sirolimus, and two discontinued study 2 and 16 weeks after starting the drug (not able to attend clinic and withdrawal of consent, respectively). Sixteen participants completed the full 20 weeks of sirolimus and comprised the primary efficacy analysis population. An additional thirteen participants completed 6 to 16 weeks of sirolimus therapy (median 12 weeks) and were grouped into a secondary efficacy analysis population. Overall, participants received a median of 19.1 weeks of sirolimus (1.0–21.6 weeks). In contrast to all enrolled participants, 56% of the primary efficacy analysis population was on non-NNRTI-based antiretroviral regimen and there was a greater percentage of White, non-Hispanic participants (38%) (Table 1). Participants in the secondary efficacy analysis population (i.e., those unable to complete the full course of therapy) had a higher weight and BMI, lower absolute CD4^+^ T cell counts, and a higher rate of efavirenz use as shown in Table 1.

### Safety analyses

Thirty participants received at least one dose of sirolimus and comprised the study-defined primary safety analysis population. No participants experienced protocol-defined virologic failure (confirmed plasma HIV-1 RNA ≥200 copies/mL). Of the 30 participants who initiated sirolimus, 20 had an adverse event judged related to sirolimus (the maximum grades were 1, 2, and 3 for 4, 13, and 2 individuals, respectively; one had a non-graded non-fasting triglyceride event; 12 of these 20 completed the full 20 weeks of sirolimus). Lower grade toxicities that did not lead to treatment discontinuation were predominantly related to increases in fasting blood glucose levels or abnormalities in the fasting lipid panel (*e.g*., increased triglycerides). These laboratory abnormalities are known effects of sirolimus.^8^^,^^9^

Twenty participants had a treatment-related safety event, but only two were graded as severe. Fourteen participants prematurely discontinued study treatment (Table S1). Whereas the discontinuation rate was higher than expected, a majority of participants who stopped therapy did so for reasons other than protocol-defined adverse events (e.g., personal choice or on the recommendation of their medical provider). Three participants met the pre-specified primary safety outcome. Two participants experienced a grade 3 adverse event related to sirolimus (stomatitis and elevated fasting glucose), and one had a confirmed CD4^+^ T cell count decrease to below 300 cells/μL.

### Pharmacokinetic measurements and dosing

Oral sirolimus use requires frequent monitoring in order to establish and maintain trough levels within the desired range. Throughout the study, the median within-participant average trough sirolimus level was 6.8 ng/mL (range 5.4–7.8) over a median 11 measurements for each participant in the primary efficacy analysis population. The therapeutic goal was 5–10 ng/mL for sirolimus trough levels during the first 4 weeks of therapy as participants were achieving steady-state concentrations for the primary and secondary analysis populations. Interestingly, participants in the primary efficacy population had higher time-averaged trough levels than in the secondary analysis population (6 ng/mL versus 5.1 ng/mL).

### Impact of sirolimus on CD4+T cell counts

**Figure 2:**
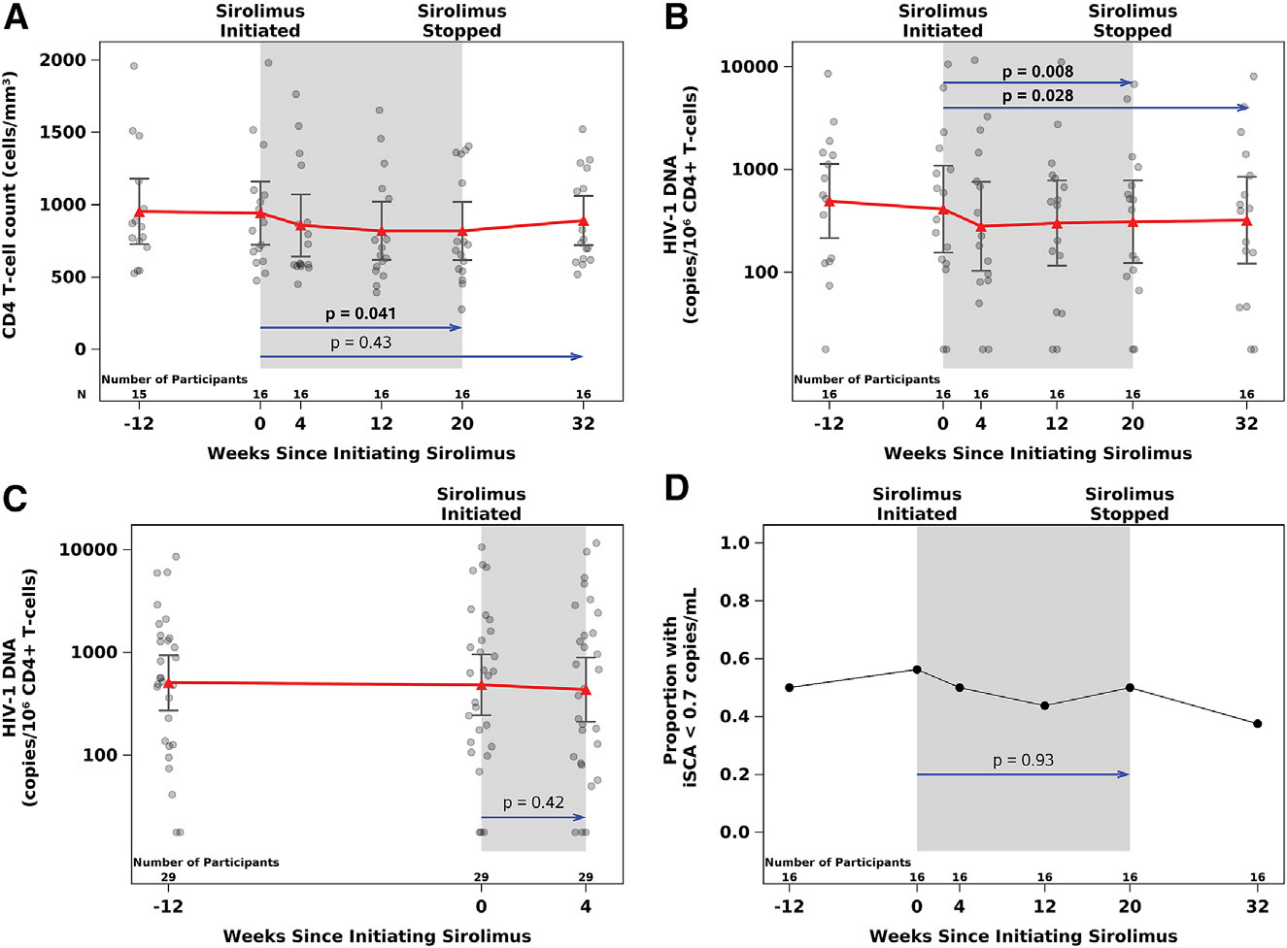
Changes in CD4^+^ T cell counts, CD4^+^ T cell-associated HIV-1 DNA, and low-level residual plasma HIV-1 RNA in response to sirolimus treatment Transient decreases in CD4^+^ T cell counts (A) and sustained and significant reduction in CD4^+^ T cell-associated HIV-1 DNA (B) in the primary analysis population are shown. CD4^+^ T cell-associated HIV-1 DNA at baseline and week 4 time points in the secondary analysis population is shown in (C). HIV-1 DNA levels remain significantly decreased 12 weeks after discontinuation of sirolimus in the primary efficacy population. The proportion of participants with undetectable low-level residual HIV-1 plasma RNA measured by iSCA for the primary analysis population is shown in (D). 95% confidence intervals and means at each time point are presented. *p* values from two-tailed, paired t tests. Baseline values were defined as the average of measures from treatment weeks −12 and 0 in statistical analyses.

A modest and transient decrease in CD4^+^ T cell counts was observed in the safety analysis population (*n* = 30; median decline of 37–91 cells/μL at treatment weeks 2–20 relative to a median baseline CD4^+^ T cell count of 818 cells/μL). In the primary efficacy population (*n* = 16 who completed 20 weeks of oral sirolimus), there was a significant decline in CD4^+^ (mean: −118 cells/μL; *p* = 0.041) and CD8^+^ (mean: −160 cells/μL; *p* = 0.021) T cell counts between baseline and treatment week 20 by paired t tests, but no change in CD4/CD8 ratios. In the combined primary and secondary efficacy populations, CD4^+^ T cell counts decreased by a mean of 60 cells/μL (*p* = 0.06) from baseline to treatment week 4 (*n* = 29). CD4^+^ T cell counts in the primary efficacy population decreased from baseline to treatment week 4 (−79 cell cells/μL), and CD4^+^ T cell counts declined by an average of 37 cells/μL from baseline to treatment week 4 in the secondary analysis population (*n* = 13). However, CD4^+^ T cell counts increased 12 weeks following cessation of sirolimus and were not significantly different at the post-treatment time point from baseline values in either population as shown in Figure 2A.

### Antigen-specific T cell responses

To test the effect of sirolimus treatment on viral antigen-specific T cell responses, flow cytometric evaluation of intracellular cytokine responses (IFNγ, IL-2, TNF-α, and MIP-1β) and cell surface markers (CD40L and CD107a) following a 6 h stimulation of peripheral blood mononuclear cells in the presence or absence of overlapping HIV-1 Gag peptide pools, CMV (pp65) antigen, and Staphylococcal enterotoxin B (SEB; positive stimulation control) was performed as shown in Figure S1. Overall, no significant changes were observed in the frequency of Gag-specific CD8^+^ or CD4^+^ T cells expressing any marker from baseline to treatment week 20 in the primary efficacy analysis population or between baseline and week 4 in the secondary efficacy population.

### Impact of sirolimus on cell-associated HIV-1 DNA and RNA

Cell-associated (CA) DNA and RNA were measured at baseline, treatment weeks 4, 12, and 20, and 12 weeks after treatment was discontinued (week 32) (Figure 2B; Table S3). Although CD4^+^ T cell-associated unspliced HIV-1 RNA levels decreased from baseline to week 20 in the primary efficacy population, this change did not reach statistical significance (mean: −0.21 log_10_ copies/10^6^ cells, *p* = 0.11). In the primary efficacy population (*n* = 16), there was a significant decrease in CD4^+^ T cell-associated HIV-1 DNA from baseline to treatment week 20 (mean DNA decline of 0.16 log_10_ copies/10^6^ cells, *p* = 0.008), which corresponds to a 31% decline (Figure 2B). The lower levels of CA-DNA persisted after sirolimus discontinuation resulting in a persistent decrease in CD4^+^ T cell-associated HIV-1 DNA (mean −0.15 log_10_ copies/10^6^ cells; *p* = 0.028; primary efficacy population) from baseline to week 32 (12 weeks following cessation of sirolimus). Overall, the magnitude of mean change in CD4^+^ T cell-associated HIV-1 DNA was similar between baseline and all treatment time points with a borderline significant mean change in HIV-1 DNA levels from baseline to treatment week 4 being observed in the primary efficacy population (mean −0.21 log_10_ copies/10^6^ cells; *p* = 0.05) but not from baseline to week 12 (mean: −0.17 log_10_ copies/10^6^ cells; *p* = 0.13) as shown in Table S3. There were no significant changes in CA-DNA from baseline to treatment week 4 in the secondary efficacy population as shown in Figure 2C.

To further understand the impact of sirolimus on total HIV-1 DNA levels, a linear mixed-effects model was applied to all on-treatment time points (weeks 4, 12, 20) together with the pre-treatment data, to estimate change from baseline, combining the primary and secondary analysis populations (*n* = 29 contributing to the treatment week 4 time point and *n* = 16 contributing to the weeks 12 and 20 time points); the estimated log_10_ change in CD4^+^ T cell-associated HIV-1 DNA was −0.08 (*p* = 0.22). Applying this model restricted to the primary analysis population (*n* = 16), the estimated log_10_ change was −0.18 (*p* = 0.026).

### Impact of sirolimus on residual low-level plasma HIV-1 RNA

Low-level plasma HIV-1 RNA was measured using iSCA, which had a lower limit of detection of 0.7 copies/mL. Among the primary efficacy population, 46 of 96 (48%) total test results were below this limit. There were no apparent changes in the frequency of detectable plasma RNA as shown in Figure 2D, and similarly, no evidence of a change in the frequency of detectable plasma RNA up to treatment week 4 in the secondary efficacy analysis population. Due to a high percentage of iSCA results less than the analysis lower limit, a supplemental analysis accounting for left-censoring was performed for the change in iSCA from baseline to treatment week 20 (primary efficacy population) and from baseline to treatment week 4 (secondary efficacy population). Results were consistent with those from the paired t tests as aforementioned, with no evidence of changes in iSCA levels after sirolimus treatment.

### Impact of sirolimus on intact proviral DNA

The intact proviral DNA assay (IPDA) was performed as described^41^ at baseline (treatment week 0), treatment week 4, and treatment week 20 in participants in the primary efficacy population (*n* = 16). Many of these participants had no detectable intact proviral DNA at treatment week 0 (56%), treatment week 4 (47%), and treatment week 20 (69%, the primary treatment endpoint). Participants who had detectable intact proviral DNA at any time point were included in a descriptive analysis of the change in the log_10_-transformed intact proviral DNA copies/10^6^ CD4^+^ T cells as previously reported for analyses using the IPDA.^41^ At treatment week 4, 67% (6 of 9) of evaluated participants had decreased intact proviral DNA (median change of −0.14 log_10_, *n* = 9), and 75% (6 of 8) of evaluated participants had decreased intact proviral DNA (median change of −0.40 log_10_, *n* = 8) at treatment week 20.

### Markers of T cell exhaustion, memory, activation, and cycling

Changes in markers of T cell activation (CD69), exhaustion (PD-1), naive and memory cell phenotypes (naive, central memory, effector memory, terminally differentiated), cycling/proliferation (Ki67), and CCR5 expression between baseline and week 20 for the primary efficacy population are shown in Table 2. The frequency of CD4^+^ and CD8^+^ T cells expressing Ki67+ and CD8^+^ T cells expressing CCR5 and PD-1 significantly decreased between baseline and week 20 in the primary efficacy population (all *p* ≤ 0.031; Table S2). Of note, the significant reduction in the frequency of Ki67-expressing T cells was no longer observed 12 weeks after discontinuation of sirolimus. In analyses of T cell phenotyping in the secondary efficacy population, a significant reduction in the frequency of the naive CD4^+^ T cells between baseline and week 4 (*p* = 0.015) was also observed.

**Table 2:** Change in percentage of T cell markers and subsets from baseline to sirolimus treatment week 20 in the primary efficacy population (*N* = 16)

	CD8^+^ T cells		CD4^+^ T cells	
	Mean (CI)	*p*	Mean (CI)	*p*
% PD-1+	−2.85 (−4.85, −0.86)	0.008^a^	0.42 (−1.81, 2.66)	0.69
% Ki67+	−0.54 (−0.90, −0.19)	0.005	−0.51 (−0.97, −0.05)	0.031
% CCR5+	−3.92 (−5.99, −1.85)	0.001	−1.67 (−3.76, 0.42)	0.11
% CD69^+^	−0.52 (−1.38, 0.35)	0.22	−0.17 (−0.97, 0.63)	0.65
% T_Naïve_	0.75 (−3.61, 5.12)	0.72	−2.16 (−5.46, 1.15)	0.18
% T_CM_	−0.87 (−1.82, 0.08)	0.07	−0.01 (−2.18, 2.17)	1.00
% T_EM_	−0.58 (−3.02, 1.86)	0.62	2.20 (−0.50, 4.90)	0.10
% T_TD_	0.68 (−2.82, 4.18)	0.69	−0.04 (−1.21, 1.14)	0.95

T_CM_, central memory; T_EM_, effector memory; T_TD_, terminally differentiated.

a From two-tailed, paired t tests.

### Soluble marker of inflammation and immune activation

**Figure 3:**
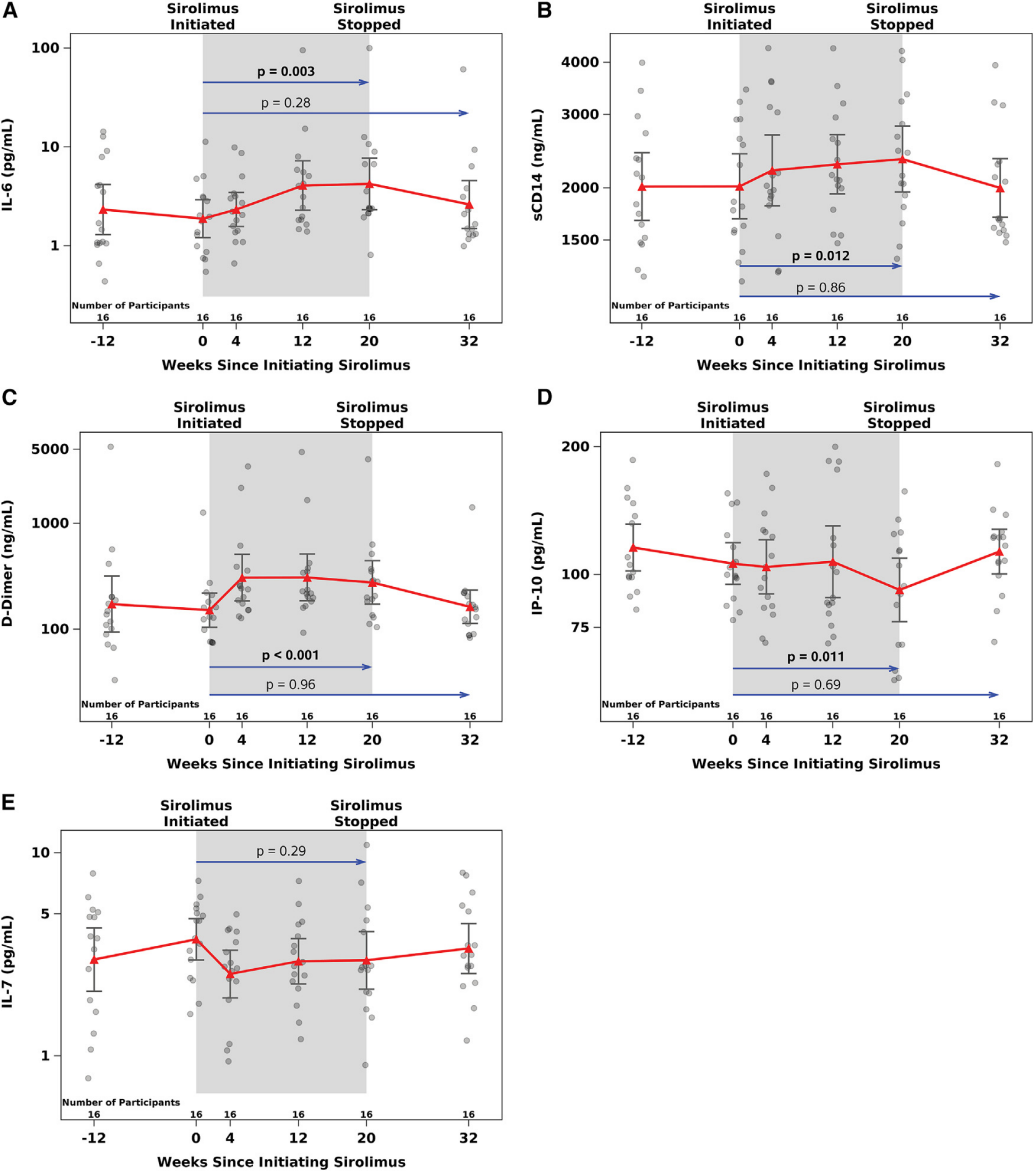
Changes in soluble markers of inflammation in response to sirolimus treatment Significant increases in circulating IL-6, sCD14, and D-Dimer are shown in (A)–(C). Levels returned to baseline 12 weeks after the cessation of treatment. A significant decrease in interferon gamma-induced protein 10 (IP-10) levels was observed at treatment week 20, which return to baseline 12 weeks after cessation of sirolimus therapy (D). IL-7 levels for the primarily analysis population are shown in (E). 95% confidence intervals and geometric means at each time point are presented. *p* values are from two-tailed, paired t tests. Baseline values were defined as the average of measures from treatment weeks −12 and 0 in statistical analyses.

To evaluate the longitudinal impact of sirolimus treatment on inflammation, we quantified changes in plasma IL-6, IL-7, IP-10, sCD14, and D-dimer using marker-specific ELISA at sirolimus treatment weeks −12, 0, 4, 12, 20, and 32 (12 weeks following cessation of therapy) as shown in Figure 3. Pro-inflammatory markers IL-6 (mean change: 0.31 log_10_; *p* = 0.003), sCD14 (mean change: 0.07 log_10_; *p* = 0.012), and D-Dimer (mean change: 0.23 log_10_; *p* = <0.001) increased significantly from baseline to treatment week 20, whereas IP-10 significantly decreased (mean change: −0.08 log_10_; *p* = 0.01) from baseline to treatment week 20 in the primary efficacy analysis population. In the secondary efficacy population, sCD14 (mean change: 0.05 log_10_; *p* = 0.002) and D-Dimer (mean change: 0.25 log_10_; *p* < 0.001) increased from baseline to treatment week 4. However, no change was evident in the soluble biomarkers of inflammation from baseline to treatment week 32, 12 weeks following cessation of sirolimus. As expected, with the normalization of these markers after discontinuation of sirolimus, all showed evidence of a significant change between treatment week 20 and 12 weeks following cessation of the drug in the opposite direction of those observed from baseline to treatment week 20 as shown in Figure 3.

### Correlation between sirolimus drug levels, immune phenotyping, markers of inflammation, and outcome measures

Overall, there were no significant correlations between sirolimus trough levels and changes to treatment week 20 in CA-RNA or DNA levels, soluble markers of inflammation, or percentages of CD4^+^ and CD8^+^ T cells expressing Ki67, CCR5, or PD-1. However, a positive correlation between sirolimus levels and changes in CD4^+^ T cell counts (Spearman r = 0.55; *p* = 0.027) was observed. There were also no significant correlations between the change in percentage of total CD4^+^ T cells, CD4^+^ or CD8^+^ T cells expressing Ki67, and CD8^+^ T cells expressing PD-1 with changes in HIV-1 DNA, CA-RNA, or total CD4^+^ T cell counts from baseline to treatment week 20 in the primary efficacy analyses. Similarly, there were no significant associations between changes in circulating levels of IL-6, sCD14, IP-10, and D-Dimer with changes in HIV-1 DNA, CA-RNA, or total CD4^+^ T cell counts in the primary efficacy population (Table S4).

## Discussion

Despite years of research to achieve long-term HIV remission, few interventions have reduced cell-associated HIV-1 DNA levels in PWH who are receiving effective ART. Whereas allogeneic stem cell transplantation and initiation of ART during “hyperacute” infection are associated with very low reservoir sizes,^42^^,^^43^ these strategies are not applicable to the vast majority of people living with HIV. Based on data from multiple groups that memory T cell clonal proliferation is a major mechanism for HIV persistence during long-term ART,^28^^,^^29^^,^^30^^,^^31^^,^^32^^,^^33^^,^^34^^,^^35^ we postulated that inhibition of proliferation might result in a reduction in reservoir size, a hypothesis predicted by recent modeling.^4^ Here, we tested the hypothesis that inhibition of mTOR would reduce the size of the reservoir, as estimated by CD4^+^ T cell-associated HIV-1 DNA, and other measures. We chose to study mTOR inhibitors based on prior hypothesis-generating studies performed by our group,^14^^,^^44^ and based on the observations that mTOR inhibition might reduce cytokine-driven homeostatic proliferation.^45^^,^^46^ Although the sample size was small and the study was complicated by a larger-than-expected frequency of premature treatment discontinuations, we found evidence that sirolimus treatment was associated with a modest reduction in cell-associated DNA levels persisting up to 12 weeks after cessation of the drug. We also found that mTOR inhibition reduced cell cycling (as defined by Ki67) in CD4^+^ and CD8^+^ T cells, reduced CCR5 expression on CD8^+^ T cells, and may have reduced T cell exhaustion (as evidenced by reduced PD-1 expression on effector cells).

Most of the HIV proviruses are defective.^47^ As both defective and intact genomes are maintained by homeostatic proliferation, the effect of non-specifically inhibiting proliferation of all memory cells should result in similar reductions of defective and intact genomes. Still, given that the intact and potentially replication-competent reservoir is the main focus of most cure interventions, we estimated the size of the intact reservoir before and after sirolimus using the IPDA. Most participants had no readily detectable intact genomes, due in part to the limited number of cells available. Despite these limitations, we observed a median 0.40 log_10_ decrease in intact HIV DNA over the 20-week treatment time course in those with detectable levels at any visit. A prior study of the impact of ART alone on intact proviral DNA (ACTG A5321) showed a median half-life of 7.1 years, which corresponds to a −0.016 log_10_ change over 20 weeks.^48^ Results from A5321 also demonstrate that change in total DNA on ART has a much slower rate of decline (half-life of 41.6 years) highlighting the importance of the observed reductions in total HIV DNA on sirolimus,^48^ and that the decay in intact proviral DNA on study drug may be an order of magnitude greater than natural decay of the reservoir on ART.

These data support preliminary observations from a prior retrospective cross-sectional study of solid organ transplant recipients, in which persons treated with sirolimus had an approximately 0.3 log_10_ copies/mL lower level of HIV-1 DNA than did persons receiving other immune-modulating drugs (e.g., cyclosporine and tacrolimus).^14^ This modest effect is consistent with the effect observed in our longitudinal study and larger than an expected ∼2% decline over 20 weeks observed on stable ART.^49^ We also observed in a recent pilot study of 10 PWH on ART, who switched to or added everolimus (an mTOR inhibitor with broad TORC1/TORC2 activity) for graft rejection following solid organ transplantation, that it did not have an overall effect on cell-associated HIV-1 DNA and RNA levels. However, participants who maintained everolimus time-averaged trough levels >5 ng/mL during the first 2 months of therapy had significantly lower CD4^+^ T cell HIV RNA levels up to 6 months after the cessation of study drug.^44^ Time-averaged everolimus trough levels significantly correlated with greater inhibition of mTOR gene pathway transcriptional activity. In this study, there was an absence of a clear pharmacodynamic relationship between sirolimus exposure and outcome measures, but there was limited variation in drug exposure between participants as intensive monitoring and adjustment of sirolimus levels were incorporated.

The sustained reduction in CD4^+^ T cell-associated HIV-1 DNA levels was limited to our primary analysis population who completed all 20 weeks of daily sirolimus treatment. Although we observed a modest decrease in cell-associated HIV-1 DNA in the remaining 13 participants who completed 6–16 weeks of sirolimus, these results were not statistically significant. Interestingly, these participants tended to have higher weight and BMI, lower CD4^+^ and CD8^+^ T cell counts, and higher rates of efavirenz use at baseline. Importantly, these participants also had lower time-averaged sirolimus trough levels while on study drug, perhaps related to challenges with achieving stable trough drug levels with weight-based dosing in those with higher BMI, which may have subsequently played a role in HIV-1 DNA response differences from those that completed the 20 planned weeks of treatment.

Several factors may have led to the observed sustained reductions in peripheral blood CD4^+^ T cell-associated HIV-1 DNA levels in the primary analysis population. Firstly, Ki67, an intracellular marker of T cell cycling, was significantly reduced during the period of oral sirolimus use. Homeostatic proliferation plays an important, if not the main, role in maintaining the latent HIV reservoir on ART.^28^^,^^29^^,^^30^^,^^31^^,^^32^^,^^33^^,^^34^^,^^35^ Blocking the proliferation of HIV-infected CD4^+^ T cells might prove to be an important target for HIV cure research,^4^ perhaps used as a combination approach.^45^^,^^50^ If CD4^+^ T cell cycling is largely driven by a cytokine-enriched environment as suggested,^51^^,^^52^ blocking the proliferation of cells comprising the latent reservoir would be expected to reduce HIV DNA burden over time, particularly if the virus is enriched in cells with high proliferative potential, as appears to be the case.^33^^,^^53^ Clinical trials designed to interrupt proliferation or inflammatory signaling (e.g., by administration of mycophenolate, JAK-STAT inhibitors)^4^^,^^54^ may decrease reservoir size. Unlike calcineurin inhibitors (cyclosporine and tacrolimus), which work by blocking T cell receptor-mediated immune signaling, mTOR inhibitors that block cytokine-mediated stimulation may preferentially lead to decreased homeostatic or cytokine-driven proliferation while preserving antigen-specific immune responses.^37^^,^^55^^,^^56^^,^^57^ While CD4^+^ T cell numbers fell slightly, the disproportionate decrease in frequency of CD4^+^ T cells containing HIV DNA in this study suggests that CD4^+^ T cells and the offspring of CD4^+^ T cells that were susceptible to HIV infection (irrespective of the replication competence of the provirus) remain selectively driven to proliferation and expansion by cytokine exposure.

Whereas the observed decrease of circulating HIV-1 DNA in this study may have been due, in part, to CD4^+^ T cell redistribution to tissues outside peripheral blood, a recent modeling study suggests that reduced CD4^+^ T cell cycling and homeostatic proliferation are likely to decrease infected cell burden in both blood and tissues^4^ through blocking memory CD4^+^ T cell generation from HIV-uninfected precursor cells.^51^ A recent non-human primate study showing decreases in simian immunodeficiency virus (SIV) DNA following antibody-mediated CD4^+^ depletion^58^ lends support to this putative homeostatic-based mechanism for the sirolimus effect on HIV-1 DNA, fundamentally different than the shock-and-kill therapeutic approach.^52^ It is interesting to note that the secondary efficacy population in this study that did not experience significant reductions in CD4^+^ T cell-associated HIV-1 DNA also did not have decreased markers of T cell cycling at the earlier 4-week time point after initiating sirolimus treatment.

Interestingly, a recent non-human primate study of sirolimus therapy with or without CD3^+^ T cell depletion did not demonstrate a decrease in overall HIV burden or lead to changes in time to SIV rebound following analytical treatment interruption (ATI), but did demonstrate decreased memory CD4^+^ T cell proliferation.^50^ The reason for the discrepancy between reservoir decay in the SIV model and the significant reductions observed in this human study is not known, but could be due to the duration of ART. In our clinical study, we enrolled individuals who had been on long-term ART. The reservoir is increasingly found in the clonal populations over time,^29^^,^^30^^,^^53^^,^^59^ and hence any agent aimed at this mechanism might only become readily detected after many years of virus suppression.

Sirolimus use also led to reduced expression of PD-1 and CCR5 on CD8^+^ T cells through week 20 of sirolimus (in the primary efficacy population). The PD-1/PD-L1 pathway is implicated in the balance between immune eradication and immune escape and is overexpressed in chronic viral infections such as HIV, even after the initiation of ART.^60^^,^^61^^,^^62^^,^^63^ As a result, there is interest in using anti-PD-1 therapies to reduce the latent HIV reservoir burden.^64^^,^^65^^,^^66^ The role of decreased frequencies of CD8^+^ T cells expressing PD-1 observed in this study is not known and warrants further study, especially as some SEB-specific responses declined with mTOR inhibition and there were no significant changes in HIV-specific responses.

Very few studies have performed a comprehensive assessment of mTOR inhibition in people. As expected, we found that sirolimus had pluripotent effects. We observed changes in cell proliferation (Ki67) and activation (CCR5 and PD-1) as expected, although likely due to small samples size, the effect was significant in only some subsets. We observed a decrease in IP-10, a marker of the interferon response pathway.^67^ Surprisingly, we observed a significant increase in circulating markers of inflammation, IL-6, d-Dimer, and sCD14, all of which in untreated and treated HIV predict excess risk of morbidity.^68^^,^^69^^,^^70^^,^^71^^,^^72^^,^^73^^,^^74^ Data regarding the *in vivo* impact of sirolimus on IL-6 production is evolving, with evidence that mTOR inhibition may reduce IL-6 expression by various antigen-presenting cells, but may also increase expression by others.^75^^,^^76^^,^^77^ Importantly, D-dimer levels also increased during sirolimus administration, and as these levels are diminished by systemic IL-6 blockade, it is likely that sirolimus administration increases IL-6 expression and bioactivity. As a result, there is some concern that mTOR inhibition may exacerbate pre-existing, persistent HIV-related inflammation, although increases related to a therapeutic intervention will likely have unique implications that are yet to be understood. However, inflammatory markers returned to baseline levels following cessation of sirolimus, and it is possible that the transient increases in immune signaling may have enhanced immune clearance of HIV-infected CD4^+^ T cells through stimulation of innate or adaptive immune responses.

This study had several limitations. First, the ACTG A5337 study was an open-label, single-arm clinical trial and did not include a randomized placebo group, although a lead-in observation and sampling period was incorporated into the study design to help control for instability in HIV-1 reservoir measures. Also, as aforementioned, only 16 of 30 participants who initiated treatment completed the full 20 weeks. The as-treated population was the basis for primary analyses and sample size estimation, but the frequency of treatment discontinuation was unexpected, although most premature discontinuations were due to low-level events with minimal clinical implications and were driven by either strict protocol definitions or site clinical preference rather than by participant-experienced adverse events. A recent study of everolimus in HIV-infected individuals who underwent solid organ transplant demonstrated that mTOR inhibitor usage in real-world clinical situations was well tolerated without any reported adverse events,^44^ with the caveat that this trial had greater tolerance for mild adverse event and laboratory abnormalities, as the drug was being used for an approved clinical indication. Furthermore, low-dose sirolimus is now being trialed in various clinical settings to minimize these adverse events while preserving desired immune-modulating functions.^78^^,^^79^ Thus, it may play a role in combination HIV cure strategies, including ones that aim to reduce the proliferative survival of infected cells. Regardless of study dropout, decreases in HIV DNA and changes in inflammatory and immune phenotypes reached statistical significance in this primary analysis population. Furthermore, the study was an open-label, single-arm trial given the need for frequent adjustments of sirolimus dosing and the logistical challenges of including a control group. The 12-week lead-in observation period prior to sirolimus therapy allowed us to determine the stability of the HIV reservoir and baseline immune markers.

In conclusion, this study of mTOR inhibitors in otherwise healthy PWH on long-term, suppressive ART revealed several important significant effects of sirolimus therapy, such as decreasing peripheral blood HIV-1 DNA burden, CD4^+^ T cell cycling, and CD8^+^ T cell PD-1 expression, which suggest that immune modulatory therapies may play an important role in ongoing efforts to cure HIV infection in persons with established infection.

### Limitations of the study

The ACTG A5337 study was an open-label, single-arm clinical trial and did not include a randomized placebo group. Whereas a lead-in period was incorporated into the study design to take into account HIV-1 reservoir instability over time, incorporation of a placebo arm would have been challenging as some participants underwent frequent dose adjustments to maintain therapeutic and safe levels of sirolimus. Furthermore, the study had a relatively high non-completion rate, although many of the premature discontinuations were not based on clinically significant adverse events. As such, the study lacked statistical power to identify subtle changes in intact proviral DNA or residual low-level HIV-1 RNA at primary and other study time points.

## Resource availability

### Lead contact

Further information and requests for resources and data should be directed to Timothy J. Henrich (timothy.henrich@ucsf.edu), who will facilitate requests in coordination with the ACTG (Advancing Clinical Therapeutics Globally).

### Materials availability

The remaining biological specimens obtained from this clinical trial are maintained and stored by the ACTG. An ACTG New Works Concept Sheet may be filed by investigators who propose access to samples for additional research, which will be reviewed by the ACTG and primary A5337 study team.

### Data and code availability

No custom statistical code was designed or used in this study. All non-identifiable data and statistical code will be made available upon request. Individual values are shown in each figure for flow cytometric, ELISA, and PCR assay results. Any additional information required to reanalyze the data reported in this paper is available from the lead contact upon request.

## Acknowledgments

Research reported in this publication was supported by the 10.13039/100000060National Institute of Allergy and Infectious Diseases of the 10.13039/100000002National Institutes of Health under award number UM1 AI068634, UM1 AI068636, UM1 AI106701, UM1 AI069496-08, UM1 AI069412, and K24AI174971-01A1 (to T.J.H.) and 10.13039/100010877ViiV Healthcare. The content is solely the responsibility of the authors and does not necessarily represent the official views of the National Institutes of Health. ClinicialTrials.gov registration #: NCT02440789.

## Author contributions

T.J.H.: A5337 study co-chair, clinical trial design and implementation, experiment design and analysis, and wrote the manuscript. P.Y.H.: A5337 study co-chair, clinical trial design and implementation, and conceived the study. S.G.D.: A5337 vice-chair, conceived the study, and clinical trial design and implementation. C.G.: division of AIDS clinical representative, clinical trial design and implementation, and regulatory approvals. R.J.B. and H.M.: clinical trial design and implementation, and data and statistical review and analysis. A.N.: data management. E.H.: clinical trial design and regulatory approvals. M.K. and C.F.: participant enrollment, study site investigators, and clinical evaluations. D.M., B.C., J.Z.L., D.R.K., A.M.B., and M.M.L. designed and performed the experiments and analyzed the data. A.N.D. and F.A. designed and performed pharmacology analyses.

## Declaration of interests

T.J.H. received grant support from Gilead Sciences, Merck, and Bristol Myers Squibb. D.R.K. receives grant support and/or consulting honoraria from AbbVie, Gilead Sciences, GlaxoSmithKline, Janssen, Merck, Roche, and ViiV. J.Z.L. received grant support from Merck.

## STAR★Methods

### Key resources table

**Table:** 

REAGENT or RESOURCE	SOURCE	IDENTIFIER
**Antibodies**		

LIVE/DEAD Fixable Aqua Dead Cell Stain Kit	Invitrogen	Cat# L34957
Anti-CD3 BV711 Clone UCHT1	BD Biosciences	Cat# 563725; RRID: AB_2744392
Anti-CD4 BUV395 Clone SK3	BD Biosciences	Cat# 563552; RRID: AB_2738275
Anti-CD8 APC-Cy7 Clone SK1	BioLegend	Cat# 344714; RRID: AB_2044006
Anti-IFN-γ PE-Cy7 Clone B27	BioLegend	Cat# 506518; RRID: AB_2123321
Anti-TNF-α FITC Mab11	BioLegend	Cat# 502906; RRID: AB_315258
Anti-IL-2 APC MQ1-17H12	BioLegend	Cat# 500310; RRID: AB_315097
Anti-CD40L/CD154 24-31	BioLegend	Cat# 310802; RRID: AB_314825
Anti-MIP-1β PE D21-1351	BD Biosciences	Cat# 550078; RRID: AB_393549
Brefeldin A	BioLegend	Cat# 420601
CD107α PE-CF594 H4A3	BD Biosciences	Cat# 562628; RRID: AB_2737686

**Biological samples**		

Human peripheral blood mononuclear cells	Human participants	This manuscript
Human plasma	Human participants	This manuscript

**Chemicals, peptides, and recombinant proteins**		

HIV-1 PTE Gag Peptide Set	NIH AIDS Reagent Program	Cat# 11554
HCMV pp65 Peptide Pool	NIH AIDS Reagent Program	Cat# 11549
Cytofix/Cytoperm	BD Biosciences	Cat# 554722
SEB	Toxin Technology Inc.	Cat# NC9442400
RPMI1640	Gibco	Cat# 11875101
Fetal Calf Serum	Sigma	Cat# F4135
Benzonase	EMD Millipore	Cat# 1.01695.0001
L-glutamine	Gibco	Cat# 25030081
Penicillin and Streptomycin	Gibco	Cat# 15140122
Quantikine ELISA kit	R&D Systems	Cat# DC140
Quantikine ELISA kit	R&D Systems	Cat# HS600C
Quantikine ELISA kit	R&D Systems	Cat# HS750
AllPrep DNA/RNA Mini Kit	Qiagen	Cat# 80204
ddPCR Supermix for probes (no dUTPS)	Bio-Rad Laboratories	Cat#186-3024

**Critical commercial assays**		

Abbott RealTime HIV-1 assay	Abbott	https://www.molecular.abbott/us/en/products/infectious-disease/realtime-hiv-1-viral-load
Roche COBAS TaqMan HIV-1 Test Version 2.0	Roche	https://diagnostics.roche.com/global/en/products/params/cobas-hiv-1-test.html

**Deposited data**		

Data S1 (Study protocol and amendments)	This manuscript	This manuscript

**Experimental models: Organisms/strains**		

Human participants	A5337 clinical trial participants	This manuscript

**Oligonucleotides**		

Proviral HIV DNA/RNA Forward Primer (5′-TACTGACGCTCTCGCACC)	Integrated DNA Technologies	https://pubmed.ncbi.nlm.nih.gov/18600229/
Proviral HIV DNA/RNA Reverse Primer (5′-TCTCGACGCAGGACTCG)	Integrated DNA Technologies	https://pubmed.ncbi.nlm.nih.gov/18600229/
Proviral HIV DNA/RNA Probe (5′-FAM-CTCTCTCCTTCTAGCCTC)	Integrated DNA Technologies	https://pubmed.ncbi.nlm.nih.gov/18600229/
IPDAΨ F (5′-CAGGACTCGGCTTGCTGAAG)	Integrated DNA Technologies	https://pubmed.ncbi.nlm.nih.gov/30700913/
IPDAΨ Ψ R (5′-GCACCCATCTCTCTCCTTCTAGC)	Integrated DNA Technologies	https://pubmed.ncbi.nlm.nih.gov/30700913/
IPDAΨ Ψ Probe (5′-TTTTGGCGTACTCACCAGT)	Integrated DNA Technologies	https://pubmed.ncbi.nlm.nih.gov/30700913/https://pubmed.ncbi.nlm.nih.gov/30700913/
IPDAΨ Env F (5′-AGTGGTGCAGAGAGAAAAAAGAGC)	Integrated DNA Technologies	https://pubmed.ncbi.nlm.nih.gov/30700913/
IPDAΨ Env R (5′-GTCTGGCCTGTACCGTCAGC)	Integrated DNA Technologies	https://pubmed.ncbi.nlm.nih.gov/30700913/
IPDAΨ Env intact probe (5′-CCTTGGGTTCTTGGGA)	Integrated DNA Technologies	https://pubmed.ncbi.nlm.nih.gov/30700913/
IPDAΨ Env hypermut (5′-CCTTAGGTTCTTAGGAGC)	Integrated DNA Technologies	https://pubmed.ncbi.nlm.nih.gov/30700913/

**Software and algorithms**		

SAS version 9.4	SAS Institute	N/A
FACSDiva software version 9.0	BD Biosciences	https://www.bdbiosciences.com/en-us/products/software/instrument-software/bd-facsdiva-software
R v. 4 (lmec version 1.0)	The R Project for Statistical Computing	https://www.r-project.org/

### Experimental model and subject details

#### Study design

A5337 was a phase I/II, open-label, single-arm clinical trial to evaluate the effect of sirolimus on the HIV-reservoir size and immune function among well-suppressed individuals on antiretroviral therapy (ART). The target enrollment of this study was 30 participants ≥18 years of age and on a suppressive ART regimen (excluding PI-based or cobicistat-based regimens) for at least 24 months, with CD4^+^ cell counts ≥350 cells/μL at study screening. Participants were to complete 20 weeks of oral sirolimus following a 12-week lead period and an additional 12 weeks follow-up period following cessation of the study drug. Because the aim of this pilot study was to investigate the biologic effects of sirolimus, the pre-defined, primary analyses were as-treated, limited to subjects who have data at baseline and week 32 (20 weeks on sirolimus) and who remained on study treatment and ART (and without virologic failure) through week 32.

#### Inclusion criteria

Additional inclusion criteria included plasma HIV-1 RNA below the level of quantitation for ≥24 months by an FDA-approved assay at any US laboratory that has a CLIA certification or its equivalent. Two plasma HIV-1 RNA measurements above the limit of quantification but <500 copies/mL in the 24 months prior to screening were allowed if directly preceded and followed by HIV-1 RNA below assay limit. Females of reproductive potential who were participating in sexual activity that could lead to pregnancy must have agreed to initiate effective contraceptives before sirolimus therapy, continued and maintained use for at least 12 weeks after sirolimus therapy has been stopped. The following laboratory criteria had to have been met: white blood cell (WBC) ≥3000 cells/μL, platelet count ≥125,000/mm^3^, absolute neutrophil count >1300 cells/μL, aspartate aminotransferase (AST) < 1.25 x upper limit of normal (ULN), alanine aminotransferase (ALT) < 1.25 x ULN, calculated creatinine clearance (CrCl) ≥60 mL/min as estimated by the Cockcroft-Gault equation, fasting or non-fasting triglyceride level ≤350 mg/dL, fasting or non-fasting LDL <160 mg/dL, urine protein to urine creatinine ratio ≤1 g/g from random urine collection.

#### Exclusion criteria

Exclusion criteria included serious illness requiring systemic treatment and/or hospitalization within 30 days prior to study entry, AIDS-defining condition or oropharyngeal candidiasis within 90 days prior to study entry, intended modification to ART during the study, latent tuberculosis infection if prophylaxis had not been complete at least 48 weeks prior to study entry, active tuberculosis infection within 48 weeks of entry, history of current or active hepatitis B infection, HCV RNA+ within 90 days of study entry, history of a neoplastic disorder or clinically significant organ dysfunction prior to study entry, detectable Epstein-Barr virus in blood, any active infection requiring systemic antimicrobial therapy within 90 days of study entry, hypersensitivities to macrolide-like drugs or mTOR inhibitors, active drug or alcohol use or dependence vaccination within 14, breastfeeding, and anal or perianal administration of anti-HPV therapies 90 days prior to entry.

#### Discontinuation/Stopping criteria

Treatment was discontinued if any two consecutive CD4^+^ cell counts <300 μL or >50% decreased from study entry value, any two consecutive HIV-1 RNA levels were >200 copies/mL, participant was repeatedly noncompliant missing >3 doses of study drug a week for 2 or more weeks, participant missed two consecutive PK monitoring blood draws, or if a participant missed two consecutive clinic visits. Participants were prematurely withdrawn from the study if ART was permanently discontinued or if the participant, primary care provider or study investigator felt that the participant should stop for any reason.

#### Sirolimus dosing and drug levels

Because of drug-drug interactions between sirolimus and ritonavir and cobicistat, participants on protease-inhibitor-based therapies were excluded from the study. For participants on an ART regimen that did not include a non-non-nucleoside reverse transcriptase inhibitor (NNRTI) regimen, and for those on rilpivirine (RPV) based regimen sirolimus was initiated at 0.025 mg/kg/day initial dose and 0.05 mg/kg/day. Dosing was adjusted based on trough sirolimus concentrations to achieve target concentrations between 5 and 10 ng/mL. Sirolimus levels were measured by FDA-approved clinical laboratory assays in Clinical Laboratory Improvement Amendments (CLIA) certified laboratories using liquid chromatography/tandem mass spectrometry to enable accurate and consistent measures of levels across study sites. The average sirolimus level was calculated for each participant using a weighted average to account for the differing numbers of trough measurements. Spearman correlations assessed associations between average trough level and virologic and immunologic changes.

#### Sample size estimation and biological sample allocations

Regarding the assessment of safety, the sample size of 30 sirolimus-treated participants would provide >90% probability of observing a sirolimus-related adverse event that would occur in 8% or more of treated individuals. Statistical power for identifying treatment effects on primary immunologic and virologic endpoints assumed 25 evaluable participants, on changes over 20 weeks of sirolimus treatment. Based on the paired t-test, power was estimated to be 80% to identify an effect size corresponding to a probability of 0.72 that a participant receiving sirolimus would have an observed increase in HIV-1 Gag-specific CD8 responses from pre-to post-treatment (probability 0.5 under the null hypothesis), and similarly a probability of 0.72 to have a decrease in cell-associated RNA. Power was estimated to be 80% to identify sirolimus effects corresponding to iSCA having probability of 0.75 to be below assay limit post-treatment, compared to probability 0.50 pre-treatment.

#### Human samples

Whole blood was collected at timepoints specific in the study protocol (Data S1) followed by plasma separation and isolation of human peripheral blood mononuclear cells (PBMCs). Plasma was frozen and stored at −80°C in a central biospecimen bank until further processing and testing. PBMCs were cryopreserved and stored in liquid nitrogen in a central biospecimen bank until further processing and testing.

#### Study approval

Institutional review boards from each participating ACTG site reviewed and approved the study documents, including informed consent forms. Informed consent was obtained from all participants in the study prior to participation.

### Method details

#### HIV DNA and RNA quantification

HIV reservoir activity and size were assessed by the quantification of cell-associated HIV-1 RNA (CA-RNA) and DNA (CA-DNA).^80^ Intracellular RNA and DNA were isolated from cryopreserved peripheral blood mononuclear cells (PBMCs) using the AllPrep DNA/RNA Mini Kit (Qiagen) as per manufacturer specifications with the addition of the optional spin column drying procedure prior to elusion in nuclease free water. 10 μL of genomic DNA, 12.5 μL of Universal Taqman mastermix (ABI), 0.75 μL of 10 μM forward primer (5′-TACTGACGCTCTCGCACC), 0.75 μL of 10 μM reverse primer (5′-TCTCGACGCAGGACTCG), and 1.0 μL of 5 μM FAM-MGB labeled probe (5′-FAM-CTCTCTCCTTCTAGCCTC) were added to each reaction well. HIV-1 standards were constructed by amplifying a cDNA region with primers that flank the region specified above from the HIV-1 reference strain HXB2 using the forward primer 5′-GGCTCACTATGCTGCCGCCC and the reverse primer 5′-TGACAAGCAGCGGCAGGACC. Cellular integrity for RNA analysis was assessed by the measurement of total extracted RNA and evaluation of the IPO-8 housekeeping gene.^81^ Unspliced CA-RNA and total CA-DNA were quantified using a real-time PCR approach with primers/probes targeting conserved regions of HIV LTR/gag as previously described.^82^ The IPDA was performed as described^41^ at baseline (treatment week 0), treatment 4 and treatment week 20 in participants in the primary efficacy analysis. Primer and probe sequences for the PCR quantitation experiments were as follows: Ψ F (CAGGACTCGGCTTGCTGAAG), Ψ R (GCACCCATCTCTCTCCTTCTAGC), Ψ Probe (TTTTGGCGTACTCACCAGT), Env F (AGTGGTGCAGAGAGAAAAAAGAGC), Env R (GTCTGGCCTGTACCGTCAGC), Env intact probe (CCTTGGGTTCTTGGGA), Env hypermut (CCTTAGGTTCTTAGGAGC). ddPCR was performed on the Bio-Rad QX100 system using the ddPCR Supermix for probes (no dUTPS; Bio-Rad Laboratories). IPDA thermocycling was performed as follows: 1 cycle of enzyme activation at 95C for 10 min, 45 cycles of denaturing (94C)/annealing/extension (59C), 1 cycle of enzyme deactivation (98C) for 10 min and hold for 4-12C until ready for droplet reading. Droplet quantitation was performed using a QX100 ddPCR droplet reader (BioRad).

#### HIV single-copy assay

Plasma HIV residual viremia were measured using the validated ultrasensitive integrase single-copy assay (iSCA),^83^ commonly used to assess levels of residual viral load.^42^^,^^64^^,^^80^^,^^84^^,^^85^^,^^86^^,^^87^ Plasma from the participants was spiked with an internal Replication-Competent ASLV long terminal repeat with a Splice acceptor (RCAS) virion standard as a control for RNA extraction efficiency.^88^ Real-time PCR reactions were performed with a Roche LightCycler 480 system using primers and probes specific to a conserved region of the HIV integrase gene.^83^

#### Plasma inflammation markers

Levels of soluble CD14 (sCD14), IL-6, IL7, and IP10 in EDTA plasma samples were measured using the Quantikine ELISA kits; Cat# DC140, HS600C, HS750 and DIP100 respectively, (all from R&D Systems) according to the manufacturer’s instructions. Levels of D-dimers were measured using the Asserachrom D-DI immunoassay: cat#00947 (Diagnostica Stago, Asnieres France).

#### Flow cytometric analyses

Cryopreserved peripheral blood mononuclear cells (PBMCs) were thawed with RPMI1640 (Gibco) containing 10%FCS (Sigma) and 50U/mL benzonase (EMD Millipore). Cells were stained for 30 min at room temperature with LIVE/DEAD Fixable Aqua Dead Cell Stain Kit (Invitrogen) followed by cell-surface staining with anti-CD3 PerCP (SK7, BioLegend), anti-CD4 AF700 (RPA-T4, BD), anti-CD8 APC-Cy7 (SK1, BioLegend), anti-CCR7 PE-CF594 (150503, BD), anti-CD45RA BB515 (HI100, BD), anti-CD27 BV786 (O323, BioLegend), anti-CCR5 PE (2D7, BD), anti-PD-1 BV421 (EH12.1, BD), and anti-CD69 PE-Cy7 (FN50, BioLegend). Cells were then permeabilized with Foxp3/Transcription Factor Fixation/Permeabilization Concentrate and Diluent (eBioscience) for 30 min at 4°C and stained intracellularly for 45 min at 4°C with anti-Ki67 APC (Ki-67, BioLegend). Samples were acquired on a BD LSRII flow cytometer and analyzed with BD FACSDiva software.

#### *In vitro* stimulations

Cryopreserved PBMCs were thawed with RPMI1640 (Gibco) containing 10%FCS (Sigma) and 50U/mL benzonase (EMD Millipore) and then rested at 37°C in RPMI 1640 with10% FCS, 2mM L-glutamine (Gibco), and 50U/mL Pen Strep (Gibco) for approximately 6 h. Cells were then stimulated with 1 μg/mL HIV-1 Gag peptides, 1 μg/mL SEB (Toxin Technology Inc.), 10 μg/mL human CMV pp65 peptide pool, or a medium control along with Brefeldin A (BioLegend) and CD107α PE-CF594 (H4A3, BD) for 16 h at 37°C. The following reagents were obtained through the NIH AIDS Reagent Program, Division of AIDS, NIAID, NIH: HIV-1 PTE Gag Peptide Set (cat# 11554) and HCMV pp65 Peptide Pool (cat# 11549)^23^^,^^89^^,^^90^

Cells were stained with LIVE/DEAD Fixable Aqua Dead Cell Stain Kit (Invitrogen) followed by cell-surface staining with anti-CD3 BV711 (UCHT1, BD), anti-CD4 BUV395 (SK3, BD), and anti-CD8 APC-Cy7 (SK1, BioLegend). Cells were permeabilized with BD Cytofix/Cytoperm for 30 min at 4°C followed by intracellular staining for 45min at 4°C with anti-IFN-γ PE-Cy7 (B27, BioLegend), anti-TNF-α FITC (Mab11, BioLegend), anti-IL-2 APC (MQ1-17H12, BioLegend), anti-MIP-1β PE (D21-1351, BD), and anti-CD40L/CD154 (24–31, BioLegend). Samples were acquired on a BD LSRII flow cytometer and analyzed with FlowJo software. For each stimulant/marker combination on CD4^+^ and CD8^+^ T cells, there was an analogous unstimulated result measured by the same marker. This value was subtracted from the corresponding HIV GAG-, CMV- and SEB-stimulated result to yield the results used in analysis (setting to zero if subtracting the unstimulated result yielded a negative number).

### Quantification and statistical analysis

#### HIV RNA and DNA

Statistical analyses are based on paired t-tests of each outcome, testing changes from baseline to treatment week 20 for the primary analysis population, and testing changes from baseline to treatment week 4 for the secondary analysis population. Results were log_10_-transformed prior to analyses. For the primary outcome of change in cell-associated HIV-1 RNA, a paired t-test also assessed changes between pre-treatment time points (treatment week −12 (study week 0) and treatment week 0 (study week 12)). Baseline is defined as the average of treatment weeks −12 and 0 (study weeks 0 and 12), or one of these time points if the other is missing. In the figures, both treatment weeks −12 and 0 are presented. To address left-censoring of CA-RNA, the analysis lower limit was determined from the normalized CA-RNA values as the largest assay lower limit across all results; results less than the analysis lower limit were imputed to half the analysis lower limit for analysis. This approach was also implemented for CA-DNA, iSCA and soluble biomarkers of inflammation. A supplemental analysis based on the left-censored normal distribution was also performed, left-censoring at the analysis lower limit.^91^ No adjustments have been made regarding multiple comparisons.

Cell-associated HIV-1 RNA and DNA were normalized to copies/10^6^ CD4^+^ T cells by dividing by CD4% (divided by 100) from the same specimen date as the CA-RNA and CA-DNA sample. In cases where the CD4% was not available on the same date, CD4% from the closest date (before or after) was used. All statistical tests were 2-tailed, and 2-tailed confidence intervals are presented in the graphical results. Linear mixed effects models with random intercept, and fixed effects for the intercept and change from baseline, were applied to pre-treatment and all 3 on-treatment time points (treatment wk4, wk12, wk20), and combining the primary and secondary analysis populations (i.e., *n* = 29 contributing to pre-treatment and wk4, *n* = 16 contributing to wk12 and wk20) to further explore the relationship between sirolimus use and HIV-1 persistence measures over time.

#### IPDA

To summarize IPDA changes (separately for changes to treatment week 4 and to treatment week 20), a descriptive approach was performed given the smaller evaluable sample size, excluding participants with no IPDA change (IPDA not detected at both time points). Longitudinal changes were estimated by the median change, derived from participant-specific changes of log_10_-transformed measures, and the proportion with decreases vs. increases. Participants whose intact proviral DNA levels went from detected to not detected were analyzed as the change with the lowest rank (greatest decreases). Participants whose intact proviral DNA levels went from not detected to detected were analyzed as the change with the highest rank (greatest increases).

All statistical and analysis codes used in analyses will be provided upon request. Data acquisition of clinical data at the sites was obtained prospectively during the study using the study case report form (CRFs), with data entry at each participating clinical site using standard ACTG procedures and trained personnel. Batch-tested laboratory data was generated at each testing lab and submitted securely (using Excel templates) to the ACTG’s data management center, where it was loaded into INGRES tables after quality assurance review. All study data was then transferred securely to the statistical analysis center and converted to SAS datasets using validated programs. Statistical analyses were performed using SAS version 9.4. A supplemental analysis was performed in R using the lmec package.

#### Flow cytometric analyses

Longitudinal summaries are presented. Statistical analyses are based on 2-tailed paired t-tests for the secondary flow-based outcomes, testing changes from baseline to treatment week 20 for the primary analysis population, and testing changes from baseline to treatment week 4 for the secondary analysis population.

### Additional resources

ClinicalTrial.Gov registration number: NCT02440789 (https://clinicaltrials.gov/study/NCT02440789).

## Supplemental information

Document S1. Figures S1 and S2, Tables S1–S4, and Data S1

Document S2. Article plus supplemental information
