# Supplementary material for: Sirolimus reduces T cell cycling, immune checkpoint marker expression, and HIV-1 DNA in people with HIV
Source: Cell Rep Med. 2024 Sep 24;5(10):101745. doi: 10.1016/j.xcrm.2024.101745 (PMC11513808; doi:10.1016/j.xcrm.2024.101745)
Supplement: Document S2. Article plus supplemental information [file mmc2.pdf]

Cell Reports
Medicine

Article

Sirolimus reduces T cell cycling, immune checkpoint marker expression, and HIV-1 DNA in people with HIV

Graphical abstract

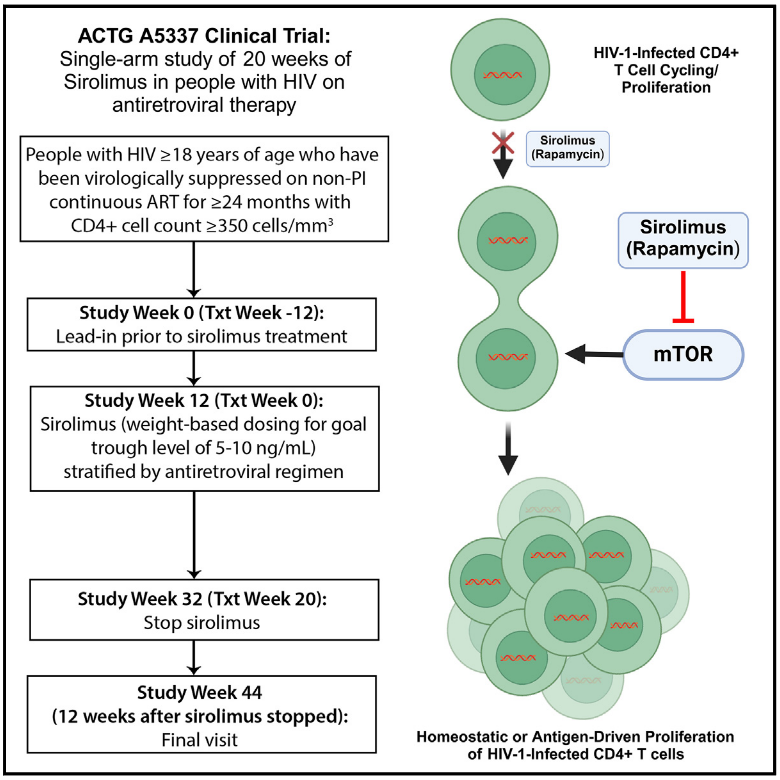

Highlights

- Sirolimus treatment decreases cell-associated HIV-1 levels in people with HIV on ART
- The reduction in HIV DNA persists for at least 12 weeks following sirolimus treatment
- Sirolimus reduces CD4$^+$ T cell cycling and CD8$^+$ T cell PD-1 expression
- Sirolimus does not impact HIV-1-specific CD8 T cell responses or HIV-1 RNA levels

Authors

Timothy J. Henrich, Ronald J. Bosch, Catherine Godfrey, ..., Priscilla Y. Hsue, Steven G. Deeks, the ACTG A5337 Team

Correspondence

timothy.henrich@ucsf.edu

In brief

Henrich et al. show that sirolimus (rapamycin) reduces HIV-1 proviral DNA, perhaps through decreased cell cycling that limits the homeostatic proliferation of infected CD4$^+$ T cells. Conceptually, these data suggest that homeostatic proliferation of infected cells, an important mechanism for HIV persistence, might be targeted therapeutically.

Henrich et al., 2024, Cell Reports Medicine *5*, 101745
October 15, 2024 © 2024 The Author(s). Published by Elsevier Inc.
https://doi.org/10.1016/j.xcrm.2024.101745

CellPress

Cell Reports Medicine

CellPress
OPEN ACCESS

Article

Sirolimus reduces T cell cycling, immune checkpoint marker expression, and HIV-1 DNA in people with HIV

Timothy J. Henrich,[1,11,*] Ronald J. Bosch,[2] Catherine Godfrey,[3] Hanna Mar,[2] Apsara Nair,[4] Michael Keefer,[5] Carl Fichtenbaum,[6] Daniela Moisi,[7] Brian Clagett,[7] Amanda M. Buck,[1,8] Amelia N. Deitchman,[9] Francesca Aweeka,[9] Jonathan Z. Li,[10] Daniel R. Kuritzkes,[10] Michael M. Lederman,[7] Priscilla Y. Hsue,[1] Steven G. Deeks,[1] and the ACTG A5337 Team

[1]Department of Medicine, University of California San Francisco, San Francisco, CA 94110, USA
[2]Center for Biostatistics in AIDS Research, Harvard T.H. Chan School of Public Health, Boston, MA 02115, USA
[3]Division of AIDS, National Institute of Allergy and Infectious Diseases, Bethesda, MD 20892, USA
[4]Frontier Science and Technology Research Foundation, Amherst, NY 14226, USA
[5]Department of Medicine, University of Rochester School of Medicine, Rochester, NY 14642, USA
[6]Department of Medicine, University of Cincinnati College of Medicine, Cincinnati, OH 45267, USA
[7]Department of Medicine, Case Western Reserve University School of Medicine, Cleveland, OH 44106, USA
[8]San Francisco State University, San Francisco, CA 94132, USA
[9]Department of Clinical Pharmacology, University of California San Francisco, San Francisco, CA 94110, USA
[10]Division of Infectious Diseases, Brigham and Women's Hospital, Harvard Medical School, Boston, MA 02115, USA
[11]Lead contact
*Correspondence: timothy.henrich@ucsf.edu
https://doi.org/10.1016/j.xcrm.2024.101745

SUMMARY

Key HIV cure strategies involve reversing immune dysfunction and limiting the proliferation of infected T cells. We evaluate the safety of sirolimus, a mammalian target of rapamycin (mTOR) inhibitor, in people with HIV (PWH) and study the impact of sirolimus on HIV-1 reservoir size and HIV-1-specific immunity in a single-arm study of 20 weeks of treatment in PWH on antiretroviral therapy (ART). Sirolimus treatment does not impact HIV-1-specific CD8 T cell responses but leads to a significant decrease in $CD4^+$ T cell-associated HIV-1 DNA levels at 20 weeks of therapy in the primary efficacy population (n = 16; 31% decline, p = 0.008). This decline persists for at least 12 weeks following cessation of the study drug. Sirolimus treatment also leads to a significant reduction in $CD4^+$ T cell cycling and PD-1 expression on $CD8^+$ lymphocytes. These data suggest that homeostatic proliferation of infected cells, an important mechanism for HIV persistence, is an intriguing therapeutic target.

INTRODUCTION

Despite sustained inhibition of virus replication by antiretroviral therapy (ART), HIV persists indefinitely. Chronic viral-associated inflammation and immune dysfunction also persist in most people with HIV (PWH). Substantial evidence supports the concept that the chronic inflammatory environment associated with treated HIV disease results in a dysfunctional virus-specific immune response and inability of the host immune response to clear the persistent viral reservoir.[1–3] Furthermore, the proliferation of infected $CD4^+$ T cells leads to the indefinite persistence of HIV reservoirs that escape immune targeting and clearance.[4] To date, few prospective immunotherapy trials, including those that target cell proliferation and immune modulation, have demonstrated significant decreases in HIV-1 DNA burden *in vivo*, particularly in those on long-term, stable ART.

The mammalian target of rapamycin (mTOR) is a regulatory kinase that controls cell cycle progression.[5–10] Despite its immunosuppressive consequences, pharmacologic mTOR inhibition leads to changes in several immune regulatory pathways that may enhance antiviral activity and limit $CD4^+$ T cell homeostatic proliferation and cell cycling.[11–27] As homeostatic proliferation of infected $CD4^+$ T cells is a key mechanism for the maintenance of the latent HIV reservoir,[28–35] targeting the heightened and dysregulated $CD4^+$ T cell cycling is a plausible strategy to limit reservoir persistence.[4] Sirolimus (rapamycin) is a selective TORC1 inhibitor with suppressive effects on cell cycle progression that predominately targets lymphocytes activated by cytokines rather than by antigen-T cell receptor engagement.[36,37] In treated and untreated HIV infection, increased $CD4^+$ and $CD8^+$ T cell cycling and proliferation appear to be bystander effects largely driven by cytokine exposure.[38–40] Prior cross-sectional data suggest that the use of sirolimus in ART-suppressed renal transplant recipients with HIV may be associated with lower peripheral total $CD4^+$ T cell HIV-1 DNA levels.[14] Prospective trials designed to investigate the impact of mTOR inhibition on HIV

© 2024 The Author(s). Published by Elsevier Inc.
This is an open access article under the CC BY license (http://creativecommons.org/licenses/by/4.0/).

persistence and the mechanisms by which reservoir reduction may be achieved are lacking.

We therefore conducted a phase 1/2, single-arm clinical trial to evaluate the effect of 20 weeks of continuous oral sirolimus use on HIV-specific immune responses, residual HIV-1 transcriptional activity, viral reservoir size, and immune phenotypes in PWH receiving long-term antiretroviral therapy (Advancing Clinical Therapeutics Globally [ACTG] protocol A5337; NCT02440789). Given that the main objective of this study was to assess the biological activity of the study drug, the protocol-defined, pre-specified primary analyses were based on the as-treated population, limited to participants who completed the full 20 weeks of study drug.

RESULTS

Study overview and enrollment

Eligible persons were on ART for at least 24 months with CD4^{+} T cell counts $\geq$350 cells/uL and no history of systemic malignancy or recent immunomodulator use. Participants were followed for 12 weeks prior to initiating sirolimus to establish baseline levels of HIV reservoir and immunological stability. Participants then received oral sirolimus, dosed to achieve a trough plasma level of 5–10 ng/mL, which is within the range for the prevention of solid organ transplant rejection and prophylaxis against graft-versus-host disease following allogeneic stem cell transplantation (Figure 1). After treatment week 20, sirolimus was discontinued, and participants remained in the study for an additional 12 weeks to measure the longer-term impact of mTOR inhibition on HIV burden, immune responses, and inflammation.

The primary endpoints were: (1) safety, (2) changes in the frequency (%) of HIV-1 Gag-specific CD8^{+} cells after 20 weeks of sirolimus therapy, and (3) changes in CD4^{+} T cell-associated unspliced HIV-1 RNA and plasma HIV-1 RNA by the integrase single-copy assay (iSCA) from baseline to week 20 of sirolimus therapy. Secondary endpoints included changes in total cell-associated HIV-1 DNA levels, HIV-1-specific CD4^{+} T cell responses, and markers of T cell activation and cycling. As pre-defined in the study protocol, primary analyses involved the as-treated population, given that this early-phase study was conducted to assess the biological activity of mTOR inhibition in PWH on ART. Baseline participant characteristics, including demographics, ART regimen, baseline CD4^{+} T cell counts, weight, and plasma HIV-1 RNA measurements are listed in Table 1. Overall, 28% of enrolled participants were female and 72% were non-White. The median age was 52 and median CD4^{+} T cell count was 813 cells/μL. Forty-four percent of participants were on an integrase inhibitor-based regimen with the remainder on a non-nucleoside reverse transcriptase inhibitor (NNRTI)-based regimen. No participants were on protease inhibitor-based regimens as per protocol, given the significant pharmacological interactions with sirolimus.

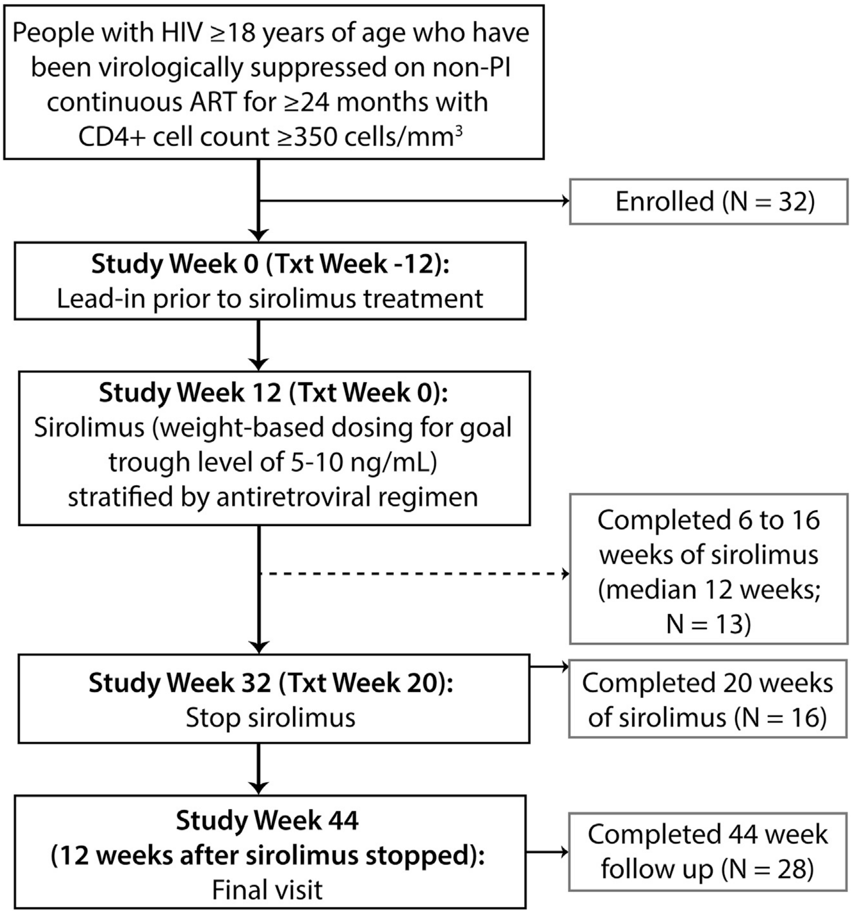

Figure 1. ACTG A5337 study schema and enrollment
Study and treatment weeks are shown with the number of participants enrolled and completing trial milestones. Sixteen participants completed the full 20 weeks of planned oral sirolimus and are defined as the primary analysis population, and 13 participants completed 6 to 16 weeks of sirolimus (median 12 weeks) and are defined as the secondary analysis population. Twenty-nine participants completed at least 6 weeks of study drug and comprise the secondary analysis population.

Sirolimus therapy in participants with HIV on ART

A total of 32 participants across 10 sites were enrolled into A5337 between December 2015 and March 2017. Twenty-eight participants completed the full 44 weeks of study follow-up (Figure 1). Two participants withdrew from the study prior to the initiation of sirolimus, and two discontinued study 2 and 16 weeks after starting the drug (not able to attend clinic and withdrawal of consent, respectively). Sixteen participants completed the full 20 weeks of

CellPress
OPEN ACCESS

Table 1. Entry and baseline characteristics of study populations

Characteristic	All enrolled (*N* = 32)	Included in safety analysis (*n* = 30)	Primary efficacy population[a] (*n* = 16)	Secondary efficacy population[b] (*n* = 13)
Age at study entry				
Median (Q1, Q3)	52 (46, 55)	52 (46, 55)	52 (43, 59)	52 (46, 53)
Female gender				
N (%)	9 (28%)	7 (23%)	3 (19%)	3 (23%)
Race/Ethnicity				
White Non-Hispanic	9 (28%)	8 (27%)	6 (38%)	2 (15%)
Black Non-Hispanic	18 (56%)	18 (60%)	7 (44%)	10 (77%)
Hispanic	4 (13%)	3 (10%)	2 (13%)	1 (8%)
More than one race	1 (3%)	1 (3%)	1 (6%)	0 (0%)
IV drug history				
Never used	29 (91%)	27 (90%)	14 (88%)	12 (92%)
Previously	3 (9%)	3 (10%)	2 (12%)	1 (8%)
Entry HIV plasma RNA				
≥40 copies/mL	0 (0%)	0 (0%)	0 (0%)	13 (100%)
Txt week 0 HIV plasma RNA				
≥40 copies/mL	1 (3%)	1 (3%)	0 (0%)	1 (8%)
Baseline $CD4^+$ T cell count (c/mm^3)				
Median (Q1, Q3)	813 (618, 1,014)	818 (635, 1,031)	846 (662, 1,065)	765 (635, 972)
Baseline CD4/$CD8^+$ ratio				
Median (Q1, Q3)	0.9 (0.7, 1.3)	0.9 (0.7, 1.3)	0.9 (0.7, 1.4)	1.0 (0.8, 1.3)
Baseline weight (kg)				
Median (Q1, Q3)	83 (68, 99)	83 (68, 99)	79 (64, 93)	90 (77, 100)
Baseline BMI (kg/m^2)				
Median (Q1, Q3)	27 (23, 31)	27 (23, 30)	24 (22, 29)	29 (26, 31)
Entry ARV regimen				
Non-PI, non-NNRTI (INSTI) based	14 (44%)	13 (43%)	9 (56%)	4 (31%)
Non-PI, RPV based	3 (9%)	2 (7%)	1 (6%)	1 (8%)
Non-PI, other NNRTI based	15 (47%)	15 (50%)	6 (38%)	8 (62%)[c]

IV, intravenous; BMI, body mass index; ARV, antiretroviral; PI, protease inhibitor; NNRTI, non-nucleoside reverse transcriptase inhibitor; INSTI, integrase strand transfer inhibitor; RPV, rilpivirine. Baseline is the average of pre-sirolimus measurements at study weeks 0 and 12.
[a]Includes participants who completed the full 20 weeks of sirolimus.
[b]Includes participants who completed 6 to 16 weeks of sirolimus.
[c]Participants in the primary efficacy population had lower efavirenz (NNRTI) use than in secondary efficacy population (38% versus 62%).

sirolimus and comprised the primary efficacy analysis population. An additional thirteen participants completed 6 to 16 weeks of sirolimus therapy (median 12 weeks) and were grouped into a secondary efficacy analysis population. Overall, participants received a median of 19.1 weeks of sirolimus (1.0–21.6 weeks). In contrast to all enrolled participants, 56% of the primary efficacy analysis population was on non-NNRTI-based antiretroviral regimen and there was a greater percentage of White, non-Hispanic participants (38%) (Table 1). Participants in the secondary efficacy analysis population (i.e., those unable to complete the full course of therapy) had a higher weight and BMI, lower absolute $CD4^+$ T cell counts, and a higher rate of efavirenz use as shown in Table 1.

Safety analyses

Thirty participants received at least one dose of sirolimus and comprised the study-defined primary safety analysis population. No participants experienced protocol-defined virologic failure (confirmed plasma HIV-1 RNA ≥200 copies/mL). Of the 30 participants who initiated sirolimus, 20 had an adverse event judged related to sirolimus (the maximum grades were 1, 2, and 3 for 4, 13, and 2 individuals, respectively; one had a non-graded non-fasting triglyceride event; 12 of these 20 completed the full 20 weeks of sirolimus). Lower grade toxicities that did not lead to treatment discontinuation were predominantly related to increases in fasting blood glucose levels or abnormalities in the fasting lipid panel (*e.g.*, increased triglycerides). These laboratory abnormalities are known effects of sirolimus.[8,9]

Twenty participants had a treatment-related safety event, but only two were graded as severe. Fourteen participants prematurely discontinued study treatment (Table S1). Whereas the discontinuation rate was higher than expected, a majority of participants who stopped therapy did so for reasons other than

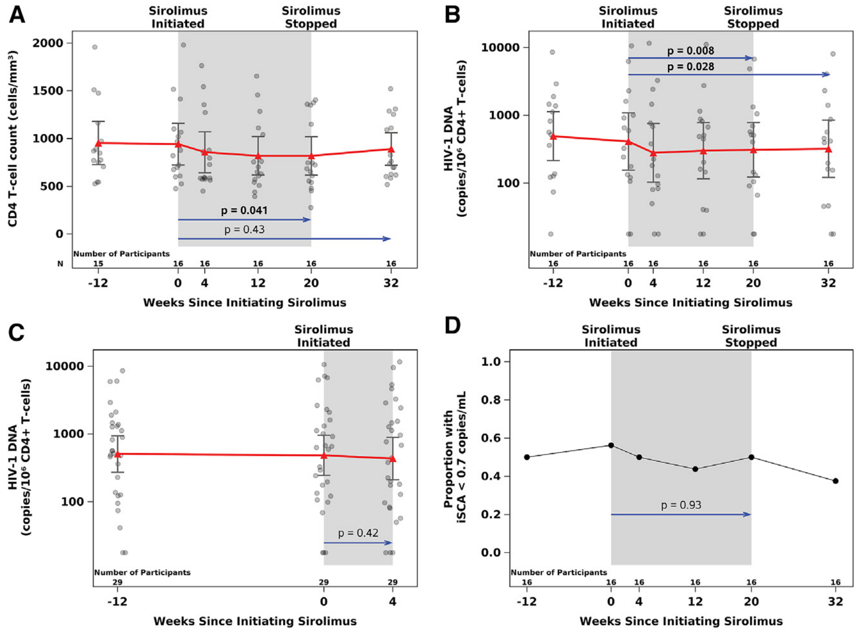

Figure 2. Changes in CD4⁺ T cell counts, CD4⁺ T cell-associated HIV-1 DNA, and low-level residual plasma HIV-1 RNA in response to sirolimus treatment

Transient decreases in CD4⁺ T cell counts (A) and sustained and significant reduction in CD4⁺ T cell-associated HIV-1 DNA (B) in the primary analysis population are shown. CD4⁺ T cell-associated HIV-1 DNA at baseline and week 4 time points in the secondary analysis population is shown in (C). HIV-1 DNA levels remain significantly decreased 12 weeks after discontinuation of sirolimus in the primary efficacy population. The proportion of participants with undetectable low-level residual HIV-1 plasma RNA measured by iSCA for the primary analysis population is shown in (D). 95% confidence intervals and means at each time point are presented. *p* values from two-tailed, paired t tests. Baseline values were defined as the average of measures from treatment weeks −12 and 0 in statistical analyses.

protocol-defined adverse events (e.g., personal choice or on the recommendation of their medical provider). Three participants met the pre-specified primary safety outcome. Two participants experienced a grade 3 adverse event related to sirolimus (stomatitis and elevated fasting glucose), and one had a confirmed CD4⁺ T cell count decrease to below 300 cells/μL.

Pharmacokinetic measurements and dosing

Oral sirolimus use requires frequent monitoring in order to establish and maintain trough levels within the desired range. Throughout the study, the median within-participant average trough sirolimus level was 6.8 ng/mL (range 5.4–7.8) over a median 11 measurements for each participant in the primary efficacy analysis population. The therapeutic goal was 5–10 ng/mL for sirolimus trough levels during the first 4 weeks of therapy as participants were achieving steady-state concentrations for the primary and secondary analysis populations. Interestingly, participants in the primary efficacy population had higher time-averaged trough levels than in the secondary analysis population (6 ng/mL versus 5.1 ng/mL).

Impact of sirolimus on CD4+T cell counts

A modest and transient decrease in CD4⁺ T cell counts was observed in the safety analysis population (n = 30; median decline of 37–91 cells/μL at treatment weeks 2–20 relative to a median baseline CD4⁺ T cell count of 818 cells/μL). In the primary efficacy population (n = 16 who completed 20 weeks of oral sirolimus), there was a significant decline in CD4⁺ (mean: −118 cells/μL; p = 0.041) and CD8⁺ (mean: −160 cells/μL; p = 0.021) T cell counts between baseline and treatment week 20 by paired t tests, but no change in CD4/CD8 ratios. In the combined primary and secondary efficacy populations, CD4⁺ T cell counts decreased by a mean of 60 cells/μL (p = 0.06) from baseline to treatment week 4 (n = 29). CD4⁺ T cell counts in the primary efficacy population decreased from baseline to treatment week 4 (−79 cell cells/μL), and CD4⁺ T cell counts declined by an average of 37 cells/μL from baseline to treatment week 4 in the secondary analysis population (n = 13). However, CD4⁺ T cell counts increased 12 weeks following cessation of sirolimus and were not significantly different at the post-treatment time point from baseline values in either population as shown in Figure 2A.

Antigen-specific T cell responses

To test the effect of sirolimus treatment on viral antigen-specific T cell responses, flow cytometric evaluation of intracellular cytokine responses (IFNγ, IL-2, TNF-α, and MIP-1β) and cell surface markers (CD40L and CD107a) following a 6 h stimulation of peripheral blood mononuclear cells in the presence or absence of overlapping HIV-1 Gag peptide pools, CMV (pp65) antigen, and Staphylococcal enterotoxin B (SEB; positive stimulation control) was performed as shown in Figure S1. Overall, no significant changes were observed in the frequency of Gag-specific CD8⁺ or CD4⁺ T cells expressing any marker from baseline to treatment week 20 in the primary efficacy analysis population or between baseline and week 4 in the secondary efficacy population.

Impact of sirolimus on cell-associated HIV-1 DNA and RNA

Cell-associated (CA) DNA and RNA were measured at baseline, treatment weeks 4, 12, and 20, and 12 weeks after treatment was discontinued (week 32) (Figure 2B; Table S3). Although CD4⁺ T cell-associated unspliced HIV-1 RNA levels decreased from baseline to week 20 in the primary efficacy population, this change did not reach statistical significance (mean: −0.21 $\log_{10}$ copies/10^6 cells, p = 0.11). In the primary efficacy population (n = 16), there was a significant decrease in CD4⁺ T cell-associated HIV-1 DNA from baseline to treatment week 20 (mean DNA decline of 0.16 $\log_{10}$ copies/10^6 cells, p = 0.008), which corresponds to a 31% decline (Figure 2B). The lower levels of CA-DNA persisted after sirolimus discontinuation

Table 2. Change in percentage of T cell markers and subsets from baseline to sirolimus treatment week 20 in the primary efficacy population ($N = 16$)

	CD8$^+$ T cells		CD4$^+$ T cells	
	Mean (CI)	p	Mean (CI)	p
% PD-1+	−2.85 (−4.85, −0.86)	0.008[a]	0.42 (−1.81, 2.66)	0.69
% Ki67+	−0.54 (−0.90, −0.19)	0.005	−0.51 (−0.97, −0.05)	0.031
% CCR5+	−3.92 (−5.99, −1.85)	0.001	−1.67 (−3.76, 0.42)	0.11
% CD69$^+$	−0.52 (−1.38, 0.35)	0.22	−0.17 (−0.97, 0.63)	0.65
% $T_{Naïve}$	0.75 (−3.61, 5.12)	0.72	−2.16 (−5.46, 1.15)	0.18
% T_{CM}	−0.87 (−1.82, 0.08)	0.07	−0.01 (−2.18, 2.17)	1.00
% T_{EM}	−0.58 (−3.02, 1.86)	0.62	2.20 (−0.50, 4.90)	0.10
% T_{TD}	0.68 (−2.82, 4.18)	0.69	−0.04 (−1.21, 1.14)	0.95

T_{CM}, central memory; T_{EM}, effector memory; T_{TD}, terminally differentiated.
[a]From two-tailed, paired t tests.

resulting in a persistent decrease in CD4$^+$ T cell-associated HIV-1 DNA (mean −0.15 $\log_{10}$ copies/10^6 cells; p = 0.028; primary efficacy population) from baseline to week 32 (12 weeks following cessation of sirolimus). Overall, the magnitude of mean change in CD4$^+$ T cell-associated HIV-1 DNA was similar between baseline and all treatment time points with a borderline significant mean change in HIV-1 DNA levels from baseline to treatment week 4 being observed in the primary efficacy population (mean −0.21 $\log_{10}$ copies/10^6 cells; p = 0.05) but not from baseline to week 12 (mean: −0.17 $\log_{10}$ copies/10^6 cells; p = 0.13) as shown in Table S3. There were no significant changes in CA-DNA from baseline to treatment week 4 in the secondary efficacy population as shown in Figure 2C.

To further understand the impact of sirolimus on total HIV-1 DNA levels, a linear mixed-effects model was applied to all on-treatment time points (weeks 4, 12, 20) together with the pre-treatment data, to estimate change from baseline, combining the primary and secondary analysis populations (n = 29 contributing to the treatment week 4 time point and n = 16 contributing to the weeks 12 and 20 time points); the estimated $\log_{10}$ change in CD4$^+$ T cell-associated HIV-1 DNA was −0.08 (p = 0.22). Applying this model restricted to the primary analysis population (n = 16), the estimated $\log_{10}$ change was −0.18 (p = 0.026).

Impact of sirolimus on residual low-level plasma HIV-1 RNA

Low-level plasma HIV-1 RNA was measured using iSCA, which had a lower limit of detection of 0.7 copies/mL. Among the primary efficacy population, 46 of 96 (48%) total test results were below this limit. There were no apparent changes in the frequency of detectable plasma RNA as shown in Figure 2D, and similarly, no evidence of a change in the frequency of detectable plasma RNA up to treatment week 4 in the secondary efficacy analysis population. Due to a high percentage of iSCA results less than the analysis lower limit, a supplemental analysis accounting for left-censoring was performed for the change in iSCA from baseline to treatment week 20 (primary efficacy population) and from baseline to treatment week 4 (secondary efficacy population). Results were consistent with those from the paired t tests as aforementioned, with no evidence of changes in iSCA levels after sirolimus treatment.

Impact of sirolimus on intact proviral DNA

The intact proviral DNA assay (IPDA) was performed as described[41] at baseline (treatment week 0), treatment week 4, and treatment week 20 in participants in the primary efficacy population (n = 16). Many of these participants had no detectable intact proviral DNA at treatment week 0 (56%), treatment week 4 (47%), and treatment week 20 (69%, the primary treatment endpoint). Participants who had detectable intact proviral DNA at any time point were included in a descriptive analysis of the change in the $\log_{10}$-transformed intact proviral DNA copies/10^6 CD4$^+$ T cells as previously reported for analyses using the IPDA.[41] At treatment week 4, 67% (6 of 9) of evaluated participants had decreased intact proviral DNA (median change of −0.14 $\log_{10}$, n = 9), and 75% (6 of 8) of evaluated participants had decreased intact proviral DNA (median change of −0.40 $\log_{10}$, n = 8) at treatment week 20.

Markers of T cell exhaustion, memory, activation, and cycling

Changes in markers of T cell activation (CD69), exhaustion (PD-1), naive and memory cell phenotypes (naive, central memory, effector memory, terminally differentiated), cycling/proliferation (Ki67), and CCR5 expression between baseline and week 20 for the primary efficacy population are shown in Table 2. The frequency of CD4$^+$ and CD8$^+$ T cells expressing Ki67+ and CD8$^+$ T cells expressing CCR5 and PD-1 significantly decreased between baseline and week 20 in the primary efficacy population (all $p \leq 0.031$; Table S2). Of note, the significant reduction in the frequency of Ki67-expressing T cells was no longer observed 12 weeks after discontinuation of sirolimus. In analyses of T cell phenotyping in the secondary efficacy population, a significant reduction in the frequency of the naive CD4$^+$ T cells between baseline and week 4 (p = 0.015) was also observed.

Soluble marker of inflammation and immune activation

To evaluate the longitudinal impact of sirolimus treatment on inflammation, we quantified changes in plasma IL-6, IL-7, IP-10, sCD14, and D-dimer using marker-specific ELISA at sirolimus treatment weeks −12, 0, 4, 12, 20, and 32 (12 weeks following cessation of therapy) as shown in Figure 3. Pro-inflammatory markers IL-6 (mean change: 0.31 $\log_{10}$; p = 0.003),

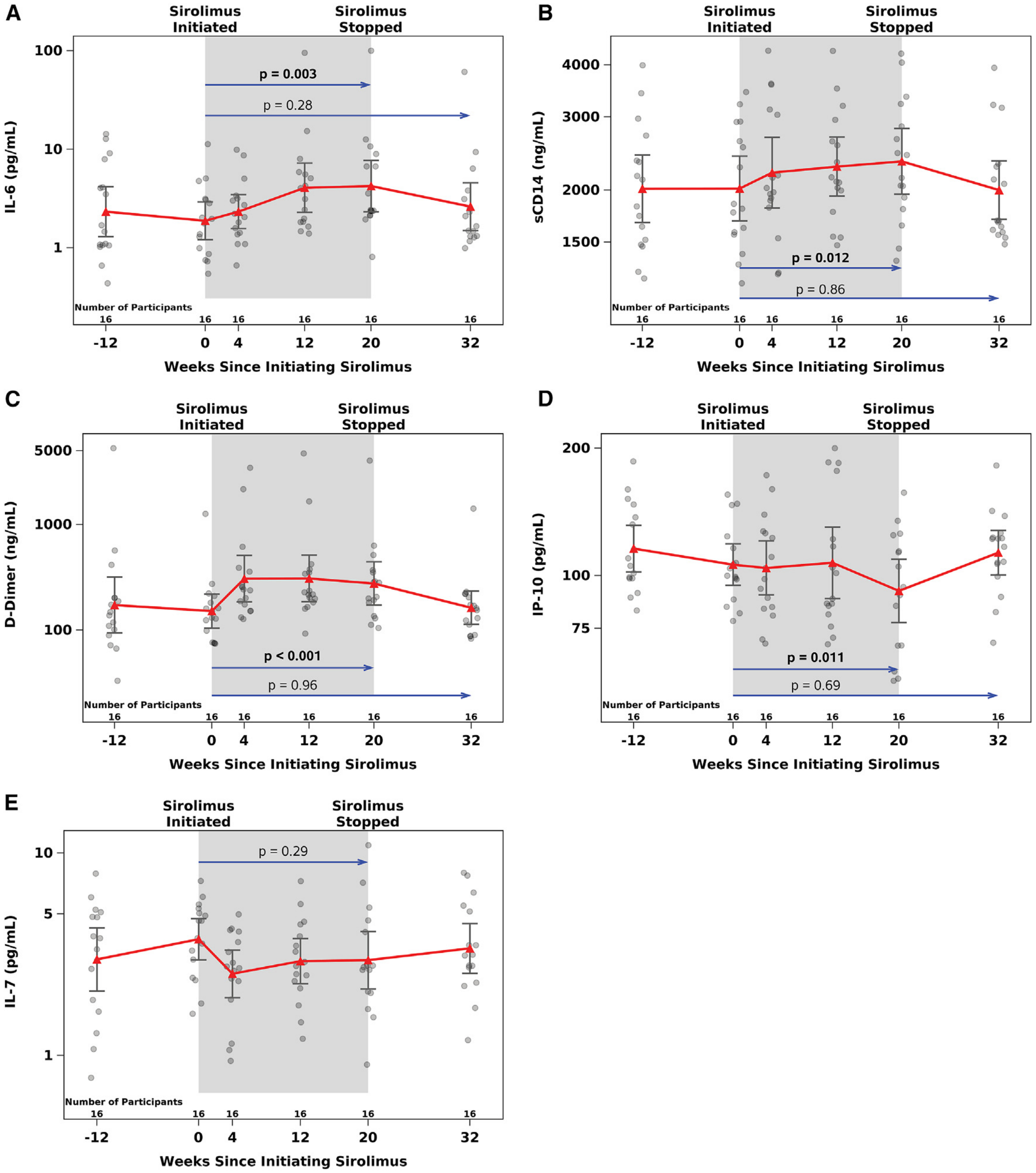

Figure 3. Changes in soluble markers of inflammation in response to sirolimus treatment

Significant increases in circulating IL-6, sCD14, and D-Dimer are shown in (A)–(C). Levels returned to baseline 12 weeks after the cessation of treatment. A significant decrease in interferon gamma-induced protein 10 (IP-10) levels was observed at treatment week 20, which return to baseline 12 weeks after cessation of sirolimus therapy (D). IL-7 levels for the primarily analysis population are shown in (E). 95% confidence intervals and geometric means at each time point are presented. *p* values are from two-tailed, paired t tests. Baseline values were defined as the average of measures from treatment weeks −12 and 0 in statistical analyses.

sCD14 (mean change: 0.07 $\log_{10}$; $p = 0.012$), and D-Dimer (mean change: 0.23 $\log_{10}$; $p = <0.001$) increased significantly from baseline to treatment week 20, whereas IP-10 significantly decreased (mean change: −0.08 $\log_{10}$; $p = 0.01$) from baseline to treatment week 20 in the primary efficacy analysis population. In the secondary efficacy population, sCD14 (mean change: 0.05 $\log_{10}$; $p = 0.002$) and D-Dimer (mean change: 0.25 $\log_{10}$; $p < 0.001$) increased from baseline to treatment week 4. However, no change was evident in the soluble biomarkers of inflammation from baseline to treatment week 32, 12 weeks following cessation of sirolimus. As expected, with the normalization of these markers after discontinuation of sirolimus, all showed

evidence of a significant change between treatment week 20 and 12 weeks following cessation of the drug in the opposite direction of those observed from baseline to treatment week 20 as shown in Figure 3.

Correlation between sirolimus drug levels, immune phenotyping, markers of inflammation, and outcome measures

Overall, there were no significant correlations between sirolimus trough levels and changes to treatment week 20 in CA-RNA or DNA levels, soluble markers of inflammation, or percentages of $CD4^+$ and $CD8^+$ T cells expressing Ki67, CCR5, or PD-1. However, a positive correlation between sirolimus levels and changes in $CD4^+$ T cell counts (Spearman r = 0.55; p = 0.027) was observed. There were also no significant correlations between the change in percentage of total $CD4^+$ T cells, $CD4^+$ or $CD8^+$ T cells expressing Ki67, and $CD8^+$ T cells expressing PD-1 with changes in HIV-1 DNA, CA-RNA, or total $CD4^+$ T cell counts from baseline to treatment week 20 in the primary efficacy analyses. Similarly, there were no significant associations between changes in circulating levels of IL-6, sCD14, IP-10, and D-Dimer with changes in HIV-1 DNA, CA-RNA, or total $CD4^+$ T cell counts in the primary efficacy population (Table S4).

DISCUSSION

Despite years of research to achieve long-term HIV remission, few interventions have reduced cell-associated HIV-1 DNA levels in PWH who are receiving effective ART. Whereas allogeneic stem cell transplantation and initiation of ART during "hyperacute" infection are associated with very low reservoir sizes,[42,43] these strategies are not applicable to the vast majority of people living with HIV. Based on data from multiple groups that memory T cell clonal proliferation is a major mechanism for HIV persistence during long-term ART,[28–35] we postulated that inhibition of proliferation might result in a reduction in reservoir size, a hypothesis predicted by recent modeling.[4] Here, we tested the hypothesis that inhibition of mTOR would reduce the size of the reservoir, as estimated by $CD4^+$ T cell-associated HIV-1 DNA, and other measures. We chose to study mTOR inhibitors based on prior hypothesis-generating studies performed by our group,[14,44] and based on the observations that mTOR inhibition might reduce cytokine-driven homeostatic proliferation.[45,46] Although the sample size was small and the study was complicated by a larger-than-expected frequency of premature treatment discontinuations, we found evidence that sirolimus treatment was associated with a modest reduction in cell-associated DNA levels persisting up to 12 weeks after cessation of the drug. We also found that mTOR inhibition reduced cell cycling (as defined by Ki67) in $CD4^+$ and $CD8^+$ T cells, reduced CCR5 expression on $CD8^+$ T cells, and may have reduced T cell exhaustion (as evidenced by reduced PD-1 expression on effector cells).

Most of the HIV proviruses are defective.[47] As both defective and intact genomes are maintained by homeostatic proliferation, the effect of non-specifically inhibiting proliferation of all memory cells should result in similar reductions of defective and intact genomes. Still, given that the intact and potentially replication-competent reservoir is the main focus of most cure interventions, we estimated the size of the intact reservoir before and after sirolimus using the IPDA. Most participants had no readily detectable intact genomes, due in part to the limited number of cells available. Despite these limitations, we observed a median 0.40 $\log_{10}$ decrease in intact HIV DNA over the 20-week treatment time course in those with detectable levels at any visit. A prior study of the impact of ART alone on intact proviral DNA (ACTG A5321) showed a median half-life of 7.1 years, which corresponds to a -0.016 $\log_{10}$ change over 20 weeks.[48] Results from A5321 also demonstrate that change in total DNA on ART has a much slower rate of decline (half-life of 41.6 years) highlighting the importance of the observed reductions in total HIV DNA on sirolimus,[48] and that the decay in intact proviral DNA on study drug may be an order of magnitude greater than natural decay of the reservoir on ART.

These data support preliminary observations from a prior retrospective cross-sectional study of solid organ transplant recipients, in which persons treated with sirolimus had an approximately 0.3 $\log_{10}$ copies/mL lower level of HIV-1 DNA than did persons receiving other immune-modulating drugs (e.g., cyclosporine and tacrolimus).[14] This modest effect is consistent with the effect observed in our longitudinal study and larger than an expected $\sim$2% decline over 20 weeks observed on stable ART.[49] We also observed in a recent pilot study of 10 PWH on ART, who switched to or added everolimus (an mTOR inhibitor with broad TORC1/TORC2 activity) for graft rejection following solid organ transplantation, that it did not have an overall effect on cell-associated HIV-1 DNA and RNA levels. However, participants who maintained everolimus time-averaged trough levels >5 ng/mL during the first 2 months of therapy had significantly lower $CD4^+$ T cell HIV RNA levels up to 6 months after the cessation of study drug.[44] Time-averaged everolimus trough levels significantly correlated with greater inhibition of mTOR gene pathway transcriptional activity. In this study, there was an absence of a clear pharmacodynamic relationship between sirolimus exposure and outcome measures, but there was limited variation in drug exposure between participants as intensive monitoring and adjustment of sirolimus levels were incorporated.

The sustained reduction in $CD4^+$ T cell-associated HIV-1 DNA levels was limited to our primary analysis population who completed all 20 weeks of daily sirolimus treatment. Although we observed a modest decrease in cell-associated HIV-1 DNA in the remaining 13 participants who completed 6–16 weeks of sirolimus, these results were not statistically significant. Interestingly, these participants tended to have higher weight and BMI, lower $CD4^+$ and $CD8^+$ T cell counts, and higher rates of efavirenz use at baseline. Importantly, these participants also had lower time-averaged sirolimus trough levels while on study drug, perhaps related to challenges with achieving stable trough drug levels with weight-based dosing in those with higher BMI, which may have subsequently played a role in HIV-1 DNA response differences from those that completed the 20 planned weeks of treatment.

Several factors may have led to the observed sustained reductions in peripheral blood $CD4^+$ T cell-associated HIV-1 DNA levels in the primary analysis population. Firstly, Ki67, an intracellular marker of T cell cycling, was significantly reduced during the

period of oral sirolimus use. Homeostatic proliferation plays an important, if not the main, role in maintaining the latent HIV reservoir on ART.[28–35] Blocking the proliferation of HIV-infected CD4$^+$ T cells might prove to be an important target for HIV cure research,[4] perhaps used as a combination approach.[45,50] If CD4$^+$ T cell cycling is largely driven by a cytokine-enriched environment as suggested,[51,52] blocking the proliferation of cells comprising the latent reservoir would be expected to reduce HIV DNA burden over time, particularly if the virus is enriched in cells with high proliferative potential, as appears to be the case.[33,53] Clinical trials designed to interrupt proliferation or inflammatory signaling (e.g., by administration of mycophenolate, JAK-STAT inhibitors)[4,54] may decrease reservoir size. Unlike calcineurin inhibitors (cyclosporine and tacrolimus), which work by blocking T cell receptor-mediated immune signaling, mTOR inhibitors that block cytokine-mediated stimulation may preferentially lead to decreased homeostatic or cytokine-driven proliferation while preserving antigen-specific immune responses.[37,55–57] While CD4$^+$ T cell numbers fell slightly, the disproportionate decrease in frequency of CD4$^+$ T cells containing HIV DNA in this study suggests that CD4$^+$ T cells and the offspring of CD4$^+$ T cells that were susceptible to HIV infection (irrespective of the replication competence of the provirus) remain selectively driven to proliferation and expansion by cytokine exposure.

Whereas the observed decrease of circulating HIV-1 DNA in this study may have been due, in part, to CD4$^+$ T cell redistribution to tissues outside peripheral blood, a recent modeling study suggests that reduced CD4$^+$ T cell cycling and homeostatic proliferation are likely to decrease infected cell burden in both blood and tissues[4] through blocking memory CD4$^+$ T cell generation from HIV-uninfected precursor cells.[51] A recent non-human primate study showing decreases in simian immunodeficiency virus (SIV) DNA following antibody-mediated CD4$^+$ depletion[58] lends support to this putative homeostatic-based mechanism for the sirolimus effect on HIV-1 DNA, fundamentally different than the shock-and-kill therapeutic approach.[52] It is interesting to note that the secondary efficacy population in this study that did not experience significant reductions in CD4$^+$ T cell-associated HIV-1 DNA also did not have decreased markers of T cell cycling at the earlier 4-week time point after initiating sirolimus treatment.

Interestingly, a recent non-human primate study of sirolimus therapy with or without CD3$^+$ T cell depletion did not demonstrate a decrease in overall HIV burden or lead to changes in time to SIV rebound following analytical treatment interruption (ATI), but did demonstrate decreased memory CD4$^+$ T cell proliferation.[50] The reason for the discrepancy between reservoir decay in the SIV model and the significant reductions observed in this human study is not known, but could be due to the duration of ART. In our clinical study, we enrolled individuals who had been on long-term ART. The reservoir is increasingly found in the clonal populations over time,[29,30,53,59] and hence any agent aimed at this mechanism might only become readily detected after many years of virus suppression.

Sirolimus use also led to reduced expression of PD-1 and CCR5 on CD8$^+$ T cells through week 20 of sirolimus (in the primary efficacy population). The PD-1/PD-L1 pathway is implicated in the balance between immune eradication and immune escape and is overexpressed in chronic viral infections such as HIV, even after the initiation of ART.[60–63] As a result, there is interest in using anti-PD-1 therapies to reduce the latent HIV reservoir burden.[64–66] The role of decreased frequencies of CD8$^+$ T cells expressing PD-1 observed in this study is not known and warrants further study, especially as some SEB-specific responses declined with mTOR inhibition and there were no significant changes in HIV-specific responses.

Very few studies have performed a comprehensive assessment of mTOR inhibition in people. As expected, we found that sirolimus had pluripotent effects. We observed changes in cell proliferation (Ki67) and activation (CCR5 and PD-1) as expected, although likely due to small samples size, the effect was significant in only some subsets. We observed a decrease in IP-10, a marker of the interferon response pathway.[67] Surprisingly, we observed a significant increase in circulating markers of inflammation, IL-6, d-Dimer, and sCD14, all of which in untreated and treated HIV predict excess risk of morbidity.[68–74] Data regarding the *in vivo* impact of sirolimus on IL-6 production is evolving, with evidence that mTOR inhibition may reduce IL-6 expression by various antigen-presenting cells, but may also increase expression by others.[75–77] Importantly, D-dimer levels also increased during sirolimus administration, and as these levels are diminished by systemic IL-6 blockade, it is likely that sirolimus administration increases IL-6 expression and bioactivity. As a result, there is some concern that mTOR inhibition may exacerbate pre-existing, persistent HIV-related inflammation, although increases related to a therapeutic intervention will likely have unique implications that are yet to be understood. However, inflammatory markers returned to baseline levels following cessation of sirolimus, and it is possible that the transient increases in immune signaling may have enhanced immune clearance of HIV-infected CD4$^+$ T cells through stimulation of innate or adaptive immune responses.

This study had several limitations. First, the ACTG A5337 study was an open-label, single-arm clinical trial and did not include a randomized placebo group, although a lead-in observation and sampling period was incorporated into the study design to help control for instability in HIV-1 reservoir measures. Also, as aforementioned, only 16 of 30 participants who initiated treatment completed the full 20 weeks. The as-treated population was the basis for primary analyses and sample size estimation, but the frequency of treatment discontinuation was unexpected, although most premature discontinuations were due to low-level events with minimal clinical implications and were driven by either strict protocol definitions or site clinical preference rather than by participant-experienced adverse events. A recent study of everolimus in HIV-infected individuals who underwent solid organ transplant demonstrated that mTOR inhibitor usage in real-world clinical situations was well tolerated without any reported adverse events,[44] with the caveat that this trial had greater tolerance for mild adverse event and laboratory abnormalities, as the drug was being used for an approved clinical indication. Furthermore, low-dose sirolimus is now being trialed in various clinical settings to minimize these adverse events while preserving desired immune-modulating functions.[78,79] Thus, it may play a role in combination HIV cure strategies, including ones that aim

to reduce the proliferative survival of infected cells. Regardless of study dropout, decreases in HIV DNA and changes in inflammatory and immune phenotypes reached statistical significance in this primary analysis population. Furthermore, the study was an open-label, single-arm trial given the need for frequent adjustments of sirolimus dosing and the logistical challenges of including a control group. The 12-week lead-in observation period prior to sirolimus therapy allowed us to determine the stability of the HIV reservoir and baseline immune markers.

In conclusion, this study of mTOR inhibitors in otherwise healthy PWH on long-term, suppressive ART revealed several important significant effects of sirolimus therapy, such as decreasing peripheral blood HIV-1 DNA burden, $CD4^+$ T cell cycling, and $CD8^+$ T cell PD-1 expression, which suggest that immune modulatory therapies may play an important role in ongoing efforts to cure HIV infection in persons with established infection.

Limitations of the study

The ACTG A5337 study was an open-label, single-arm clinical trial and did not include a randomized placebo group. Whereas a lead-in period was incorporated into the study design to take into account HIV-1 reservoir instability over time, incorporation of a placebo arm would have been challenging as some participants underwent frequent dose adjustments to maintain therapeutic and safe levels of sirolimus. Furthermore, the study had a relatively high non-completion rate, although many of the premature discontinuations were not based on clinically significant adverse events. As such, the study lacked statistical power to identify subtle changes in intact proviral DNA or residual low-level HIV-1 RNA at primary and other study time points.

RESOURCE AVAILABILITY

Lead contact

Further information and requests for resources and data should be directed to Timothy J. Henrich (timothy.henrich@ucsf.edu), who will facilitate requests in coordination with the ACTG (Advancing Clinical Therapeutics Globally).

Materials availability

The remaining biological specimens obtained from this clinical trial are maintained and stored by the ACTG. An ACTG New Works Concept Sheet may be filed by investigators who propose access to samples for additional research, which will be reviewed by the ACTG and primary A5337 study team.

Data and code availability

No custom statistical code was designed or used in this study. All non-identifiable data and statistical code will be made available upon request. Individual values are shown in each figure for flow cytometric, ELISA, and PCR assay results. Any additional information required to reanalyze the data reported in this paper is available from the lead contact upon request.

ACKNOWLEDGMENTS

Research reported in this publication was supported by the National Institute of Allergy and Infectious Diseases of the National Institutes of Health under award number UM1 AI068634, UM1 AI068636, UM1 AI106701, UM1 AI069496-08, UM1 AI069412, and K24AI174971-01A1 (to T.J.H.) and ViiV Healthcare. The content is solely the responsibility of the authors and does not necessarily represent the official views of the National Institutes of Health. ClinicialTrials.gov registration #: NCT02440789.

AUTHOR CONTRIBUTIONS

T.J.H.: A5337 study co-chair, clinical trial design and implementation, experiment design and analysis, and wrote the manuscript. P.Y.H.: A5337 study co-chair, clinical trial design and implementation, and conceived the study. S.G.D.: A5337 vice-chair, conceived the study, and clinical trial design and implementation. C.G.: division of AIDS clinical representative, clinical trial design and implementation, and regulatory approvals. R.J.B. and H.M.: clinical trial design and implementation, and data and statistical review and analysis. A.N.: data management. E.H.: clinical trial design and regulatory approvals. M.K. and C.F.: participant enrollment, study site investigators, and clinical evaluations. D.M., B.C., J.Z.L., D.R.K., A.M.B., and M.M.L. designed and performed the experiments and analyzed the data. A.N.D. and F.A. designed and performed pharmacology analyses.

DECLARATION OF INTERESTS

T.J.H. received grant support from Gilead Sciences, Merck, and Bristol Myers Squibb. D.R.K. receives grant support and/or consulting honoraria from AbbVie, Gilead Sciences, GlaxoSmithKline, Janssen, Merck, Roche, and ViiV. J.Z.L. received grant support from Merck.

STAR★METHODS

Detailed methods are provided in the online version of this paper and include the following:

- KEY RESOURCES TABLE
- EXPERIMENTAL MODEL AND SUBJECT DETAILS
  - Study design
  - Inclusion criteria
  - Exclusion criteria
  - Discontinuation/Stopping criteria
  - Sirolimus dosing and drug levels
  - Sample size estimation and biological sample allocations
  - Human samples
  - Study approval
- METHOD DETAILS
  - HIV DNA and RNA quantification
  - HIV single-copy assay
  - Plasma inflammation markers
  - Flow cytometric analyses
  - *In vitro* stimulations
- QUANTIFICATION AND STATISTICAL ANALYSIS
  - HIV RNA and DNA
  - IPDA
  - Flow cytometric analyses
- ADDITIONAL RESOURCES

SUPPLEMENTAL INFORMATION

Supplemental information can be found online at https://doi.org/10.1016/j.xcrm.2024.101745.

Received: November 12, 2023
Revised: April 10, 2024
Accepted: August 28, 2024
Published: September 24, 2024

REFERENCES

1. Klatt, N.R., Chomont, N., Douek, D.C., and Deeks, S.G. (2013). Immune activation and HIV persistence: implications for curative approaches to HIV infection. Immunol. Rev. *254*, 326–342. https://doi.org/10.1111/imr.12065.

2. International AIDS Society Scientific Working Group on HIV Cure; Deeks, S.G., Autran, B., Berkhout, B., Benkirane, M., Cairns, S., Chomont, N., Chun, T.W., Churchill, M., Di Mascio, M., et al. (2012). Towards an HIV cure: a global scientific strategy. Nat. Rev. Immunol. *12*, 607–614, nri3262 [pii]. https://doi.org/10.1038/nri3262.

3. Deeks, S.G., Archin, N., Cannon, P., Collins, S., Jones, R.B., de Jong, M.A.W.P., Lambotte, O., Lamplough, R., Ndung'u, T., Sugarman, J., et al. (2021). Research priorities for an HIV cure: International AIDS Society Global Scientific Strategy 2021. Nat. Med. *27*, 2085–2098. https://doi.org/10.1038/s41591-021-01590-5.

4. Reeves, D.B., Duke, E.R., Hughes, S.M., Prlic, M., Hladik, F., and Schiffer, J.T. (2017). Anti-proliferative therapy for HIV cure: a compound interest approach. Sci. Rep. *7*, 4011. https://doi.org/10.1038/s41598-017-04160-3.

5. Gamper, C.J., and Powell, J.D. (2012). All PI3Kinase signaling is not mTOR: dissecting mTOR-dependent and independent signaling pathways in T cells. Front. Immunol. *3*, 312. https://doi.org/10.3389/fimmu.2012.00312.

6. Isotani, S., Hara, K., Tokunaga, C., Inoue, H., Avruch, J., and Yonezawa, K. (1999). Immunopurified mammalian target of rapamycin phosphorylates and activates p70 S6 kinase alpha in vitro. J. Biol. Chem. *274*, 34493–34498.

7. Moes, D.J.A.R., Guchelaar, H.J., and de Fijter, J.W. (2015). Sirolimus and everolimus in kidney transplantation. Drug Discov. Today *20*, 1243–1249, S1359-6446(15)00196-8 [pii]. https://doi.org/10.1016/j.drudis.2015.05.006.

8. Cutler, C., and Antin, J.H. (2004). Sirolimus for GVHD prophylaxis in allogeneic stem cell transplantation. Bone Marrow Transplant. *34*, 471–476. https://doi.org/10.1038/sj.bmt.1704604. 1704604 [pii].

9. Cutler, C., and Antin, J.H. (2010). Sirolimus immunosuppression for graft-versus-host disease prophylaxis and therapy: an update. Curr. Opin. Hematol. *17*, 500–504. https://doi.org/10.1097/MOH.0b013e32833e5b2e.

10. De Simone, P., Fagiuoli, S., Cescon, M., De Carlis, L., Tisone, G., Volpes, R., and Cillo, U. (2017). Use of Everolimus in Liver Transplantation: Recommendations From a Working Group. Transplantation *101*, 239–251. https://doi.org/10.1097/TP.0000000000001438.

11. Di Benedetto, F., Di Sandro, S., De Ruvo, N., Montalti, R., Ballarin, R., Guerrini, G.P., Spaggiari, M., Guaraldi, G., and Gerunda, G. (2010). First report on a series of HIV patients undergoing rapamycin monotherapy after liver transplantation. Transplantation *89*, 733–738. https://doi.org/10.1097/TP.0b013e3181c7dcc0.

12. Donia, M., McCubrey, J.A., Bendtzen, K., and Nicoletti, F. (2010). Potential use of rapamycin in HIV infection. Br. J. Clin. Pharmacol. *70*, 784–793. https://doi.org/10.1111/j.1365-2125.2010.03735.x.

13. Samonakis, D.N., Cholongitas, E., Triantos, C.K., Griffiths, P., Dhillon, A.P., Thalheimer, U., Patch, D.W., and Burroughs, A.K. (2005). Sustained, spontaneous disappearance of serum HCV-RNA under immunosuppression after liver transplantation for HCV cirrhosis. J. Hepatol. *43*, 1091–1093, S0168-8278(05)00563-5 [pii]. https://doi.org/10.1016/j.jhep.2005.08.005.

14. Stock, P.G., Barin, B., Hatano, H., Rogers, R.L., Roland, M.E., Lee, T.H., Busch, M., and Deeks, S.G.; for Solid Organ Transplantation in HIV Study Investigators (2014). Reduction of HIV persistence following transplantation in HIV-infected kidney transplant recipients. Am. J. Transplant. *14*, 1136–1141. https://doi.org/10.1111/ajt.12699.

15. Oswald-Richter, K., Grill, S.M., Leelawong, M., and Unutmaz, D. (2004). HIV infection of primary human T cells is determined by tunable thresholds of T cell activation. Eur. J. Immunol. *34*, 1705–1714. https://doi.org/10.1002/eji.200424892.

16. Roy, J., Paquette, J.S., Fortin, J.F., and Tremblay, M.J. (2002). The immunosuppressant rapamycin represses human immunodeficiency virus type 1 replication. Antimicrob. Agents Chemother. *46*, 3447–3455.

17. Heredia, A., Latinovic, O., Gallo, R.C., Melikyan, G., Reitz, M., Le, N., and Redfield, R.R. (2008). Reduction of CCR5 with low-dose rapamycin enhances the antiviral activity of vicriviroc against both sensitive and drug-resistant HIV-1. Proc. Natl. Acad. Sci. USA *105*, 20476–20481, 0810843106 [pii]. https://doi.org/10.1073/pnas.0810843106.

18. Heredia, A., Le, N., Gartenhaus, R.B., Sausville, E., Medina-Moreno, S., Zapata, J.C., Davis, C., Gallo, R.C., and Redfield, R.R. (2015). Targeting of mTOR catalytic site inhibits multiple steps of the HIV-1 lifecycle and suppresses HIV-1 viremia in humanized mice. Proc. Natl. Acad. Sci. USA *112*, 9412–9417, 1511144112 [pii]. https://doi.org/10.1073/pnas.1511144112.

19. Coppock, J.D., Wieking, B.G., Molinolo, A.A., Gutkind, J.S., Miskimins, W.K., and Lee, J.H. (2013). Improved clearance during treatment of HPV-positive head and neck cancer through mTOR inhibition. Neoplasia *15*, 620–630.

20. Molinolo, A.A., Marsh, C., El Dinali, M., Gangane, N., Jennison, K., Hewitt, S., Patel, V., Seiwert, T.Y., and Gutkind, J.S. (2012). mTOR as a molecular target in HPV-associated oral and cervical squamous carcinomas. Clin. Cancer Res. *18*, 2558–2568, 1078-0432.CCR-11-2824 [pii]. https://doi.org/10.1158/1078-0432.CCR-11-2824.

21. Keating, R., Hertz, T., Wehenkel, M., Harris, T.L., Edwards, B.A., McClaren, J.L., Brown, S.A., Surman, S., Wilson, Z.S., Bradley, P., et al. (2013). The kinase mTOR modulates the antibody response to provide cross-protective immunity to lethal infection with influenza virus. Nat. Immunol. *14*, 1266–1276, ni.2741 [pii]. https://doi.org/10.1038/ni.2741.

22. Krown, S.E., Roy, D., Lee, J.Y., Dezube, B.J., Reid, E.G., Venkataramanan, R., Han, K., Cesarman, E., and Dittmer, D.P. (2012). Rapamycin with antiretroviral therapy in AIDS-associated Kaposi sarcoma: an AIDS Malignancy Consortium study. J. Acquir. Immune Defic. Syndr. *59*, 447–454. https://doi.org/10.1097/QAI.0b013e31823e7884.

23. Kern, F., Faulhaber, N., Frömmel, C., Khatamzas, E., Prösch, S., Schönemann, C., Kretzschmar, I., Volkmer-Engert, R., Volk, H.D., and Reinke, P. (2000). Analysis of CD8 T cell reactivity to cytomegalovirus using protein-spanning pools of overlapping pentadecapeptides. Eur. J. Immunol. *30*, 1676–1682. https://doi.org/10.1002/1521-4141(200006)30:6<1676::AID-IMMU1676>3.0.CO;2-V.

24. Mhatre, M., McAndrew, T., Carpenter, C., Burk, R.D., Einstein, M.H., and Herold, B.C. (2012). Cervical intraepithelial neoplasia is associated with genital tract mucosal inflammation. Sex. Transm. Dis. *39*, 591–597, 00007435-201208000-00004 [pii]. https://doi.org/10.1097/OLQ.0b013e318255aeef.

25. Gilliam, B.L., Heredia, A., Devico, A., Le, N., Bamba, D., Bryant, J.L., Pauza, C.D., and Redfield, R.R. (2007). Rapamycin reduces CCR5 mRNA levels in macaques: potential applications in HIV-1 prevention and treatment. AIDS *21*, 2108–2110, 00002030-200710010-00016 [pii]. https://doi.org/10.1097/QAD.0b013e3282f02a4f.

26. Heredia, A., Amoroso, A., Davis, C., Le, N., Reardon, E., Dominique, J.K., Klingebiel, E., Gallo, R.C., and Redfield, R.R. (2003). Rapamycin causes down-regulation of CCR5 and accumulation of anti-HIV beta-chemokines: an approach to suppress R5 strains of HIV-1. Proc. Natl. Acad. Sci. USA *100*, 10411–10416. https://doi.org/10.1073/pnas.1834278100. 1834278100 [pii].

27. Ponticelli, C. (2014). The pros and the cons of mTOR inhibitors in kidney transplantation. Expet Rev. Clin. Immunol. *10*, 295–305. https://doi.org/10.1586/1744666X.2014.872562.

28. Chomont, N., El-Far, M., Ancuta, P., Trautmann, L., Procopio, F.A., Yassine-Diab, B., Boucher, G., Boulassel, M.R., Ghattas, G., Brenchley, J.M., et al. (2009). HIV reservoir size and persistence are driven by T cell survival and homeostatic proliferation. Nat. Med. *15*, 893–900, nm.1972 [pii]. https://doi.org/10.1038/nm.1972.

29. Wagner, T.A., McLaughlin, S., Garg, K., Cheung, C.Y.K., Larsen, B.B., Styrchak, S., Huang, H.C., Edlefsen, P.T., Mullins, J.I., and Frenkel, L.M. (2014). HIV latency. Proliferation of cells with HIV integrated into cancer genes contributes to persistent infection. Science *345*, 570–573, science.1256304 [pii]. https://doi.org/10.1126/science.1256304.

30. Maldarelli, F., Wu, X., Su, L., Simonetti, F.R., Shao, W., Hill, S., Spindler, J., Ferris, A.L., Mellors, J.W., Kearney, M.F., et al. (2014). HIV latency. Specific HIV integration sites are linked to clonal expansion and persistence

CellPress
OPEN ACCESS

of infected cells. Science *345*, 179–183, science.1254194 [pii]. https://doi.org/10.1126/science.1254194.

31. Mendoza, P., Jackson, J.R., Oliveira, T.Y., Gaebler, C., Ramos, V., Caskey, M., Jankovic, M., Nussenzweig, M.C., and Cohn, L.B. (2020). Antigen-responsive CD4+ T cell clones contribute to the HIV-1 latent reservoir. J. Exp. Med. *217*, e20200051. https://doi.org/10.1084/jem.20200051.
32. Simonetti, F.R., Zhang, H., Soroosh, G.P., Duan, J., Rhodehouse, K., Hill, A.L., Beg, S.A., McCormick, K., Raymond, H.E., Nobles, C.L., et al. (2021). Antigen-driven clonal selection shapes the persistence of HIV-1-infected CD4+ T cells in vivo. J. Clin. Invest. *131*, e145254. https://doi.org/10.1172/JCI145254.
33. Bacchus-Souffan, C., Fitch, M., Symons, J., Abdel-Mohsen, M., Reeves, D.B., Hoh, R., Stone, M., Hiatt, J., Kim, P., Chopra, A., et al. (2021). Relationship between CD4 T cell turnover, cellular differentiation and HIV persistence during ART. PLoS Pathog. *17*, e1009214. https://doi.org/10.1371/journal.ppat.1009214.
34. Hiener, B., Horsburgh, B.A., Eden, J.S., Barton, K., Schlub, T.E., Lee, E., von Stockenstrom, S., Odevall, L., Milush, J.M., Liegler, T., et al. (2017). Identification of Genetically Intact HIV-1 Proviruses in Specific CD4+ T Cells from Effectively Treated Participants. Cell Rep. *21*, 813–822, S2211-1247(17)31386-4 [pii]. https://doi.org/10.1016/j.celrep.2017.09.081.
35. Gantner, P., Pagliuzza, A., Pardons, M., Ramgopal, M., Routy, J.P., Fromentin, R., and Chomont, N. (2020). Single-cell TCR sequencing reveals phenotypically diverse clonally expanded cells harboring inducible HIV proviruses during ART. Nat. Commun. *11*, 4089. https://doi.org/10.1038/s41467-020-17898-8.
36. Battaglia, M., Stabilini, A., and Roncarolo, M.G. (2005). Rapamycin selectively expands CD4+CD25+FoxP3+ regulatory T cells. Blood *105*, 4743–4748, 2004-10-3932 [pii]. https://doi.org/10.1182/blood-2004-10-3932.
37. Wiederrecht, G.J., Sabers, C.J., Brunn, G.J., Martin, M.M., Dumont, F.J., and Abraham, R.T. (1995). Mechanism of action of rapamycin: new insights into the regulation of G1-phase progression in eukaryotic cells. Prog. Cell Cycle Res. *1*, 53–71.
38. Jiang, W., Younes, S.A., Funderburg, N.T., Mudd, J.C., Espinosa, E., Davenport, M.P., Babineau, D.C., Sieg, S.F., and Lederman, M.M. (2014). Cycling memory CD4+ T cells in HIV disease have a diverse T cell receptor repertoire and a phenotype consistent with bystander activation. J. Virol. *88*, 5369–5380. https://doi.org/10.1128/JVI.00017-14.
39. Younes, S.A., Freeman, M.L., Mudd, J.C., Shive, C.L., Reynaldi, A., Panigrahi, S., Estes, J.D., Deleage, C., Lucero, C., Anderson, J., et al. (2016). IL-15 promotes activation and expansion of CD8+ T cells in HIV-1 infection. J. Clin. Invest. *126*, 2745–2756. https://doi.org/10.1172/JCI85996.
40. Morris, S.R., Chen, B., Mudd, J.C., Panigrahi, S., Shive, C.L., Sieg, S.F., Cameron, C.M., Zidar, D.A., Funderburg, N.T., Younes, S.A., et al. (2020). Inflammescent CX3CR1+CD57+CD8+ T cells are generated and expanded by IL-15. JCI Insight *5*, e132963. https://doi.org/10.1172/jci.insight.132963.
41. Bruner, K.M., Wang, Z., Simonetti, F.R., Bender, A.M., Kwon, K.J., Sengupta, S., Fray, E.J., Beg, S.A., Antar, A.A.R., Jenike, K.M., et al. (2019). A quantitative approach for measuring the reservoir of latent HIV-1 proviruses. Nature *566*, 120–125, 10.1038/s41586-019-0898-8 [pii]. https://doi.org/10.1038/s41586-019-0898-8.
42. Henrich, T.J., Hanhauser, E., Marty, F.M., Sirignano, M.N., Keating, S., Lee, T.H., Robles, Y.P., Davis, B.T., Li, J.Z., Heisey, A., et al. (2014). Antiretroviral-Free HIV-1 Remission and Viral Rebound After Allogeneic Stem Cell Transplantation: Report of 2 Cases. Ann. Intern. Med. *161*, 319–327, [pii]. https://doi.org/10.7326/M14-1027.
43. Henrich, T.J., Hatano, H., Bacon, O., Hogan, L.E., Rutishauser, R., Hill, A., Kearney, M.F., Anderson, E.M., Buchbinder, S.P., Cohen, S.E., et al. (2017). HIV-1 persistence following extremely early initiation of antiretroviral therapy (ART) during acute HIV-1 infection: An observational study. PLoS Med. *14*, e1002417, PMEDICINE-D-17-02137 [pii]. https://doi.org/10.1371/journal.pmed.1002417.
44. Henrich, T.J., Schreiner, C., Cameron, C., Hogan, L.E., Richardson, B., Rutishauser, R.L., Deitchman, A.N., Chu, S., Rogers, R., Thanh, C., et al. (2021). Everolimus, an mTORC1/2 inhibitor, in ART-suppressed individuals who received solid organ transplantation: A prospective study. Am. J. Transplant. *21*, 1765–1779. https://doi.org/10.1111/ajt.16244.
45. Martin, A.R., Pollack, R.A., Capoferri, A., Ambinder, R.F., Durand, C.M., and Siliciano, R.F. (2017). Rapamycin-mediated mTOR inhibition uncouples HIV-1 latency reversal from cytokine-associated toxicity. J. Clin. Invest. *127*, 651–656, 89552 [pii]. https://doi.org/10.1172/JCI89552.
46. Araki, K., Turner, A.P., Shaffer, V.O., Gangappa, S., Keller, S.A., Bachmann, M.F., Larsen, C.P., and Ahmed, R. (2009). mTOR regulates memory CD8 T-cell differentiation. Nature *460*, 108–112, nature08155 [pii]. https://doi.org/10.1038/nature08155.
47. Ho, Y.-C., Shan, L., Hosmane, N.N., Wang, J., Laskey, S.B., Rosenbloom, D.I.S., Lai, J., Blankson, J.N., Siliciano, J.D., and Siliciano, R.F. (2013). Replication-Competent Noninduced Proviruses in the Latent Reservoir Increase Barrier to HIV-1 Cure. Cell *155*, 540–551.
48. Gandhi, R.T., Cyktor, J.C., Bosch, R.J., Mar, H., Laird, G.M., Martin, A., Collier, A.C., Riddler, S.A., Macatangay, B.J., Rinaldo, C.R., et al. (2021). Selective Decay of Intact HIV-1 Proviral DNA on Antiretroviral Therapy. J. Infect. Dis. *223*, 225–233. https://doi.org/10.1093/infdis/jiaa532.
49. Gandhi, R.T., McMahon, D.K., Bosch, R.J., Lalama, C.M., Cyktor, J.C., Macatangay, B.J., Rinaldo, C.R., Riddler, S.A., Hogg, E., Godfrey, C., et al. (2017). Levels of HIV-1 persistence on antiretroviral therapy are not associated with markers of inflammation or activation. PLoS Pathog. *13*, e1006285. https://doi.org/10.1371/journal.ppat.1006285.
50. Varco-Merth, B.D., Brantley, W., Marenco, A., Duell, D.D., Fachko, D.N., Richardson, B., Busman-Sahay, K., Shao, D., Flores, W., Engelman, K., et al. (2022). Rapamycin limits CD4+ T cell proliferation in simian immunodeficiency virus-infected rhesus macaques on antiretroviral therapy. J. Clin. Invest. *132*, e156063. https://doi.org/10.1172/JCI156063.
51. Grossman, Z., Singh, N.J., Simonetti, F.R., Lederman, M.M., Douek, D.C., and Deeks, S.G.; Contributing authors (2020). Rinse and Replace': Boosting T Cell Turnover To Reduce HIV-1 Reservoirs. Trends Immunol. *41*, 466–480. https://doi.org/10.1016/j.it.2020.04.003.
52. Sadowski, I., and Hashemi, F.B. (2019). Strategies to eradicate HIV from infected patients: elimination of latent provirus reservoirs. Cell. Mol. Life Sci. *76*, 3583–3600. https://doi.org/10.1007/s00018-019-03156-8.
53. Lee, E., Bacchetti, P., Milush, J., Shao, W., Boritz, E., Douek, D., Fromentin, R., Liegler, T., Hoh, R., Deeks, S.G., et al. (2019). Memory CD4 + T-Cells Expressing HLA-DR Contribute to HIV Persistence During Prolonged Antiretroviral Therapy. Front. Microbiol. *10*, 2214. https://doi.org/10.3389/fmicb.2019.02214.
54. Pitman, M.C., Lau, J.S.Y., McMahon, J.H., and Lewin, S.R. (2018). Barriers and strategies to achieve a cure for HIV. Lancet. HIV *5*, e317–e328. https://doi.org/10.1016/S2352-3018(18)30039-0.
55. Lee, Y.R., Yang, I.H., Lee, Y.H., Im, S.A., Song, S., Li, H., Han, K., Kim, K., Eo, S.K., and Lee, C.K. (2005). Cyclosporin A and tacrolimus, but not rapamycin, inhibit MHC-restricted antigen presentation pathways in dendritic cells. Blood *105*, 3951–3955, 2004-10-3927 [pii]. https://doi.org/10.1182/blood-2004-10-3927.
56. Baan, C.C., van der Mast, B.J., Klepper, M., Mol, W.M., Peeters, A.M.A., Korevaar, S.S., Balk, A.H.M.M., and Weimar, W. (2005). Differential effect of calcineurin inhibitors, anti-CD25 antibodies and rapamycin on the induction of FOXP3 in human T cells. Transplantation *80*, 110–117. 00007890-200507150-00019 [pii].
57. Coenen, J.J.A., Koenen, H.J.P.M., van Rijssen, E., Hilbrands, L.B., and Joosten, I. (2006). Rapamycin, and not cyclosporin A, preserves the highly suppressive CD27+ subset of human CD4+CD25+ regulatory T cells. Blood *107*, 1018–1023, 2005-07-3032 [pii]. https://doi.org/10.1182/blood-2005-07-3032.
58. Kumar, N.A., McBrien, J.B., Carnathan, D.G., Mavigner, M., Mattingly, C., White, E.R., Viviano, F., Bosinger, S.E., Chahroudi, A., Silvestri, G., et al. (2018). Antibody-Mediated CD4 Depletion Induces Homeostatic CD4(+)

T Cell Proliferation without Detectable Virus Reactivation in Antiretroviral Therapy-Treated Simian Immunodeficiency Virus-Infected Macaques. J. Virol. *92*, e01235-18. https://doi.org/10.1128/JVI.01235-18.

59. Cohn, L.B., Silva, I.T., Oliveira, T.Y., Rosales, R.A., Parrish, E.H., Learn, G.H., Hahn, B.H., Czartoski, J.L., McElrath, M.J., Lehmann, C., et al. (2015). HIV-1 integration landscape during latent and active infection. Cell *160*, 420–432, S0092-8674(15)00063-X [pii]. https://doi.org/10.1016/j.cell.2015.01.020.
60. Velu, V., Titanji, K., Zhu, B., Husain, S., Pladevega, A., Lai, L., Vanderford, T.H., Chennareddi, L., Silvestri, G., Freeman, G.J., et al. (2009). Enhancing SIV-specific immunity in vivo by PD-1 blockade. Nature *458*, 206–210, nature07662 [pii]. https://doi.org/10.1038/nature07662.
61. Trautmann, L., Janbazian, L., Chomont, N., Said, E.A., Gimmig, S., Bessette, B., Boulassel, M.R., Delwart, E., Sepulveda, H., Balderas, R.S., et al. (2006). Upregulation of PD-1 expression on HIV-specific CD8+ T cells leads to reversible immune dysfunction. Nat. Med. *12*, 1198–1202, nm1482 [pii]. https://doi.org/10.1038/nm1482.
62. Day, C.L., Kaufmann, D.E., Kiepiela, P., Brown, J.A., Moodley, E.S., Reddy, S., Mackey, E.W., Miller, J.D., Leslie, A.J., DePierres, C., et al. (2006). PD-1 expression on HIV-specific T cells is associated with T-cell exhaustion and disease progression. Nature *443*, 350–354. https://doi.org/10.1038/nature05115.
63. Rinaldi, S., de Armas, L., Dominguez-Rodríguez, S., Pallikkuth, S., Dinh, V., Pan, L., Gärtner, K., Pahwa, R., Cotugno, N., Rojo, P., et al. (2021). T cell immune discriminants of HIV reservoir size in a pediatric cohort of perinatally infected individuals. PLoS Pathog. *17*, e1009533. https://doi.org/10.1371/journal.ppat.1009533.
64. Scully, E.P., Rutishauser, R.L., Simoneau, C.R., Delagrèverie, H., Euler, Z., Thanh, C., Li, J.Z., Hartig, H., Bakkour, S., Busch, M., et al. (2018). Inconsistent HIV reservoir dynamics and immune responses following anti-PD-1 therapy in cancer patients with HIV infection. Ann. Oncol. *29*, 2141–2142, 5055604 [pii]. https://doi.org/10.1093/annonc/mdy259.
65. Uldrick, T.S., Adams, S.V., Fromentin, R., Roche, M., Fling, S.P., Gonçalves, P.H., Lurain, K., Ramaswami, R., Wang, C.C.J., Gorelick, R.J., et al. (2022). Pembrolizumab induces HIV latency reversal in people living with HIV and cancer on antiretroviral therapy. Sci. Transl. Med. *14*, eabl3836. https://doi.org/10.1126/scitranslmed.abl3836.
66. Guihot, A., Marcelin, A.G., Massiani, M.A., Samri, A., Soulié, C., Autran, B., and Spano, J.P. (2018). Drastic decrease of the HIV reservoir in a patient treated with nivolumab for lung cancer. Ann. Oncol. *29*, 517–518, 4677259 [pii]. https://doi.org/10.1093/annonc/mdx696.
67. Lei, J., Yin, X., Shang, H., and Jiang, Y. (2019). IP-10 is highly involved in HIV infection. Cytokine *115*, 97–103. https://doi.org/10.1016/j.cyto.2018.11.018.
68. Borges, Á.H., Silverberg, M.J., Wentworth, D., Grulich, A.E., Fätkenheuer, G., Mitsuyasu, R., Tambussi, G., Sabin, C.A., Neaton, J.D., Lundgren, J.D., et al. (2013). Predicting risk of cancer during HIV infection: the role of inflammatory and coagulation biomarkers. AIDS *27*, 1433–1441, 00002030-201306010-00009 [pii]. https://doi.org/10.1097/QAD.0b013e32835f6b0c.
69. Tenorio, A.R., Zheng, Y., Bosch, R.J., Krishnan, S., Rodriguez, B., Hunt, P.W., Plants, J., Seth, A., Wilson, C.C., Deeks, S.G., et al. (2014). Soluble markers of inflammation and coagulation but not T-cell activation predict non-AIDS-defining morbid events during suppressive antiretroviral treatment. J. Infect. Dis. *210*, 1248–1259, jiu254 [pii]. https://doi.org/10.1093/infdis/jiu254.
70. Sereti, I., Krebs, S.J., Phanuphak, N., Fletcher, J.L., Slike, B., Pinyakorn, S., O'Connell, R.J., Rupert, A., Chomont, N., Valcour, V., et al. (2017). Persistent, Albeit Reduced, Chronic Inflammation in Persons Starting Antiretroviral Therapy in Acute HIV Infection. Clin. Infect. Dis. *64*, 124–131, ciw683 [pii]. https://doi.org/10.1093/cid/ciw683.
71. Tawakol, A., Ishai, A., Li, D., Takx, R.A.P., Hur, S., Kaiser, Y., Pampaloni, M., Rupert, A., Hsu, D., Sereti, I., et al. (2017). Association of Arterial and Lymph Node Inflammation With Distinct Inflammatory Pathways in Human Immunodeficiency Virus Infection. JAMA Cardiol. *2*, 163–171, 2590066 [pii]. https://doi.org/10.1001/jamacardio.2016.4728.
72. Lederman, M.M., Calabrese, L., Funderburg, N.T., Clagett, B., Medvik, K., Bonilla, H., Gripshover, B., Salata, R.A., Taege, A., Lisgaris, M., et al. (2011). Immunologic failure despite suppressive antiretroviral therapy is related to activation and turnover of memory CD4 cells. J. Infect. Dis. *204*, 1217–1226, jir507 [pii]. https://doi.org/10.1093/infdis/jir507.
73. Baker, J.V., Neuhaus, J., Duprez, D., Kuller, L.H., Tracy, R., Belloso, W.H., De Wit, S., Drummond, F., Lane, H.C., Ledergerber, B., et al. (2011). Changes in inflammatory and coagulation biomarkers: a randomized comparison of immediate versus deferred antiretroviral therapy in patients with HIV infection. J. Acquir. Immune Defic. Syndr. *56*, 36–43. https://doi.org/10.1097/QAI.0b013e3181f7f61a.
74. Crowell, T.A., Fletcher, J.L., Sereti, I., Pinyakorn, S., Dewar, R., Krebs, S.J., Chomchey, N., Rerknimitr, R., Schuetz, A., Michael, N.L., et al. (2016). Initiation of antiretroviral therapy before detection of colonic infiltration by HIV reduces viral reservoirs, inflammation and immune activation. J. Int. AIDS Soc. *19*, 21163. 21163 [pii].
75. Ekshyyan, O., Khandelwal, A.R., Rong, X., Moore-Medlin, T., Ma, X., Alexander, J.S., and Nathan, C.A.O. (2016). Rapamycin targets Interleukin 6 (IL-6) expression and suppresses endothelial cell invasion stimulated by tumor cells. Am. J. Transl. Res. *8*, 4822–4830.
76. Zhang, Y., He, X., Wu, X., Lei, M., Wei, Z., Zhang, X., Wen, L., Xu, P., Li, S., and Qu, S. (2017). Rapamycin upregulates glutamate transporter and IL-6 expression in astrocytes in a mouse model of Parkinson's disease. Cell Death Dis. *8*, e2611. https://doi.org/10.1038/cddis.2016.491.
77. Weichhart, T., Hengstschläger, M., and Linke, M. (2015). Regulation of innate immune cell function by mTOR. Nat. Rev. Immunol. *15*, 599–614. https://doi.org/10.1038/nri3901.
78. Parker, V.E.R., Keppler-Noreuil, K.M., Faivre, L., Luu, M., Oden, N.L., De Silva, L., Sapp, J.C., Andrews, K., Bardou, M., Chen, K.Y., et al. (2019). Safety and efficacy of low-dose sirolimus in the PIK3CA-related overgrowth spectrum. Genet. Med. *21*, 1189–1198. https://doi.org/10.1038/s41436-018-0297-9.
79. Wen, H.Y., Wang, J., Zhang, S.X., Luo, J., Zhao, X.C., Zhang, C., Wang, C.H., Hu, F.Y., Zheng, X.J., Cheng, T., et al. (2019). Low-Dose Sirolimus Immunoregulation Therapy in Patients with Active Rheumatoid Arthritis: A 24-Week Follow-Up of the Randomized, Open-Label, Parallel-Controlled Trial. J. Immunol. Res. *2019*, 7684352. https://doi.org/10.1155/2019/7684352.
80. Li, J.Z., Etemad, B., Ahmed, H., Aga, E., Bosch, R.J., Mellors, J.W., Kuritzkes, D.R., Lederman, M.M., Para, M., and Gandhi, R.T. (2016). The size of the expressed HIV reservoir predicts timing of viral rebound after treatment interruption. AIDS *30*, 343–353. https://doi.org/10.1097/QAD.0000000000000953.
81. Ledderose, C., Heyn, J., Limbeck, E., and Kreth, S. (2011). Selection of reliable reference genes for quantitative real-time PCR in human T cells and neutrophils. BMC Res. Notes *4*, 427. https://doi.org/10.1186/1756-0500-4-427.
82. Malnati, M.S., Scarlatti, G., Gatto, F., Salvatori, F., Cassina, G., Rutigliano, T., Volpi, R., and Lusso, P. (2008). A universal real-time PCR assay for the quantification of group-M HIV-1 proviral load. Nat. Protoc. *3*, 1240–1248, nprot.2008.108 [pii]. https://doi.org/10.1038/nprot.2008.108.
83. Cillo, A.R., Vagratian, D., Bedison, M.A., Anderson, E.M., Kearney, M.F., Fyne, E., Koontz, D., Coffin, J.M., Piatak, M., Jr., and Mellors, J.W. (2014). Improved single-copy assays for quantification of persistent HIV-1 viremia in patients on suppressive antiretroviral therapy. J. Clin. Microbiol. *52*, 3944–3951, JCM.02060-14 [pii]. https://doi.org/10.1128/JCM.02060-14.
84. Li, J.Z., Heisey, A., Ahmed, H., Wang, H., Zheng, L., Carrington, M., Wrin, T., Schooley, R.T., Lederman, M.M., and Kuritzkes, D.R.; ACTG A5197 Study Team (2014). Relationship of HIV reservoir characteristics with immune status and viral rebound kinetics in an HIV therapeutic vaccine study. AIDS *28*, 2649–2657. https://doi.org/10.1097/QAD.0000000000000478.

CellPress
OPEN ACCESS

85. Henrich, T., Hu, Z., Li, J., Sciaranghella, G., Busch, M., Keating, S., Gallien, S., Lin, N., Giguel, F., Lavoie, L., et al. (2013). Long-Term Reduction in Peripheral Blood HIV-1 Reservoirs Following Reduced-Intensity Conditioning Allogeneic Stem Cell Transplantation. JID (J. Infect. Dis.) *207*, 1694–1702. *In Press*.

86. Li, J.Z., Gallien, S., Ribaudo, H., Heisey, A., Bangsberg, D.R., and Kuritzkes, D.R. (2014). Incomplete adherence to antiretroviral therapy is associated with higher levels of residual HIV-1 viremia. AIDS *28*, 181–186. https://doi.org/10.1097/QAD.0000000000000123.

87. Salantes, D.B., Zheng, Y., Mampe, F., Srivastava, T., Beg, S., Lai, J., Li, J.Z., Tressler, R.L., Koup, R.A., Hoxie, J., et al. (2018). HIV-1 latent reservoir size and diversity are stable following brief treatment interruption. J. Clin. Invest. *128*, 3102–3115, 120194 [pii]. https://doi.org/10.1172/JCI120194.

88. Palmer, S., Wiegand, A.P., Maldarelli, F., Bazmi, H., Mican, J.M., Polis, M., Dewar, R.L., Planta, A., Liu, S., Metcalf, J.A., et al. (2003). New real-time reverse transcriptase-initiated PCR assay with single-copy sensitivity for human immunodeficiency virus type 1 RNA in plasma. J. Clin. Microbiol. *41*, 4531–4536.

89. Kern, F., Bunde, T., Faulhaber, N., Kiecker, F., Khatamzas, E., Rudawski, I.M., Pruss, A., Gratama, J.W., Volkmer-Engert, R., Ewert, R., et al. (2002). Cytomegalovirus (CMV) phosphoprotein 65 makes a large contribution to shaping the T cell repertoire in CMV-exposed individuals. J. Infect. Dis. *185*, 1709–1716. https://doi.org/10.1086/340637.

90. Maecker, H.T., Dunn, H.S., Suni, M.A., Khatamzas, E., Pitcher, C.J., Bunde, T., Persaud, N., Trigona, W., Fu, T.M., Sinclair, E., et al. (2001). Use of overlapping peptide mixtures as antigens for cytokine flow cytometry. J. Immunol. Methods *255*, 27–40. https://doi.org/10.1016/s0022-1759(01)00416-1.

91. Vaida, F., and Liu, L. (2009). Fast Implementation for Normal Mixed Effects Models With Censored Response. J. Comput. Graph Stat. *18*, 797–817. https://doi.org/10.1198/jcgs.2009.07130.

STAR★METHODS

KEY RESOURCES TABLE

REAGENT or RESOURCE	SOURCE	IDENTIFIER
**Antibodies**		
LIVE/DEAD Fixable Aqua Dead Cell Stain Kit	Invitrogen	Cat# L34957
Anti-CD3 BV711 Clone UCHT1	BD Biosciences	Cat# 563725; RRID: AB_2744392
Anti-CD4 BUV395 Clone SK3	BD Biosciences	Cat# 563552; RRID: AB_2738275
Anti-CD8 APC-Cy7 Clone SK1	BioLegend	Cat# 344714; RRID: AB_2044006
Anti-IFN-γ PE-Cy7 Clone B27	BioLegend	Cat# 506518; RRID: AB_2123321
Anti-TNF-α FITC Mab11	BioLegend	Cat# 502906; RRID: AB_315258
Anti-IL-2 APC MQ1-17H12	BioLegend	Cat# 500310; RRID: AB_315097
Anti-CD40L/CD154 24-31	BioLegend	Cat# 310802; RRID: AB_314825
Anti-MIP-1β PE D21-1351	BD Biosciences	Cat# 550078; RRID: AB_393549
Brefeldin A	BioLegend	Cat# 420601
CD107α PE-CF594 H4A3	BD Biosciences	Cat# 562628; RRID: AB_2737686
**Biological samples**		
Human peripheral blood mononuclear cells	Human participants	This manuscript
Human plasma	Human participants	This manuscript
**Chemicals, peptides, and recombinant proteins**		
HIV-1 PTE Gag Peptide Set	NIH AIDS Reagent Program	Cat# 11554
HCMV pp65 Peptide Pool	NIH AIDS Reagent Program	Cat# 11549
Cytofix/Cytoperm	BD Biosciences	Cat# 554722
SEB	Toxin Technology Inc.	Cat# NC9442400
RPMI1640	Gibco	Cat# 11875101
Fetal Calf Serum	Sigma	Cat# F4135
Benzonase	EMD Millipore	Cat# 1.01695.0001
L-glutamine	Gibco	Cat# 25030081
Penicillin and Streptomycin	Gibco	Cat# 15140122
Quantikine ELISA kit	R&D Systems	Cat# DC140
Quantikine ELISA kit	R&D Systems	Cat# HS600C
Quantikine ELISA kit	R&D Systems	Cat# HS750
AllPrep DNA/RNA Mini Kit	Qiagen	Cat# 80204
ddPCR Supermix for probes (no dUTPS)	Bio-Rad Laboratories	Cat#186-3024
**Critical commercial assays**		
Abbott RealTime HIV-1 assay	Abbott	https://www.molecular.abbott/us/en/products/infectious-disease/realtime-hiv-1-viral-load
Roche COBAS TaqMan HIV-1 Test Version 2.0	Roche	https://diagnostics.roche.com/global/en/products/params/cobas-hiv-1-test.html
**Deposited data**		
Data S1 (Study protocol and amendments)	This manuscript	This manuscript
**Experimental models: Organisms/strains**		
Human participants	A5337 clinical trial participants	This manuscript
**Oligonucleotides**		
Proviral HIV DNA/RNA Forward Primer (5′-TACTGACGCTCTCGCACC)	Integrated DNA Technologies	https://pubmed.ncbi.nlm.nih.gov/18600229/
Proviral HIV DNA/RNA Reverse Primer (5′-TCTCGACGCAGGACTCG)	Integrated DNA Technologies	https://pubmed.ncbi.nlm.nih.gov/18600229/

(*Continued on next page*)

Continued

REAGENT or RESOURCE	SOURCE	IDENTIFIER
Proviral HIV DNA/RNA Probe (5′-FAM-CTCTCTCCTTCTAGCCTC)	Integrated DNA Technologies	https://pubmed.ncbi.nlm.nih.gov/18600229/
IPDAΨ F (5′-CAGGACTCGGCTTGCTGAAG)	Integrated DNA Technologies	https://pubmed.ncbi.nlm.nih.gov/30700913/
IPDAΨ Ψ R (5′-GCACCCATCTCTCTCCTTCTAGC)	Integrated DNA Technologies	https://pubmed.ncbi.nlm.nih.gov/30700913/
IPDAΨ Ψ Probe (5′-TTTTGGCGTACTCACCAGT)	Integrated DNA Technologies	https://pubmed.ncbi.nlm.nih.gov/30700913/ https://pubmed.ncbi.nlm.nih.gov/30700913/
IPDAΨ Env F (5′-AGTGGTGCAGAGAGAAAAAAGAGC)	Integrated DNA Technologies	https://pubmed.ncbi.nlm.nih.gov/30700913/
IPDAΨ Env R (5′-GTCTGGCCTGTACCGTCAGC)	Integrated DNA Technologies	https://pubmed.ncbi.nlm.nih.gov/30700913/
IPDAΨ Env intact probe (5′-CCTTGGGTTCTTGGGA)	Integrated DNA Technologies	https://pubmed.ncbi.nlm.nih.gov/30700913/
IPDAΨ Env hypermut (5′-CCTTAGGTTCTTAGGAGC)	Integrated DNA Technologies	https://pubmed.ncbi.nlm.nih.gov/30700913/
Software and algorithms		
SAS version 9.4	SAS Institute	N/A
FACSDiva software version 9.0	BD Biosciences	https://www.bdbiosciences.com/en-us/products/software/instrument-software/bd-facsdiva-software
R v. 4 (lmec version 1.0)	The R Project for Statistical Computing	https://www.r-project.org/

EXPERIMENTAL MODEL AND SUBJECT DETAILS

Study design

A5337 was a phase I/II, open-label, single-arm clinical trial to evaluate the effect of sirolimus on the HIV-reservoir size and immune function among well-suppressed individuals on antiretroviral therapy (ART). The target enrollment of this study was 30 participants ≥18 years of age and on a suppressive ART regimen (excluding PI-based or cobicistat-based regimens) for at least 24 months, with $CD4^+$ cell counts ≥350 cells/μL at study screening. Participants were to complete 20 weeks of oral sirolimus following a 12-week lead period and an additional 12 weeks follow-up period following cessation of the study drug. Because the aim of this pilot study was to investigate the biologic effects of sirolimus, the pre-defined, primary analyses were as-treated, limited to subjects who have data at baseline and week 32 (20 weeks on sirolimus) and who remained on study treatment and ART (and without virologic failure) through week 32.

Inclusion criteria

Additional inclusion criteria included plasma HIV-1 RNA below the level of quantitation for ≥24 months by an FDA-approved assay at any US laboratory that has a CLIA certification or its equivalent. Two plasma HIV-1 RNA measurements above the limit of quantification but <500 copies/mL in the 24 months prior to screening were allowed if directly preceded and followed by HIV-1 RNA below assay limit. Females of reproductive potential who were participating in sexual activity that could lead to pregnancy must have agreed to initiate effective contraceptives before sirolimus therapy, continued and maintained use for at least 12 weeks after sirolimus therapy has been stopped. The following laboratory criteria had to have been met: white blood cell (WBC) ≥3000 cells/μL, platelet count ≥125,000/mm^3, absolute neutrophil count >1300 cells/μL, aspartate aminotransferase (AST) < 1.25 x upper limit of normal (ULN), alanine aminotransferase (ALT) < 1.25 x ULN, calculated creatinine clearance (CrCl) ≥60 mL/min as estimated by the Cockcroft-Gault equation, fasting or non-fasting triglyceride level ≤350 mg/dL, fasting or non-fasting LDL <160 mg/dL, urine protein to urine creatinine ratio ≤1 g/g from random urine collection.

Exclusion criteria

Exclusion criteria included serious illness requiring systemic treatment and/or hospitalization within 30 days prior to study entry, AIDS-defining condition or oropharyngeal candidiasis within 90 days prior to study entry, intended modification to ART during the study, latent tuberculosis infection if prophylaxis had not been complete at least 48 weeks prior to study entry, active tuberculosis infection within 48 weeks of entry, history of current or active hepatitis B infection, HCV RNA+ within 90 days of study entry, history of a neoplastic disorder or clinically significant organ dysfunction prior to study entry, detectable Epstein-Barr virus in blood, any active infection requiring systemic antimicrobial therapy within 90 days of study entry, hypersensitivities to macrolide-like drugs or mTOR inhibitors, active drug or alcohol use or dependence vaccination within 14, breastfeeding, and anal or perianal administration of anti-HPV therapies 90 days prior to entry.

Discontinuation/Stopping criteria

Treatment was discontinued if any two consecutive $CD4^+$ cell counts <300 µL or >50% decreased from study entry value, any two consecutive HIV-1 RNA levels were >200 copies/mL, participant was repeatedly noncompliant missing >3 doses of study drug a week for 2 or more weeks, participant missed two consecutive PK monitoring blood draws, or if a participant missed two consecutive clinic visits. Participants were prematurely withdrawn from the study if ART was permanently discontinued or if the participant, primary care provider or study investigator felt that the participant should stop for any reason.

Sirolimus dosing and drug levels

Because of drug-drug interactions between sirolimus and ritonavir and cobicistat, participants on protease-inhibitor-based therapies were excluded from the study. For participants on an ART regimen that did not include a non-non-nucleoside reverse transcriptase inhibitor (NNRTI) regimen, and for those on rilpivirine (RPV) based regimen sirolimus was initiated at 0.025 mg/kg/day initial dose and 0.05 mg/kg/day. Dosing was adjusted based on trough sirolimus concentrations to achieve target concentrations between 5 and 10 ng/mL. Sirolimus levels were measured by FDA-approved clinical laboratory assays in Clinical Laboratory Improvement Amendments (CLIA) certified laboratories using liquid chromatography/tandem mass spectrometry to enable accurate and consistent measures of levels across study sites. The average sirolimus level was calculated for each participant using a weighted average to account for the differing numbers of trough measurements. Spearman correlations assessed associations between average trough level and virologic and immunologic changes.

Sample size estimation and biological sample allocations

Regarding the assessment of safety, the sample size of 30 sirolimus-treated participants would provide >90% probability of observing a sirolimus-related adverse event that would occur in 8% or more of treated individuals. Statistical power for identifying treatment effects on primary immunologic and virologic endpoints assumed 25 evaluable participants, on changes over 20 weeks of sirolimus treatment. Based on the paired t-test, power was estimated to be 80% to identify an effect size corresponding to a probability of 0.72 that a participant receiving sirolimus would have an observed increase in HIV-1 Gag-specific CD8 responses from pre- to post-treatment (probability 0.5 under the null hypothesis), and similarly a probability of 0.72 to have a decrease in cell-associated RNA. Power was estimated to be 80% to identify sirolimus effects corresponding to iSCA having probability of 0.75 to be below assay limit post-treatment, compared to probability 0.50 pre-treatment.

Human samples

Whole blood was collected at timepoints specific in the study protocol (Data S1) followed by plasma separation and isolation of human peripheral blood mononuclear cells (PBMCs). Plasma was frozen and stored at −80°C in a central biospecimen bank until further processing and testing. PBMCs were cryopreserved and stored in liquid nitrogen in a central biospecimen bank until further processing and testing.

Study approval

Institutional review boards from each participating ACTG site reviewed and approved the study documents, including informed consent forms. Informed consent was obtained from all participants in the study prior to participation.

METHOD DETAILS

HIV DNA and RNA quantification

HIV reservoir activity and size were assessed by the quantification of cell-associated HIV-1 RNA (CA-RNA) and DNA (CA-DNA).[80] Intracellular RNA and DNA were isolated from cryopreserved peripheral blood mononuclear cells (PBMCs) using the AllPrep DNA/RNA Mini Kit (Qiagen) as per manufacturer specifications with the addition of the optional spin column drying procedure prior to elusion in nuclease free water. 10 µL of genomic DNA, 12.5 µL of Universal Taqman mastermix (ABI), 0.75 µL of 10 µM forward primer (5′-TACTGACGCTCTCGCACC), 0.75 µL of 10 µM reverse primer (5′-TCTCGACGCAGGACTCG), and 1.0 µL of 5 µM FAM-MGB labeled probe (5′-FAM-CTCTCTCCTTCTAGCCTC) were added to each reaction well. HIV-1 standards were constructed by amplifying a cDNA region with primers that flank the region specified above from the HIV-1 reference strain HXB2 using the forward primer 5′-GGCTCACTATGCTGCCGCCC and the reverse primer 5′-TGACAAGCAGCGGCAGGACC. Cellular integrity for RNA analysis was assessed by the measurement of total extracted RNA and evaluation of the IPO-8 housekeeping gene.[81] Unspliced CA-RNA and total CA-DNA were quantified using a real-time PCR approach with primers/probes targeting conserved regions of HIV LTR/gag as previously described.[82] The IPDA was performed as described[41] at baseline (treatment week 0), treatment 4 and treatment week 20 in participants in the primary efficacy analysis. Primer and probe sequences for the PCR quantitation experiments were as follows: Ψ F (CAGGACTCGGCTTGCTGAAG), Ψ R (GCACCCATCTCTCTCCTTCTAGC), Ψ Probe (TTTTGGCGTACTCACCAGT), Env F (AGTGGTGCAGAGAGAAAAAAGAGC), Env R (GTCTGGCCTGTACCGTCAGC), Env intact probe (CCTTGGGTTCTTGGGA), Env hypermut (CCTTAGGTTCTTAGGAGC). ddPCR was performed on the Bio-Rad QX100 system using the ddPCR Supermix for probes (no dUTPS; Bio-Rad Laboratories). IPDA thermocycling was performed as follows: 1 cycle of enzyme activation at 95C for

10 min, 45 cycles of denaturing (94C)/annealing/extension (59C), 1 cycle of enzyme deactivation (98C) for 10 min and hold for 4-12C until ready for droplet reading. Droplet quantitation was performed using a QX100 ddPCR droplet reader (BioRad).

HIV single-copy assay

Plasma HIV residual viremia were measured using the validated ultrasensitive integrase single-copy assay (iSCA),[83] commonly used to assess levels of residual viral load.[42,64,80,84–87] Plasma from the participants was spiked with an internal Replication-Competent ASLV long terminal repeat with a Splice acceptor (RCAS) virion standard as a control for RNA extraction efficiency.[88] Real-time PCR reactions were performed with a Roche LightCycler 480 system using primers and probes specific to a conserved region of the HIV integrase gene.[83]

Plasma inflammation markers

Levels of soluble CD14 (sCD14), IL-6, IL7, and IP10 in EDTA plasma samples were measured using the Quantikine ELISA kits; Cat# DC140, HS600C, HS750 and DIP100 respectively, (all from R&D Systems) according to the manufacturer's instructions. Levels of D-dimers were measured using the Asserachrom D-DI immunoassay: cat#00947 (Diagnostica Stago, Asnieres France).

Flow cytometric analyses

Cryopreserved peripheral blood mononuclear cells (PBMCs) were thawed with RPMI1640 (Gibco) containing 10%FCS (Sigma) and 50U/mL benzonase (EMD Millipore). Cells were stained for 30 min at room temperature with LIVE/DEAD Fixable Aqua Dead Cell Stain Kit (Invitrogen) followed by cell-surface staining with anti-CD3 PerCP (SK7, BioLegend), anti-CD4 AF700 (RPA-T4, BD), anti-CD8 APC-Cy7 (SK1, BioLegend), anti-CCR7 PE-CF594 (150503, BD), anti-CD45RA BB515 (HI100, BD), anti-CD27 BV786 (O323, BioLegend), anti-CCR5 PE (2D7, BD), anti-PD-1 BV421 (EH12.1, BD), and anti-CD69 PE-Cy7 (FN50, BioLegend). Cells were then permeabilized with Foxp3/Transcription Factor Fixation/Permeabilization Concentrate and Diluent (eBioscience) for 30 min at 4°C and stained intracellularly for 45 min at 4°C with anti-Ki67 APC (Ki-67, BioLegend). Samples were acquired on a BD LSRII flow cytometer and analyzed with BD FACSDiva software.

In vitro stimulations

Cryopreserved PBMCs were thawed with RPMI1640 (Gibco) containing 10%FCS (Sigma) and 50U/mL benzonase (EMD Millipore) and then rested at 37°C in RPMI 1640 with10% FCS, 2mM L-glutamine (Gibco), and 50U/mL Pen Strep (Gibco) for approximately 6 h. Cells were then stimulated with 1 μg/mL HIV-1 Gag peptides, 1 μg/mL SEB (Toxin Technology Inc.), 10 μg/mL human CMV pp65 peptide pool, or a medium control along with Brefeldin A (BioLegend) and CD107α PE-CF594 (H4A3, BD) for 16 h at 37°C. The following reagents were obtained through the NIH AIDS Reagent Program, Division of AIDS, NIAID, NIH: HIV-1 PTE Gag Peptide Set (cat# 11554) and HCMV pp65 Peptide Pool (cat# 11549)[23,89,90]

Cells were stained with LIVE/DEAD Fixable Aqua Dead Cell Stain Kit (Invitrogen) followed by cell-surface staining with anti-CD3 BV711 (UCHT1, BD), anti-CD4 BUV395 (SK3, BD), and anti-CD8 APC-Cy7 (SK1, BioLegend). Cells were permeabilized with BD Cytofix/Cytoperm for 30 min at 4°C followed by intracellular staining for 45min at 4°C with anti-IFN-γ PE-Cy7 (B27, BioLegend), anti-TNF-α FITC (Mab11, BioLegend), anti-IL-2 APC (MQ1-17H12, BioLegend), anti-MIP-1β PE (D21-1351, BD), and anti-CD40L/CD154 (24–31, BioLegend). Samples were acquired on a BD LSRII flow cytometer and analyzed with FlowJo software. For each stimulant/marker combination on $CD4^+$ and $CD8^+$ T cells, there was an analogous unstimulated result measured by the same marker. This value was subtracted from the corresponding HIV GAG-, CMV- and SEB-stimulated result to yield the results used in analysis (setting to zero if subtracting the unstimulated result yielded a negative number).

QUANTIFICATION AND STATISTICAL ANALYSIS

HIV RNA and DNA

Statistical analyses are based on paired t-tests of each outcome, testing changes from baseline to treatment week 20 for the primary analysis population, and testing changes from baseline to treatment week 4 for the secondary analysis population. Results were $\log_{10}$-transformed prior to analyses. For the primary outcome of change in cell-associated HIV-1 RNA, a paired t-test also assessed changes between pre-treatment time points (treatment week −12 (study week 0) and treatment week 0 (study week 12)). Baseline is defined as the average of treatment weeks −12 and 0 (study weeks 0 and 12), or one of these time points if the other is missing. In the figures, both treatment weeks −12 and 0 are presented. To address left-censoring of CA-RNA, the analysis lower limit was determined from the normalized CA-RNA values as the largest assay lower limit across all results; results less than the analysis lower limit were imputed to half the analysis lower limit for analysis. This approach was also implemented for CA-DNA, iSCA and soluble biomarkers of inflammation. A supplemental analysis based on the left-censored normal distribution was also performed, left-censoring at the analysis lower limit.[91] No adjustments have been made regarding multiple comparisons.

Cell-associated HIV-1 RNA and DNA were normalized to copies/10^6 $CD4^+$ T cells by dividing by CD4% (divided by 100) from the same specimen date as the CA-RNA and CA-DNA sample. In cases where the CD4% was not available on the same date, CD4% from the closest date (before or after) was used. All statistical tests were 2-tailed, and 2-tailed confidence intervals are presented in the graphical results. Linear mixed effects models with random intercept, and fixed effects for the intercept and change from

baseline, were applied to pre-treatment and all 3 on-treatment time points (treatment wk4, wk12, wk20), and combining the primary and secondary analysis populations (i.e., n = 29 contributing to pre-treatment and wk4, n = 16 contributing to wk12 and wk20) to further explore the relationship between sirolimus use and HIV-1 persistence measures over time.

IPDA

To summarize IPDA changes (separately for changes to treatment week 4 and to treatment week 20), a descriptive approach was performed given the smaller evaluable sample size, excluding participants with no IPDA change (IPDA not detected at both time points). Longitudinal changes were estimated by the median change, derived from participant-specific changes of $\log_{10}$-transformed measures, and the proportion with decreases vs. increases. Participants whose intact proviral DNA levels went from detected to not detected were analyzed as the change with the lowest rank (greatest decreases). Participants whose intact proviral DNA levels went from not detected to detected were analyzed as the change with the highest rank (greatest increases).

All statistical and analysis codes used in analyses will be provided upon request. Data acquisition of clinical data at the sites was obtained prospectively during the study using the study case report form (CRFs), with data entry at each participating clinical site using standard ACTG procedures and trained personnel. Batch-tested laboratory data was generated at each testing lab and submitted securely (using Excel templates) to the ACTG's data management center, where it was loaded into INGRES tables after quality assurance review. All study data was then transferred securely to the statistical analysis center and converted to SAS datasets using validated programs. Statistical analyses were performed using SAS version 9.4. A supplemental analysis was performed in R using the lmec package.

Flow cytometric analyses

Longitudinal summaries are presented. Statistical analyses are based on 2-tailed paired t-tests for the secondary flow-based outcomes, testing changes from baseline to treatment week 20 for the primary analysis population, and testing changes from baseline to treatment week 4 for the secondary analysis population.

ADDITIONAL RESOURCES

ClinicalTrial.Gov registration number: NCT02440789 (https://clinicaltrials.gov/study/NCT02440789).

Cell Reports Medicine, Volume *5*

Supplemental information

Sirolimus reduces T cell cycling, immune checkpoint marker expression, and HIV-1 DNA in people with HIV

Timothy J. Henrich, Ronald J. Bosch, Catherine Godfrey, Hanna Mar, Apsara Nair, Michael Keefer, Carl Fichtenbaum, Daniela Moisi, Brian Clagett, Amanda M. Buck, Amelia N. Deitchman, Francesca Aweeka, Jonathan Z. Li, Daniel R. Kuritzkes, Michael M. Lederman, Priscilla Y. Hsue, Steven G. Deeks, and the ACTG A5337 Team

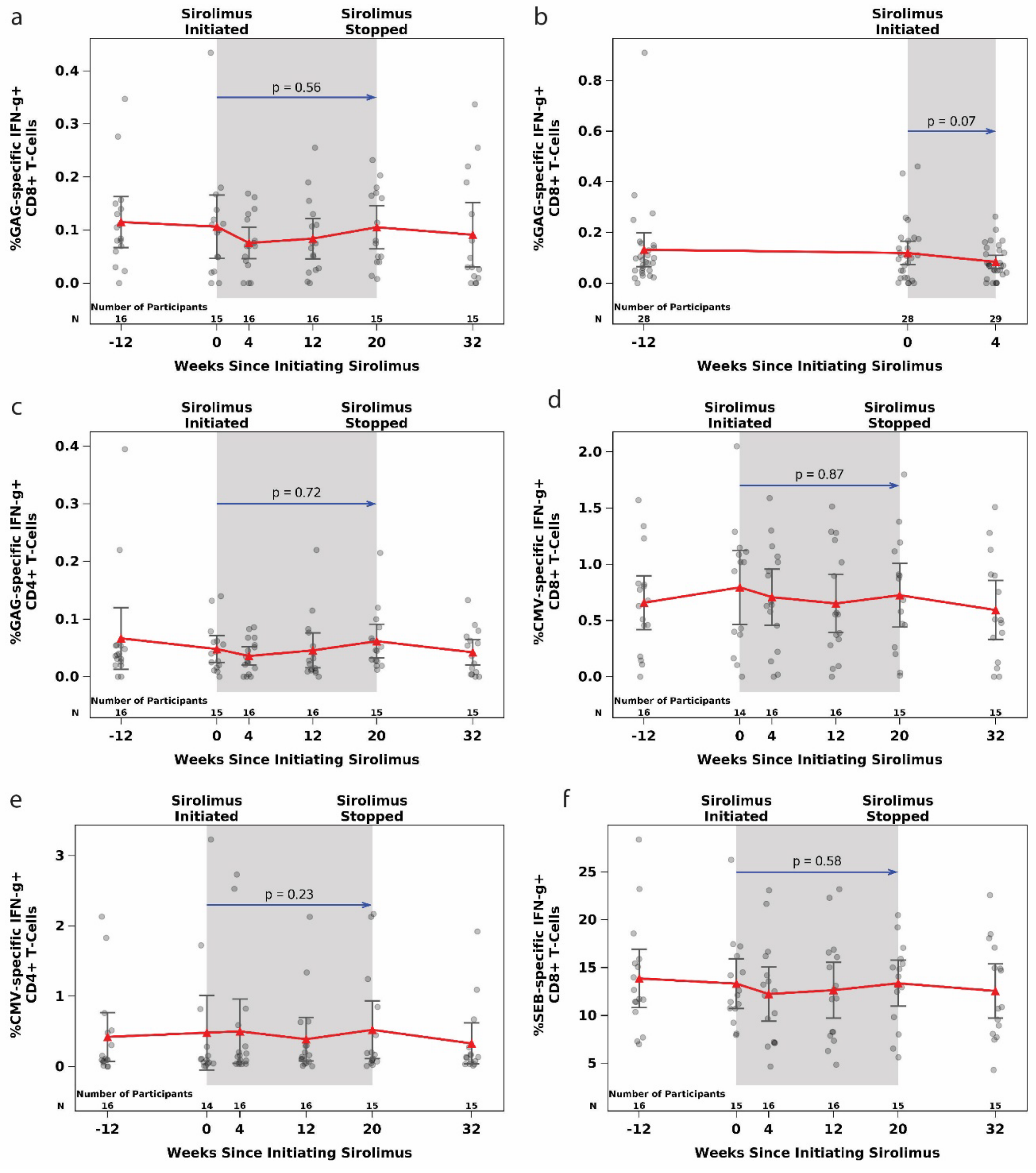

Supplementary Figure 1. Changes in the percent of antigen-specific T cell responses in response to sirolimus treatment, as related to Table 2. The percentages of CD8 cells producing IFNγ in response to HIV-1Gag exposure in the primary and secondary analysis populations are shown in (**a**) and (**b**), respectively. The percentage of CD4 T cells producing IFNγ in response to HIV-1 Gag exposure in the primary analysis population is shown in (**c**). Changes in the percentage of CD8 or CD4 cells producing IFNγ in response to CMV pp65 peptide and SEB are shown in (**d-f**) for the primary analysis population. No significant changes in CMV-specific T cell responses were observed from baseline to treatment week 20 in the primary efficacy population or from baseline to treatment week 4 in the secondary efficacy population. Similarly, there were no significant differences in SEB-specific T cell

responses between baseline and treatment week 20, although nominally significant decrease from baseline to treatment week 4 were seen in the percentages of IFNγ+ CD8 T-cells (mean change: -0.98%; P = 0.048), CD40L+ CD8 and CD4 T cells (mean change: -0.25% and -2.24%, respectively; P = 0.033, 0.02), IL-2+ CD8 and CD4 T cells (mean change: -0.70% and -1.32%, respectively; P = 0.001, 0.008) and TNFα+ CD4 T-cells (mean change: -1.23%; P = 0.011) 95% confidence intervals and means at each time point are presented. P values from two-tailed, paired T Tests.

Supplementary Table 1. Protocol-Defined Clinical Events and Toxicities and Reasons for Discontinuing Sirolimus as related to Figure 1.

	Total (N=32)
Treatment Not Started	2 (6%)
Completed < 20 Weeks of Sirolimus	14 (44%)
Disallowed Medication	1
Non-Protocol Defining Event (single CD4+T Cell count <300 c/mm^3)[a]	1
Non-Protocol Defined Low Grade Toxicities (Refusing Re-Challenge)	4
Not Able to Attend Clinic	1
Other (Inadvertently discontinued treatment early)	1
Protocol Defined Clinical Events/Toxicities	6
Confirmed CD4+T Cell Count <300 c/mm^3	1
Detectable EBV DNA in Blood	3
Grade 3 Stomatitis (possibly related)	1
Lipid Metabolism Disorder (possibly related)	1
Completed Full 20 Weeks of Sirolimus	16 (50%)

[a] Stopping criterion defined as confirmed CD4+T cell count <300 cells/μL or > 50% decline from pre-treatment baseline testing

Supplementary Table 2: Average Sirolimus Level and Number of Trough Measurements over the First 4 Weeks of Treatment for the Primary Efficacy, Secondary and Total Study Populations, as related to the Sirolimus and Drug Levels Methods Section.

		**Primary (N=16)**	**Secondary (N=13)**	**Total (N=29)**
Average Sirolimus Level (ng/mL)	Median	6.0	5.1	5.5
	Q1, Q3	5.3, 7.2	4.8, 6.1	5.0, 6.7
	10%, 90%	4.3, 8.6	3.2, 8.4	3.2, 8.6
	Min, Max	2.4, 8.6	2.6, 9.2	2.4, 9.2
Average Sirolimus Level < 5 ng/mL	No	13 (81%)	9 (69%)	22 (76%)
	Yes	3 (19%)	4 (31%)	7 (24%)
Number of Trough Sirolimus Levels Measured per Participant	Median	5	7	6
	Q1, Q3	4, 7	6, 8	4, 7
	10%, 90%	3, 8	5, 8	4, 8
	Min, Max	3, 8	4, 8	3, 8

Q1, Q3 = quartiles 1 and 3

Supplementary Table 3: Change in CA-DNA from Baseline to Treatment Weeks 4, 12, 20, 32 in the Primary efficacy population (n = 16) as related to Figure 2.

	Tx Week	N	Mean	Median	(Q1, Q3)	(Min, Max)	95% Confidence Interval	P-Value
**CA-DNA $\log_{10}$ c/10^6 CD4+ T-cells[a]**	4	16	-0.21	-0.08	(-0.34, 0.00)	(-1.44, 0.17)	(-0.42, 0.00)	0.05
	12	16	-0.17	-0.07	(-0.23, 0.08)	(-1.44, 0.31)	(-0.41, 0.06)	0.13
	20	16	-0.16	-0.13	(-0.31, -0.01)	(-0.59, 0.21)	(-0.27, -0.05)	**0.008**
	32	16	-0.15	-0.09	(-0.25, -0.01)	(-0.81, 0.12)	(-0.27, -0.02)	**0.028**

[a] Change is calculated as: Treatment Week - Baseline, Treatment Week 32 is 12 weeks after sirolimus discontinuation

Supplementary Table 4. Spearman Correlations: Change from Baseline to Sirolimus Treatment Week 20 in the Primary Efficacy Population as related to Table 2 and Supplementary Figure 1.

		Change in					
		log10 HIV DNA		log10 CA-RNA		CD4+ T-Cells	
**Change in**	**N**	**Spearman**	**P-Value**	**Spearman**	**P-Value**	**Spearman**	**P-Value**
% Ki67 CD4	16	-0.02	0.94	0.11	0.69	0.23	0.39
% Ki67 CD8	16	0.26	0.32	-0.22	0.42	-0.06	0.82
% PD1 CD8	16	0.00	1.00	0.20	0.45	-0.31	0.24
log10 IL-6	16	0.36	0.16	0.23	0.38	-0.33	0.22
log10 sCD14	16	-0.31	0.25	-0.09	0.75	0.34	0.20
log10 IP-10	16	0.01	0.96	0.25	0.34	-0.09	0.74
log10 D-Dimer	16	0.15	0.58	-0.10	0.71	-0.07	0.80
CD4+ T-Cells	16	0.02	0.93	0.08	0.78	1.00	.

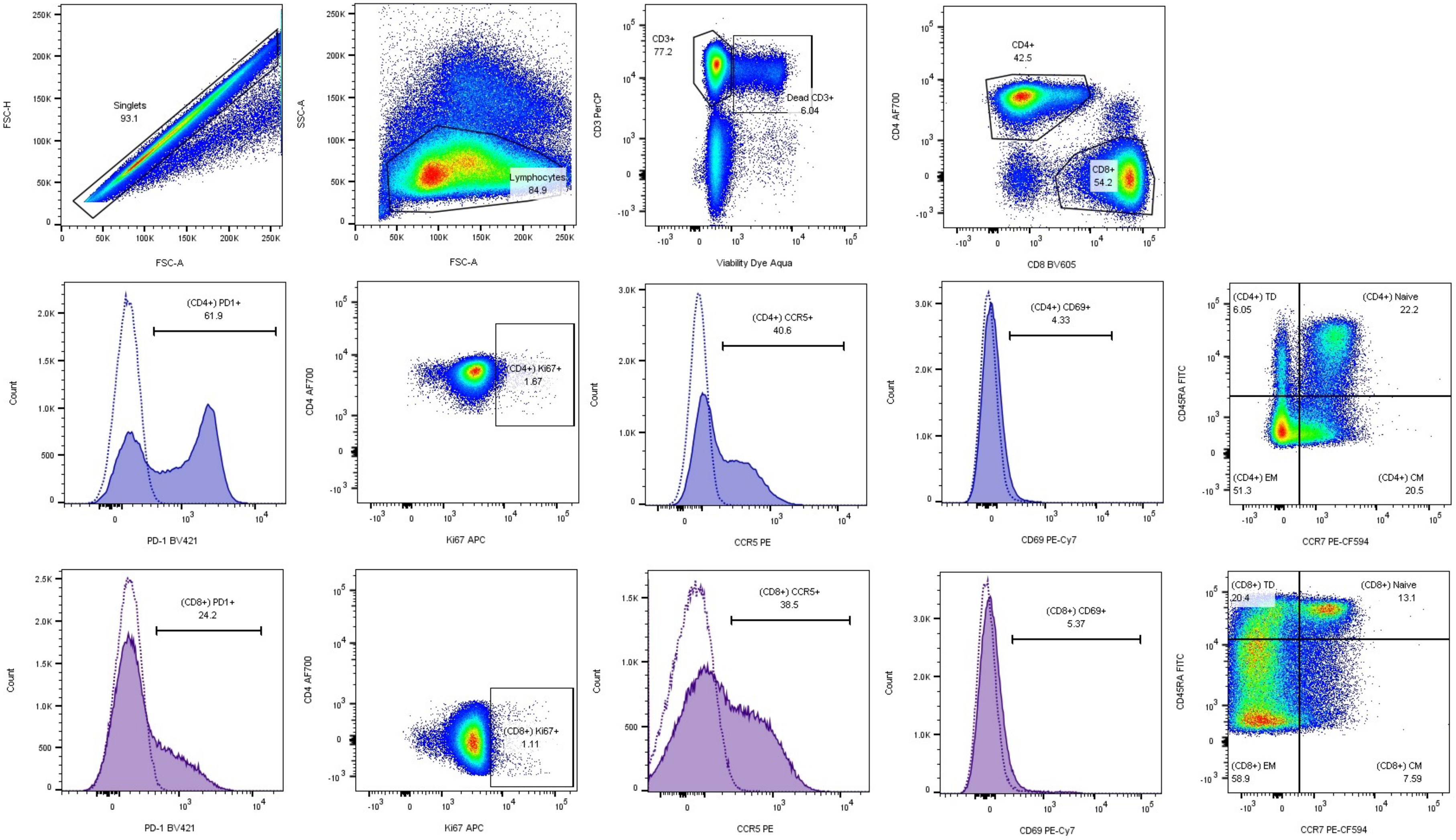

Supplementary Figure 2. Flow cytometric gating strategy for immune subset phenotyping, as related to the Flow Cytometric Analysis section of the Methods..

Data S1: ACTG A5337 protocol documents related to the STAR Methods study design section. These documents include the main protocol, protocol amendment, protocol amendment letter, and trial clarification letter.

A5337

Safety and Efficacy of Sirolimus for HIV Reservoir Reduction in Individuals on Suppressive Antiretroviral Therapy

A Limited Center Trial of the AIDS Clinical Trials Group (ACTG)

Sponsored by:

The National Institute of Allergy and Infectious Diseases

Industry Support Provided by:

ViiV Healthcare

**The ACTG HIV Reservoirs and Viral Eradication Transformative Science Group:**	**Joseph Eron, MD, Chair**
**Protocol Co-Chairs:**	**Timothy J. Henrich, MD**<br>**Priscilla Hsue, MD**
**Protocol Vice Chair:**	**Steven G. Deeks, MD**
**DAIDS Clinical Representative:**	**Catherine Godfrey, MD**
**Clinical Trials Specialist:**	**Elizabeth Hawkins, MA**

Version 2.0
July 18, 2016

ACTG
AIDS CLINICAL TRIALS GROUP

CONTENTS

Page

SITES PARTICIPATING IN THE STUDY 4
PROTOCOL TEAM ROSTER 5
STUDY MANAGEMENT 8
GLOSSARY OF PROTOCOL-SPECIFIC TERMS....10
SCHEMA 11

1.0 HYPOTHESES AND STUDY OBJECTIVES....14
1.1 Hypotheses....14
1.2 Primary Objectives....14
1.3 Secondary Objectives 14
1.4 Exploratory Objectives 14

2.0 INTRODUCTION....15
2.1 Background....15
2.2 Rationale 21

3.0 STUDY DESIGN 26

4.0 SELECTION AND ENROLLMENT OF **PARTICIPANTS** 27
4.1 Inclusion Criteria 27
4.2 Exclusion Criteria 29
4.3 Study Enrollment Procedures....31
4.4 Coenrollment Guidelines....32

5.0 STUDY TREATMENT 33
5.1 Regimens, Administration and Duration 33
5.2 Study Product Formulation and Preparation....34
5.3 Pharmacy: Product Supply, Distribution, and Accountability 35
5.4 Concomitant Medications....35

6.0 CLINICAL AND LABORATORY EVALUATIONS....37
6.1 Schedule of Events 37
6.2 Timing of Evaluations....40
6.3 Instructions for Evaluations 42

7.0 CLINICAL MANAGEMENT ISSUES....50
7.1 Toxicity 50
7.2 Management of Specific Toxicities....52
7.3 Management of HPV-Related Disease....53
7.4 Pregnancy Outcomes and Obstetrical History Reporting....53

8.0 CRITERIA FOR DISCONTINUATION 53
8.1 Permanent and Premature Treatment Discontinuation....53
8.2 Premature Study Discontinuation....54

CONTENTS (Cont'd)

Page

9.0 STATISTICAL CONSIDERATIONS....54
9.1 General Design Issues....54
9.2 Outcome Measures....54
9.3 Randomization and Stratification....56
9.4 Sample Size and Accrual....56
9.5 Monitoring....56
9.6 Analyses....57

10.0 PHARMACOLOGY PLAN....59
10.1 Pharmacology Study Design....59
10.2 Primary and Secondary Data, Modeling and Data Analysis....59

11.0 DATA COLLECTION AND MONITORING AND ADVERSE EVENT REPORTING....60
11.1 Records to Be Kept....60
11.2 Role of Data Management....60
11.3 Clinical Site Monitoring and Record Availability....60
11.4 Expedited Adverse Event Reporting to DAIDS....60

12.0 **PARTICIPANTS**....61
12.1 Institutional Review Board (IRB) Review and Informed Consent....61
12.2 **Participant** Confidentiality....62
12.3 Study Discontinuation....62

13.0 PUBLICATION OF RESEARCH FINDINGS....62

14.0 BIOHAZARD CONTAINMENT....62

15.0 REFERENCES....63

APPENDIX I: SAMPLE INFORMED CONSENT

SITES PARTICIPATING IN THE STUDY

A5337 is a limited center study open to select US clinical research sites (CRSs) that **ideally have** rapid sirolimus testing available within 72 hours via liquid chromatography/tandem mass spectrometry (LC tandem MS), **but laboratory results may be dependent on the testing laboratory's standard operating procedures. Refer to the Site tab on the protocol's webpage on the ACTG Member website for the list of eligible sites.**

PROTOCOL TEAM ROSTER

Co-Chairs
Timothy J. Henrich, MD
Assistant Professor of Medicine
Department of Experimental Medicine
University of California San Francisco
San Francisco General Hospital Campus, Building 3
San Francisco, CA
Phone: 415-206-5518
E-mail: timothy.henrich@ucsf.edu

Priscilla Hsue, MD
University of California, San Francisco
San Francisco General Hospital
Room 5G1
Cardiology SFGH
1001 Potrero Avenue
San Francisco, CA 94110
Phone: 415-206-8257
Fax: 415-206-5447
E-mail: phsue@medsfgh.ucsf.edu

Vice Chair
Steven G. Deeks, MD
UCSF, Positive Health Program
SFGH Building 80, Ward 84
996 Potrero Avenue
San Francisco, CA 94110
Phone: 415-476-4082 x330
Fax: 415-476-6953
E-mail: sdeeks@php.ucsf.edu

Virologist
Daniel R. Kuritzkes, MD
Division of Infectious Diseases
Brigham and Women's Hospital
65 Landsdowne St, Rm 449
Cambridge, MA 02139
Phone: 617-768-8398
Fax: 617-768-8738
E-mail: dkuritzkes@partners.org

Immunologist
Michael M. Lederman, MD
Case Western Reserve University
University Hospitals Cleveland
11100 Euclid Ave
Cleveland, OH 44106
Phone: 216-844-8786
Fax: 216-844-5523
E-mail:
lederman.michael@clevelandactu.org

DAIDS Clinical Representative
Catherine Godfrey, MD
TRP, DAIDS, NIAID, NIH, HIV Research Branch,
5601 Fishers Lane
Room 9E49
MSC 9830, Bethesda, MD 20852-9830
Phone: 240-627-3074
Fax: **301-**432-9282
E-mail: cgodfrey@niaid.nih.gov

Clinical Trials Specialist
Elizabeth Hawkins, MA
ACTG Network Coordinating Center
Social & Scientific Systems, Inc.
8757 Georgia Avenue, 12th Floor
Silver Spring, MD 20910-3714
Phone: 301-628-3335
Fax: 301-628-3302
E-mail: ehawkins@s-3.com

Senior Statistician
Ronald J. Bosch, PhD
Center for Biostatistics in AIDS Research
Harvard School of Public Health
651 Huntington Ave
Boston, MA 02115
Phone: 617-432-3024
Fax: 617-432-2843
E-mail: ronbosch@sdac.harvard.edu

PROTOCOL TEAM ROSTER (Cont'd)

Statistician
Justin Ritz, MS
Statistical and Data Analysis Center
Harvard School of Public Health
FXB Building, Room 510
Boston MA 02115
Phone: 617-432-3034
Fax: 617-432-3163
E-mail: jritz@sdac.harvard.edu

Data Manager
Apsara Nair, MS
Frontier Science and Technology
Research Foundation
4033 Maple Road
Amherst, NY 14226
Phone: 716-834-0900 x7293
Fax: 716-834-8432
E-mail: nair@fstrf.org

DAIDS Pharmacist
Nayri Khairalla, BS, PharmD
Pharmaceutical Affairs Branch
OCSO, DAIDS, NIAID, NIH
5601 Fishers Lane
Room 9D46
Rockville, MD 20852
Phone: 301-761-6659
Fax: 240-627-3112
E-mail: Nayri.khairalla@nih.gov.

Pharmacologist
Francesca Aweeka, PharmD
Clinical Pharmacy
University of California, San Francisco
P.O. Box 0622, C152
521 Parnassus Avenue
San Francisco, CA 94143-0622
Phone: 415-476-0339
Fax: 415-476-0307
E-mail: faweeka@sfghsom.ucsf.edu

Investigators
Corey S. Cutler MD, MPH
Department of Medical Oncology
Dana-Farber Cancer Institute
450 Brookline Avenue
Boston, MA 02115
Phone: 617-632-5946
Fax: 617-632-5168
E-mail: corey_cutler@dfci.harvard.edu

Sulggi A. Lee, MD PhD
University of California, San Francisco
San Francisco General Hospital
995 Potrero Avenue, Bldg 80, Box 0874
San Francisco, CA 94110
Phone: 415-735-5127
Fax: 415-476-6953
E-mail: sulggi.lee@ucsf.edu

Rafick Sekaly, PhD
Vaccine Gene Therapy Institute-Florida
VGTI-FL, 9801 SW Discovery Way
Port St. Lucie, FL 34987
Phone: 772-345-5668
Fax: 772-345-5752
E-mail: rpsekaly@vgti-fl.org

Field Representatives
Susan Pedersen, RN, BSN
The UNC Global HIV Prevention and
Treatment Clinical Trials Unit
130 Mason Farm Road,
Campus Box 7215
Chapel Hill, NC 27599
Phone: 919-966-6713
Fax: 919-966-8928
E-mail: spederse@med.unc.edu

Becky Straub, RN, BSN, MPH
The UNC Global HIV Prevention and
Treatment Clinical Trials Unit
130 Mason Farm Road
Campus Box 7215
Chapel Hill, NC 27599
Phone: 919-843-9975
Fax: 919-966-8928
E-mail: bstraub@med.unc.edu

Laboratory Technologist
David L. Shugarts, MA
Infectious Diseases Clinical Trials Lab
University of Colorado Denver
12700 E. 19th Ave. Room 11006
Bldg. P15 RC-2
Aurora, CO 80045
Phone: 303-724-2981
Fax: 303-724-2985
E-mail: david.shugarts@ucdenver.edu

Community Scientific Subcommittee (CSS) Representatives
Danielle Campbell, MPHc
University of California, Los Angeles CARE Center
8810 S. Mary Avenue
Los Angeles, CA 90002
Phone: 310-910-8341
E-mail: daniellecampbell@cdrewu.edu

Michael Dorosh, MA
University of Colorado
38 West Maple Avenue
Denver, CO 80223-1839
Phone: 303-777-5737
Fax: 720-920-7110
E-mail: michael@ontheten.org

Laboratory Data Manager
Christopher Hensel
Frontier Science and Technology Research Foundation
4033 Maple Road
Amherst, NY 14226-1056
Phone: 716-834-0900
Fax: 716-833-0655
E-mail: chensel@fstrf.org

Industry Representatives
Belinda Ha, PhD
ViiV Healthcare
Five Moore Drive
Research Triangle Park, NC 27709
Phone: 919-483-8284
E-mail: belinda.f.ha@viivhealthcare.com

Andrew R. Zolopa, MD
ViiV Healthcare
Five Moore Drive
Research Triangle Park, NC 27709-3398
Phone: 919-315-8183
E-mail: Andrew.r.zolopa@viivhealthcare.com

STUDY MANAGEMENT

All questions concerning this protocol should be sent to actg.corea5337@fstrf.org via e-mail. The appropriate team member will respond with a "cc" to actg.corea5337@fstrf.org. A response should generally be received within 24 hours (Monday-Friday).

Protocol E-mail Group

Sites should contact the **User** Support Group at the Data Management Center (DMC) as soon as possible to have the relevant personnel at the site added to the 'prota5337' e-mail group. Include the protocol number in the e-mail subject line.

- Send an e-mail message to actg.user.support@fstrf.org.

Clinical Management

For questions concerning entry criteria, toxicity management, concomitant medications, and coenrollment, contact the protocol team.

- Send an e-mail message to actg.corea5337@fstrf.org. Include the protocol number, patient identification number (PID), and a brief relevant history.

Laboratory

For questions specifically related to immunology laboratory tests, contact the protocol immunologist.

- Send an e-mail message to actg.corea5337@fstrf.org (ATTN: Michael Lederman, MD).

For questions specifically related to virology laboratory tests, contact the protocol virologist.

- Send an e-mail message to actg.corea5337@fstrf.org (ATTN: Daniel Kuritzkes, MD).

For questions specifically related to pharmacology laboratory tests, contact the protocol pharmacologist.

- Send an e-mail message to actg.corea5337@fstrf.org (ATTN: Francesca Aweeka, PharmD).

Data Management

- **For nonclinical questions about transfers, inclusion/exclusion criteria, case report forms (CRFs), randomization/registration, and other data management issues, contact the data manager. CRFs completion guidelines and participant completed CRFs can be downloaded from the FSTRF website at www.fstrf.org**For transfers, reference the **Study Participant** Transfer SOP 119, and contact Apsara Nair, MS directly.
- For other questions, send an e-mail message to actg.corea5337@fstrf.org (ATTN: Apsara Nair, MS).
- Include the protocol number, PID, and a detailed question.

Participant Registration

For **participant** registration questions or problems and study identification number (SID) lists. Send an e-mail message to rando.support@fstrf.org. Call the Statistical and Data Analysis Center (SDAC)/DMC Randomization Desk at 716-834-0900 extension 7301.

Computer and Screen Problems

Contact the SDAC/DMC programmers.

- Send an e-mail message to actg.support@fstrf.org or call 716-834-0900 x7302.

Protocol Document Questions
For questions concerning the protocol document, contact the clinical trials specialist.
- Send an e-mail message to actg.corea5337@fstrf.org (ATTN: Elizabeth Hawkins, MA).

Copies of the Protocol
To request a hard copy of the protocol, send an e-mail message to ACTGNCC@s-3.com (ATTN: Diane Delgado). Electronic copies can be downloaded from the ACTG Web site (https://www.actgnetwork.org).

Product Package Inserts and/or Investigator Brochures
To request copies of product package inserts or investigator brochures, contact the DAIDS Regulatory Support Center (RSC) at RIC@tech-res.com or call 301-897-1708.

Protocol Registration
For protocol registration questions, send an e-mail message to Protocol@tech-res.com or call 301-897-1707.

Protocol Activation
For questions related to protocol activation, contact the Elizabeth Hawkins at ehawkins@s-3.com or ACTG Site Coordination group at actgsitecoordination@s-3.com.

Study Product
For questions or problems regarding study product, dose, supplies, records, and returns, call Nayri Khairalla, BS, PharmD, protocol pharmacist, at **301-761-6659.**

Study Drug Orders
Call the Clinical Research Products Management Center (CRPMC) at 301-294-0741.

Expedited Adverse Event (EAE) Reporting/Questions
Contact DAIDS through the RSC Safety Office at DAIDSRSCSafetyOffice@tech-res.com or call 1-800-537-9979 or 301-897-1709; or fax 1-800-275-7619 or 301-897-1710.

Phone Calls
Sites are responsible for documenting any phone calls made to A5337 team members.
- Send an e-mail to actg.corea5337@fstrf.org.

Protocol-Specific Web Page
Additional information about management of the protocol can be found on the protocol-specific web page (PSWP).

GLOSSARY OF PROTOCOL-SPECIFIC TERMS

5FU	Fluorouracil
AUC	area under the curve
BCG	Bacillus Calmette-Guerin
BOOP	bronchiolitis obliterans organizing pneumonia
CCR5	C-C chemokine receptor type 5
C_{max}	maximum concentration
CM	central memory
CMV	cytomegalovirus
EBV	Epstein-Barr virus
E/CIA	chemiluminescence immunoassay
EM	effector memory
eGFR	estimated glomerular filtration rate
**HHV**	**human herpes virus**
HSCT	hematopoietic stem cell transplantation
IFN	interferon
IL-2	interleukin 2
KS	Kaposi sarcoma
MHC	major histocompatibility complex
mTOR	mammalian target of rapamycin
PD-1	programmed cell death protein 1
PK	pharmacokinetic
SCA	single-copy assay
SD	standard deviation
SMC	Study Monitoring Committee
TCM	central memory T cells
TM	transitional memory
T_{max}	time to peak concentration
TST	tuberculin skin test

SCHEMA

A5337

Safety and Efficacy of Sirolimus for HIV Reservoir Reduction in Individuals on Suppressive Antiretroviral Therapy

DESIGN	A5337 is a phase I/II, open label, single arm, pilot study to evaluate the safety of sirolimus and its efficacy with respect to its effects on HIV-1 reservoir size and immune function. This study will assess the effects of sirolimus on HIV-1-specific CD8+ T-cell function, HIV transcription, and residual viral production. Measurement of additional inflammatory markers, immunological studies, and reservoir size will be performed on stored specimens if the initial findings appear promising.
DURATION	44 weeks (12-week pre-sirolimus treatment lead-in period, followed by 20 weeks of sirolimus treatment and an additional 12 weeks off sirolimus treatment).
SAMPLE SIZE	30 **participants**
POPULATION	HIV-infected men and women ≥18 years of age, maintained on suppressive antiretroviral therapy (ART) for ≥24 months with CD4+ cell count ≥**350** cells/mm^3. **Participants** may not be on a PI-based or cobicistat-based regimen 3 months prior to and at any time after study entry and must remain on ART during sirolimus study treatment.
STRATIFICATION	By class of antiretroviral (ARV) regimen
REGIMEN	▪ For **participants** on a non-protease inhibitor (PI), non-non-nucleoside reverse transcriptase inhibitor (NNRTI) regimen, and for those on a non-PI, rilpivirine (RPV) based regimen:<br><br>Sirolimus 0.025 mg/kg/day initial dose for 20 weeks<br><br>▪For **participants** who are on an NNRTI regimen with the exception of RPV:<br><br>Sirolimus 0.05 mg/kg/day initial dose for 20 weeks.<br><br>Dosing will be adjusted based on trough sirolimus concentrations to achieve target concentrations between 5 and 10 ng/mL.

Study Design Schematic

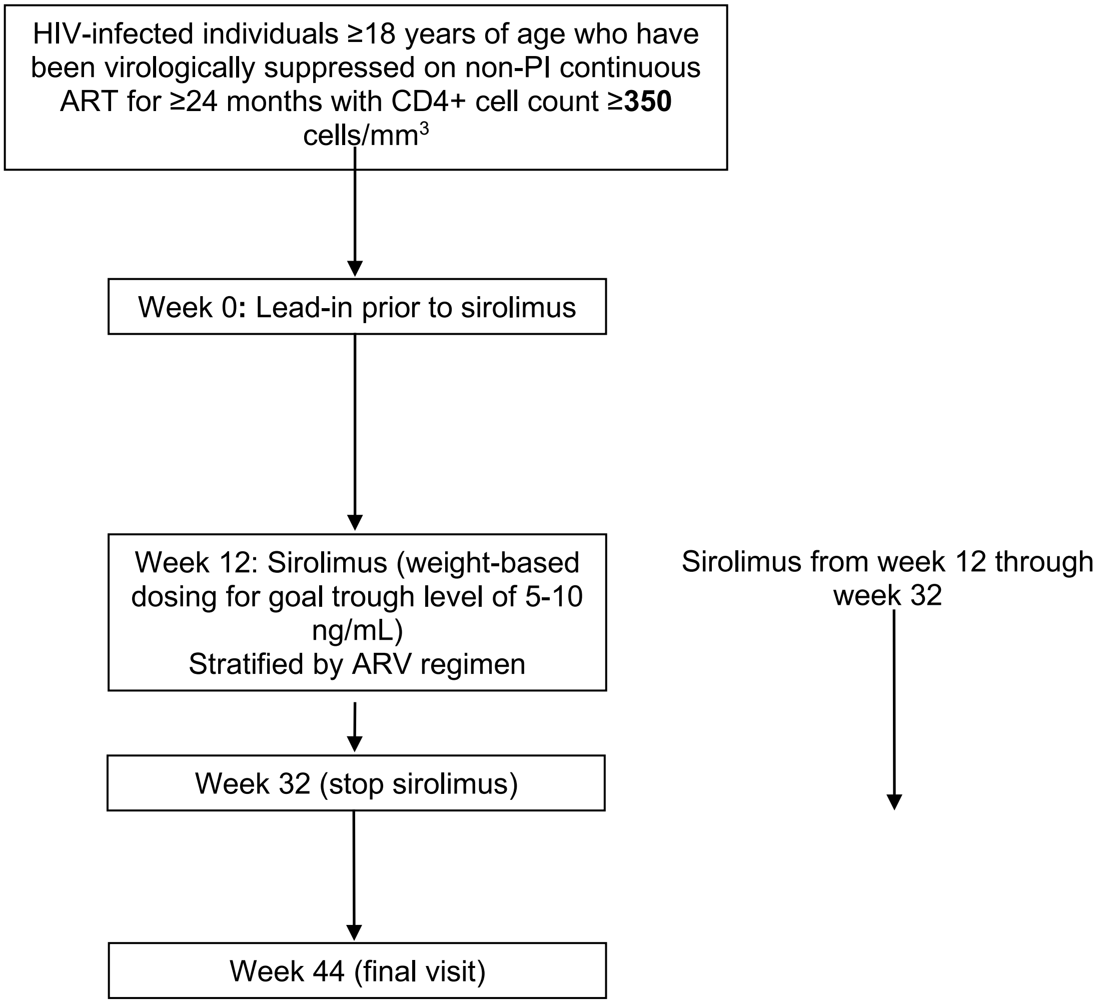

1.0 HYPOTHESES AND STUDY OBJECTIVES

1.1 Hypotheses

1.1.1 Sirolimus will be safe and tolerated in **participants** on non-protease inhibitor (PI) suppressive antiretroviral therapy (ART).

1.1.2 Sirolimus will improve HIV-specific cellular immunity and reduce T-cell activation, which in turn will cause decreases in viral transcription/cell-associated RNA levels and levels of low-level residual viremia.

1.1.3 Sirolimus will cause down-regulation of programmed cell death protein (PD-1) and suppression of homeostatic proliferation of the latent reservoir, leading to reductions in cell-associated HIV-1 DNA levels.

1.2 Primary Objectives

1.2.1 To assess the safety of sirolimus in HIV-infected **participants** receiving non PI-based suppressive ART.

1.2.2 To evaluate changes in the frequency (%) of HIV-1 Gag-specific CD8+ cells after 20 weeks of sirolimus therapy.

1.2.3 To assess changes in CD4+ cell-associated HIV-1 RNA and plasma HIV-1 RNA by single-copy assay (SCA) at baseline and at week 20 of sirolimus therapy.

1.3 Secondary Objectives

1.3.1 To assess the impact of sirolimus on measures of reservoir size including HIV-1 DNA levels in CD4+ cells and RNA levels by conventional assay.

1.3.2 To assess the impact of sirolimus on CD4+ cell counts.

1.3.3 To assess the impact of sirolimus on HIV-1-specific CD4+ cell responses and HIV-1-specific CD8+ cell responses.

1.3.4 To assess the impact of sirolimus on T-cell activation and proliferation (% CD4+ and CD8+ cells CD38+/HLA-DR+, CD25+, PD-1+, Ki67+ and PD-L1 expression).

1.4 Exploratory Objectives

1.4.1 To assess the pharmacokinetic (PK) interactions between sirolimus therapy and antiretroviral (ARV) drug levels.

1.4.2 To perform ex vivo flow cytometric phenotyping of activation/proliferation in CD4+ and CD8+ central memory (CM), transitional memory (TM), effector memory (EM) and naïve populations as defined by CCR7 and CD27 expression.

1.4.3 To measure the effects of sirolimus on homeostatic proliferation, **gene expression and transcriptional regulation.**

1.4.4 To assess the impact of sirolimus on soluble markers of inflammation, including IL-6, IL-7 and IL-15.

1.4.5 To measure the effects of sirolimus on HIV-1-specific antibody levels, the breadth of antibody responses, and B cell phenotype, rearrangements, and class switching.

1.4.6 To assess the relationship between human papillomavirus (HPV) DNA levels and HPV-related disease from anal swabs and anal Pap smears prior to and at completion of sirolimus therapy, and to assess the relationship between HPV and immune activation and reservoir size.

1.4.7 To evaluate whether the use of sirolimus is associated with more frequent reactivation or suppression of human herpes viruses (i.e., cytomegalovirus [CMV], Epstein Barr virus [EBV], herpes simplex viruses [HSV] and human herpes viruses [HHV] 6, 7, and 8) as measured in longitudinally collected oral swabs.

2.0 INTRODUCTION

2.1 Background

Despite complete or near complete inhibition of viral replication with standard therapies, replication-competent HIV persists indefinitely in all individuals. Chronic inflammation and/or immune dysfunction also persist during effective therapy [1]. Theoretically, host immune environment may contribute to HIV persistence through at least five independent mechanisms: (a) activating viral replication in latently-infected T cells (leading to HIV replication, assuming ART is not fully effective), (b) increasing the availability of susceptible target cells (making de novo infection more likely), (c) inhibiting the function of the adaptive immune system, (d) stimulating directly or indirectly the proliferation and expansion of memory T cells harboring replication competent HIV, and/or (e) stimulating intracellular signaling pathways (eg, PD-1) that may cause and maintain latent infection. The potential role of immune-based therapeutics as a component of a curative strategy is now recognized as a key research question in the field[1].

This study evaluates an intervention that may have an impact on HIV viral reservoirs, which has implications for viral eradication and ultimately ART-free remission of HIV. To date, few interventions have led to significant reductions in the size of the cellular HIV-1 reservoir. In addition, this study investigates the viral and immune pathogenesis of HIV-1 infection and related complications. The pathogenesis/immunology-oriented approach in this study of sirolimus treatment in HIV patients will help to identify underlying mechanisms of mammalian target of rapamycin (mTOR) inhibition, including the potential role of inflammation and immune activation, linking them to HIV disease

characteristics and reservoir size. In addition, A5337 may potentially provide important infrastructure by which to test the effects of using mTOR inhibition to target HIV viral reservoirs. Data obtained from this study will provide critical insights into a number of fundamental questions regarding the pathogenesis of long-term antiretroviral-treated HIV disease. Although the primary objective is to prepare for future clinical trials aimed at HIV-1 reservoir reduction using sirolimus and other drugs, the knowledge gained from this study could have broader implications, including the potential effects of anti-inflammatory agents in preventing inflammation-associated non-AIDS events. The results from this pilot study will be used to generate preliminary data for future grants and later phase clinical trials.

2.1.1 Sirolimus, T-cell activation and immunomodulatory effects

Sirolimus (rapamycin) is a naturally occurring macrolide antibiotic that inhibits mTOR, a key regulatory kinase that controls cell-cycle progression [2] and has complex effects on T-cell activation and function [3]. As a consequence, progression from G1 to S phase is blocked in activated T cells. In contrast to other immune suppressing agents such as cyclosporine, the inhibitory effect of sirolimus on cell-cycle progression appears to be limited to T cells that are activated by cytokines and does not occur in T cells activated directly by antigen-TCR engagement [4, 5]. Sirolimus has also been shown to inhibit class switching in B cells and promote cross-strain protection when used concomitantly with influenza vaccination [6].

2.1.2 Clinical use of sirolimus therapy

Sirolimus is approved for the prophylaxis of organ rejection in patients aged 13 years and older receiving renal transplants [7, 8]. However, sirolimus is used in other settings such as prophylaxis of graft-versus-host-disease after allogeneic hematopoietic stem cell transplantation (HSCT) [9-13] and as a treatment for Kaposi sarcoma (KS) in HIV-infected patients as detailed below and in solid-organ transplant patients [14-16]. Sirolimus is often used in combination with other immunosuppressive medications, such as cyclosporine or calcineurin inhibitors, and as a result, a large proportion of safety and efficacy data are in the setting of concomitant therapies or in populations with significant underlying morbidity.

2.1.3 Sirolimus use in the setting of HIV and hepatitis C virus (HCV) infections

Sirolimus may have utility in the management of HIV infection [17]. Reductions in HIV-associated T-cell activation should lead to a reduction in HIV replication, as has been observed with other immunosuppressant drugs. Sirolimus also can reduce HIV replication in vitro, possibly due to its impact in modulating the pro-inflammatory aspects of interleukin 2 (IL-2) on cytokine stimulated T cells [18] and/or by reducing HIV transcription [19]. Sirolimus reduces C-C chemokine receptor type 5 (CCR5) expression on T cells, which may make them less susceptible to HIV infection [20-22]. Sirolimus may also attenuate the increased T-cell cycling seen in untreated HIV infection [23] and in persons with immunologic failure on suppressive ART [24].

In a recent pilot study involving seven HIV-infected adults with KS, sirolimus was well tolerated and resulted in partial KS remissions in three individuals [14]. The drug has also been used in transplantation studies. No increased risk of opportunistic infections has been noted in HIV-infected allogeneic HSCT recipients receiving sirolimus for prevention of graft-versus-host disease [25]. Among 150 HIV-infected individuals receiving solid organ transplant [26], our group has shown that sirolimus exposure was safe.

A small report of the effects of sirolimus treatment in HIV-infected liver transplant recipients showed significantly better control of both HIV and HCV replication in the patients receiving sirolimus monotherapy, with two of six patients showing complete clearance of HCV [15]. Complete HCV clearance upon conversion to sirolimus therapy has also been described in HIV-infected patients undergoing liver transplant [27].

2.1.4 Sirolimus and cytomegalovirus (CMV) and HPV infection

A comprehensive review of the literature of CMV coinfection in renal transplant recipients shows that sirolimus and everolimus (a sirolimus derivative) may decrease the incidence and severity of clinical CMV disease [28]. A recent report showed a significant increase in (CMV-specific) effector-type CD8+ and CD4+ cell counts in patients undergoing everolimus dual therapy, which may partially explain the reported low incidence of CMV-related pathology in everolimus-treated patients [29].

A relationship between the mTOR pathway and HPV-related disease has been described, and promising in vitro studies suggest a role for future therapeutic efforts for HPV-associated tumors via inhibition of the E6 and E7 oncoprotein [30]. Early clinical studies suggest that inhibition of this pathway induces in vitro regression of cutaneous warts and may play a role in the therapy of cervical cancer and HPV-associated head and neck cancers [31, 32]. Reduction of persistent HPV infection may reduce the HPV-related burden of disease in the anogenital tract. Cutaneous warts have also been shown to resolve in the setting of conversion from tacrolimus or tacrolimus and cyclosporine to sirolimus in a transplant patient.

Oncogenic HPV, in particular HPV 16, induces a local inflammatory response as evidenced by increased isolation of soluble markers of inflammation [33]. There are no data on whether this local response contributes to the systemic inflammatory response. It is plausible, however, that this local inflammation may contribute to an increased HIV-1 reservoir size.

As part of this study we will evaluate whether the use of sirolimus is associated with more frequent reactivation or suppression of HHV as measured in longitudinally-collected oral swabs. Shedding of HHV DNA is frequently detected in saliva of healthy individuals and more frequently in HIV-infected adults and persistent replication of HHV (in particular CMV) has been repeatedly associated with increased systemic inflammation and cellular activation. As an alternative hypothesis, since persistent

inflammation can also be a trigger for HHV reactivation, reduction in inflammation and improvement of effector immune responses after sirolimus administration might be associated with decrease in HHV replication as seen with clinical KS.

Sirolimus may be associated with suppression of CMV due to two potential factors: 1) increased CMV-specific effector immune responses as detailed in study rationale which may explain the low incidence of CMV-related pathology in mTOR inhibitor-treated participants, and 2) reduction in oral and systemic cytokine-driven immune activation and inflammation leading to lower levels of oral or genital CMV secretion. Given an existing body of literature showing potential benefits of mTOR inhibition on CMV infection, we have extrapolated from this information to hypothesize that there will be less CMV shedding from both oral and genital tissues in the setting of sirolimus use. Alternatively, CMV shedding may increase due to immune suppressive effects of sirolimus.

2.1.5 Safety of sirolimus in HIV-uninfected patients

The boxed warning in the FDA sirolimus labeling has been updated to state that increased susceptibility to infection and the possible development of lymphoma and other malignancies may result from immunosuppression [7]. Although other cancers such as non-melanoma skin cancers were previously thought to be associated with sirolimus, the current body of evidence suggests that this is no longer the case. Overall, current observations suggest that approved use of sirolimus in organ transplant recipients may reduce the burden or recurrence of non-melanoma skin cancers. There is a paucity of data on the use of sirolimus as non-melanoma skin cancer prophylaxis in non-transplant populations, but there is growing evidence that mTOR inhibitors may be effective in treating other solid tumors of the skin, such as KS [34-36].

Serious adverse events (AEs) in populations treated with sirolimus monotherapy for various solid tumors were primarily limited to hyperglycemia, gastrointestinal disease (including apthous ulcers, mucositis, and diarrhea), infection (including pneumonia, cellulitis, urinary tract infections), dyspnea and anemia [15, **37, 38**]. Although minor AEs of this nature have been noted in a majority of subjects taking sirolimus or related drugs as monotherapy [**38**], the high prevalence of AEs were likely due to the lack of drug level monitoring. In our clinical experience, the prevalence of AEs is lower in clinical practice settings where drug levels are monitored, although it is unclear if concomitant immunosuppressive medications may contribute to these findings through additive effects.

AEs associated with sirolimus use in the setting of transplantation include [7]:

- Increased susceptibility to infection, including activation of latent Epstein-Barr viral infection, and the possible development of lymphoma, as discussed above.

- Hypersensitivity reactions including anaphylactic/anaphylactoid reactions, angioedema, exfoliative dermatitis, and hypersensitivity vasculitis have been associated with the administration of sirolimus but are rare. The concomitant use of sirolimus with other drugs known to cause angioedema, such as ACE-inhibitors, may increase the risk of developing angioedema.
- Impaired or delayed wound healing in patients receiving sirolimus, including lymphocele and wound dehiscence, has been reported.
- Proteinuria.
- Fluid accumulation, including peripheral edema, lymphedema, pleural effusion, ascites, and pericardial effusions (including hemodynamically significant effusions and tamponade requiring intervention in children and adults) in patients receiving sirolimus.
- Increased serum cholesterol and triglycerides requiring treatment occurred more frequently in patients treated with sirolimus compared to placebo controls.
- Interstitial lung disease (including pneumonitis, bronchiolitis obliterans organizing pneumonia [BOOP] and pulmonary fibrosis) with no identified infectious etiology have occurred in patients receiving immunosuppressive regimens including sirolimus. In some cases, the interstitial lung disease has resolved upon discontinuation or dose reduction of sirolimus and the risk may be increased as the trough sirolimus concentration increases. Underlying lung disease may increase risks of pulmonary toxicity in lung transplant patients.

2.1.6 Sirolimus and pregnancy

Sirolimus is pregnancy Category C [7]. Sirolimus is embryo/fetotoxic in rats when given in doses approximately 0.2 to 0.5 times the human doses (adjusted for body surface area). Embryo/fetotoxicity was manifested as mortality and reduced fetal weights but no teratogenesis was evident. There are no adequate and well-controlled studies in pregnant women.

2.1.7 Sirolimus and fertility

Sirolimus plays a central inhibitory role in a stem cell factor (SCF)/c-kit-dependent process in spermatogonia via the phos3phoinositide3-kinase (PI3-K)/(AKT)/p70S6K pathway and has been shown to have effects on both male and female fertility **[39].** Testosterone has been shown to decrease during sirolimus use whereas follicle stimulating hormone and luteinizing hormone increase **[40].** Furthermore, six patients (four men and two women), all of whom had either fathered or conceived children and who were receiving sirolimus for rejection prophylaxis pre-kidney transplant, developed gonadal dysfunction and infertility on average 5-12 months after transplantation. Sirolimus was discontinued, and all four male patients demonstrated full recovery of the oligo/azoospermia and restoration of fertility. Both women developed amenorrhea during therapy; amenorrhea resolved in only one patient after discontinuation of therapy [**41].** Transient oligospermia or azoospermia have also been reported in two other male patients in the setting of sirolimus use for renal transplantation **[42,43].**

2.1.8 Sirolimus pharmacokinetics and pharmacodynamics

Sirolimus is a substrate for both CYP3A4 and P-gp. Sirolimus is extensively metabolized in the intestinal wall and liver and undergoes counter-transport from enterocytes of the small intestine into the gut lumen. Inhibitors of CYP3A4 and P-gp increase sirolimus concentrations. Inducers of CYP3A4 and P-gp decrease sirolimus concentrations. After a single dose of (C14) sirolimus oral solution in healthy volunteers, the majority (91%) of radioactivity was recovered from the feces, and only a minor amount (2.2%) was excreted in urine. The mean ±SD terminal elimination half-life (t½) of sirolimus after multiple dosing in stable renal transplant patients was estimated to be about 62 ±16 hours. Following administration of rapamune oral solution, the mean times to peak concentration (t_{max}) of sirolimus are approximately 1 hour and 2 hours in healthy subjects and renal transplant patients, respectively [7]. The systemic availability of sirolimus is low, and was estimated to be approximately 14% after the administration of rapamune oral solution. In healthy subjects, the mean bioavailability of sirolimus after administration of the tablet is approximately 27% higher relative to the solution. Sirolimus tablets are not bioequivalent to the solution [7].

2.1.9 Sirolimus dosing

According to FDA package inserts [7], sirolimus is to be administered orally once daily consistently with or without food and is extensively partitioned into formed blood elements. Sirolimus is often dosed by weight and requires monitoring during initial therapy until steady-state levels of drug are achieved on a maintenance dose. Loading doses are sometimes used to more rapidly reach desired trough concentrations when used as prophylaxis to reduce solid organ transplant rejection. Once the sirolimus maintenance dose is adjusted, patients should continue on the new maintenance dose for at least 7 to 14 days before further dosage adjustment with concentration monitoring. It is recommended that the maintenance dose of sirolimus be reduced by approximately one-third in patients with mild or moderate hepatic impairment and by approximately one-half in patients with severe hepatic impairment. It is not necessary to modify the sirolimus loading dose. Dosage adjustment is not needed in patients with impaired renal function. At doses of 2 mg/day and 5 mg/day, sirolimus significantly reduced the incidence of organ rejection in low- to moderate-immunologic risk renal transplant patients at 6 months following transplantation compared with either azathioprine or placebo [7].

2.1.10 Sirolimus metabolism and drug-drug interactions

Sirolimus is usually avoided with concomitant use of strong inducers (e.g., rifampin and rifabutin) and strong inhibitors (e.g., ketoconazole, voriconazole, itraconazole, erythromycin, and clarithromycin) of CYP3A4 and P-gp. Grapefruit juice is also contraindicated during sirolimus therapy. Drugs that could increase sirolimus blood concentrations include cimetidine, cisapride, clotrimazole, diltiazem, fluconazole, PIs (including those used to treat HIV and HCV), metoclopramide, nicardipine, troleandomycin and verapamil (verapamil levels may also increase when given with sirolimus). Drugs that could decrease sirolimus concentrations include carbamazepine, phenobarbital, phenytoin, rifapentine, NNRTIs and St. John's wort.

2.2 Rationale

Table 1. HIV-1 Persistence and the Potential Benefits of Sirolimus on HIV-1 Reservoirs

Obstacle to HIV Reservoir Reduction	Potential Effect of Sirolimus	Immune Evaluation	Potential Beneficial Impact on HIV-1 Reservoir Size	Related Objectives
Ongoing viral replication in activated reservoir	Decreased immune activation (cytokine mediated); reduction of CCR5 expression	Markers of immune activation and/or proliferation, soluble markers of inflammation	Decreased transcription/ translation and CA-RNA; decreased CA-DNA	1.2.3 (P)<br>1.3.1 (S)<br>1.3.4 (S)<br>1.4.4 (E)
Blunted immune mediated clearance	Increased CD8+ central and effector memory response; improved HIV-1-specific CD8+ cell immunity; generation of novel immune responses; CD4+ cell differentiation to regulatory CD25+Fox-P3+ cells; enhanced viral specific humoral immunity and inhibits class switching in B cells	HIV-1-specific CD8+ cell immunity; HIV-1-specific CD4+ T-cell immunity; transcriptional profiling; HIV-specific Ab quantitation and B cell phenotyping	Increased clearance of HIV-infected cells (decreased CA-DNA, RNA and residual viremia), plasma RNA	1.2.2 (P)<br>1.3.1 (S)<br>1.3.3 (S)<br>1.4.5 (E)
Negative regulators; immune exhaustion	Decreased PD-1 and dysfunctional T-cell signatures	PD1; PD-L1	Decreased CA-DNA and other markers of reservoir size	1.2.2 (P)<br>1.3.1 (S)<br>1.3.4 (S)
Homeostatic proliferation	Decreased proliferation of latent reservoir	T-cell immune subset activation and proliferation; homeostatic prolif.-regulating cytokines; STAT-mediated signaling	Decreased CA-DNA and other markers of reservoir size	1.3.4 (S)<br>1.4.2 (E)<br>1.4.3 (E)
Objective: P = primary, S = secondary, E = exploratory				

2.2.1 Rationale for the potential effect on HIV latency

Given that the cell-cycle effects of sirolimus appear to be limited to T cells that are activated by cytokines, and data indicating that generalized T-cell activation in HIV disease is cytokine rather than antigen-driven [23], the mechanism of action of sirolimus may be particularly beneficial in HIV infection. Also, in contrast to cyclosporine, major histocompatibility complex (MHC) class I or class II restricted antigen presentation by dendritic cells is not inhibited by sirolimus, at least in vitro **[44],** which may preserve the capacity of the immune system to generate novel T-cell responses, prevent CD8+ cell exhaustion, and allow the preservation of an intact adaptive immune response [**45].** Sirolimus also appears to enhance T regulatory cell function in humans, which may have both beneficial and harmful effects on immune function [**46, 47].**

Inhibition of mTOR will likely prevent differentiation of naïve T cells and central memory T (TCM) cells to the different CD4+ effector T-cell lineages as the latter

differentiation steps are metabolically demanding. Sirolimus inhibits STAT-mediated signaling and thus could inhibit homeostatic proliferation characterized by cytokines, such as IL-7 and IL-15, which is in part responsible for the maintenance of the pool of latently infected cells. These combined effects could collectively decrease HIV burden by preventing the generation of cells that might be more likely to support HIV replication, and by preventing homeostatic proliferation of latently-infected TCM cells. Indeed clonal expansion of resting T cells may be an important mechanism for HIV persistence. Two separate groups have recently shown identical HIV integration sites in a limited number of genes which accounted for a large proportion of the infected T cells in those patients **[48, 49].**

2.2.2 Rationale for other potential mechanisms through which sirolimus may reduce HIV-1 reservoirs

We have recently identified additional potential avenues through which sirolimus treatment could potentially reduce the HIV-1 reservoir in virally suppressed patients. We have shown that ex vivo sirolimus treatment of purified and stimulated CD4+ and CD8+ cells from chronically infected patients on ART results in the down-regulation of PD-1 expression and expansion of central memory subsets [Chomont N and Sekaly RP, unpublished data]. PD-1 is involved in the triggering of a dysfunctional T-cell signature and plays a role in the establishment and maintenance of the HIV-1 reservoir **[50,51]**. Sirolimus treatment has also been shown to down-regulate PD-L1 expression (ligand for PD-1) on both myeloid- and monocyte-derived dendritic cells, further expanding the possibility of potential rescue of CD8+ effector function [**52**]. Furthermore, our preliminary data have shown that CD8+ cells from HIV-1 elite controllers show a transcriptional profile that is characterized by the down-regulation of the mTOR signal transduction pathway, a phenotype that is generated by exposure of T cells to sirolimus. It is possible that decreased activation of mTOR signaling in the T cells of elite controllers is partially responsible for superior viral control and reduced reservoir size in these patients. **Therefore, we will explore expression of genes in the mTOR pathway in study participants prior to receiving and while on sirolimus.**

Taken together, these findings suggest that sirolimus has anti-HIV effects by improving HIV-1 cellular immunity while reducing overall cytokine-mediated inflammation and T-cell activation and proliferation; sirolimus therapy may have significant effects on the latent HIV-1 reservoir.

2.2.3 Rationale for the impact of sirolimus on HIV DNA levels during ART

In order to determine the potential role of sirolimus and other immune-suppressing agents in accelerating the decay of the HIV reservoir, we analyzed cellular and plasma HIV-1 RNA and DNA levels in a cohort of ARV-treated adults who underwent renal transplantation [**53].** All individuals in this cohort were on ART, had a CD4+ cell count ≥200 cells/mm^3 and had undetectable plasma HIV-1 RNA using conventional assays. The decision as to which regimen was used was not standardized across subjects. Most subjects received a combination

regimen that included cyclosporine, tacrolimus or sirolimus, mycophenolate, and/or prednisone.

From this cohort, we identified those subjects (n=91) who were on a stable ART regimen at study entry and who did not interrupt ART for more than 3 days in the post-transplant period. The median pre-transplant CD4+ cell count was 510 cells/mm^3. Immunosuppressive drugs used at week 12 included cyclosporine (21%), tacrolimus (74%), sirolimus (9%), mycophenolate mofetil (73%), and prednisone (76%). Of note, the calcineurin inhibitor (cyclosporine or tacrolimus) was discontinued in 9% of subjects due to toxicity, and replaced by sirolimus. At any study time point, a maximum of nine subject received sirolimus treatment.

Predictors of HIV reservoir size (which included CD4+ cell count and exposure to various immunosuppressive drugs as time-dependent covariates) over the 2-year period post-transplant were examined using univariate and multivariate linear repeated measures models. Subjects were censored at the time of virologic failure or graft loss. All variables with $p<0.1$ from the univariate model were included in an initial multivariate model. For normality of the response variable, $\log_{10}$ (for plasma HIV-1 RNA) and reciprocal (for cell-associated RNA and proviral DNA) transformations were used. In the multivariate model, higher baseline plasma HIV RNA level ($p<0.0001$) and white race ($p=0.01$) were associated with increased plasma HIV-1 RNA levels post-transplant.

Figure 1: HIV-1 DNA levels by sirolimus use post-transplant. Sample sizes represent HIV-1 DNA measurements at all time points.

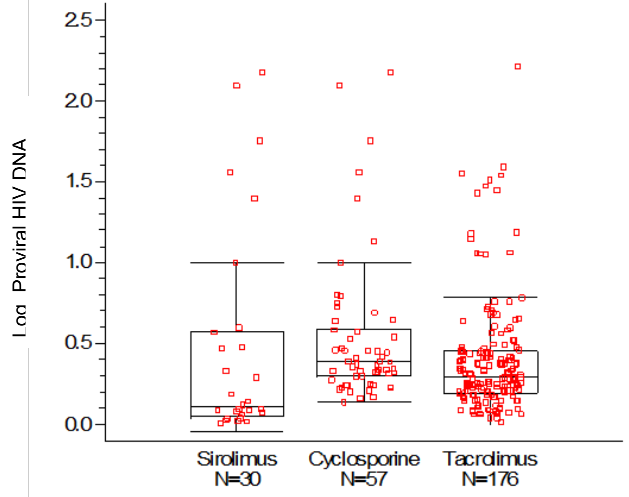

Figure 2: Proviral HIV DNA level over time by sirolimus exposure.

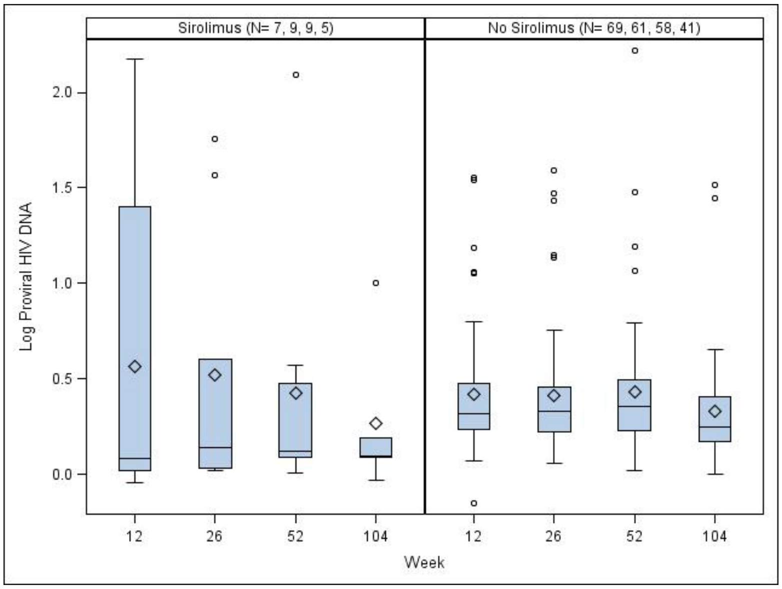

Sirolimus use (p=0.04) and longer duration of follow-up post-transplant (marginally; p=0.06) were associated with a decreased proviral DNA level in the multivariate model (model adjusted for baseline HIV DNA, baseline CD4+ cell count, time-varying sirolimus use, and study week); higher baseline proviral HIV DNA (p<0.0001) and lower CD4+ cell count (p=0.001) were associated with increased proviral HIV DNA level post-transplant. Sirolimus use was also significantly associated with lower proviral DNA in univariate analysis (p=0.02).

As compared to subjects on other immune-modulating drugs such as cyclosporine and tacrolimus, the relative difference in HIV DNA levels was approximately 0.3 log_{10} copies/mL (see Figure 1). Figure 2 shows the proviral HIV DNA level over time by sirolimus exposure. No significant predictors of cell-associated RNA post-transplant were identified in the multivariate model (data not shown). These post-hoc analyses have several limitations, including the lack of consistent use of sirolimus monotherapy among the study population. As a result, the findings may have been influenced by various uncontrollable biases, and further data regarding sirolimus use and viral reservoirs from the kidney transplant population are not available. Taken together, a proof-of-concept study of sirolimus in HIV-infected individuals on suppressive ART is warranted to elucidate the safety and efficacy of mTOR inhibition on latent reservoirs and anti-HIV immune responses and inflammation.

2.2.4 Rationale for the safety of sirolimus in HIV-infected individuals

There is growing evidence suggesting that long-term sirolimus monotherapy in HIV-infected individuals on ART is safe. For example, seven participants with KS underwent up to 12 cumulative months of sirolimus therapy while on suppressive ART [14]. During therapy, no significant increases in viral load measurements were observed and CD4+ cell declines were mild and transient for a majority of study participants (total counts from all participants remained above 300 cells/uL). CD4+ cell counts returned to pre-sirolimus levels on therapy, and treatment was well tolerated overall. Although all participants had at least one AE, most were mild (Grade 1) with Grade 3 AEs occurring in two participants. One AE was a dental abscess that likely was present before sirolimus initiation and one participant had lower extremity superficial venous thrombosis. Another participant required oral antibiotic treatment for pneumonia (Grade 2 AE), but recovered and continued on sirolimus throughout the study [14]. Another study of HIV individuals undergoing liver transplantation observed ten individuals who received sirolimus therapy to prevent graft rejection. Two participants stopped sirolimus therapy secondary to wound infection or thrombocytopenia [15]. However, these participants were on concurrent immunosuppressive therapies in addition to sirolimus (cyclosporine/tacrolimus) which made it difficult to ascribe specific events to a single drug. A more recent, large prospective study of renal transplantation in HIV-infected individuals on ART included 68 participants on sirolimus immunosuppressive therapy, including some who tolerated years of immunosuppression **[54].** Although there was a high rate of graft rejection noted in the study population, AEs to immunosuppression or changes in viral or immunologic parameters were not addressed in detail.

2.2.5 Rationale for the exclusion of patients on PI-based therapies and potential drug-drug interactions

Sirolimus, which is metabolized by the cytochrome P450 system, has significant drug-drug interactions, predominately with the PI class of ARV drugs. PIs significantly increase the area under the curve (AUC) and maximum concentration (C_{max}) of sirolimus and therefore will be prohibited in this study. NNRTIs can potentially lead to decreased levels of sirolimus, but dosage adjustment up for sirolimus and careful monitoring of sirolimus levels as

proposed will be sufficient to overcome potential interactions [**55,56].** In the recent sirolimus monotherapy study for KS, the three participants who were on suppressive NNRTI regimens without PIs had maximum drug trough levels of 7 to 13, all of which are in or near the accepted, safe therapeutic range used for transplant rejection [14]. Furthermore, these participants were on steady-state doses of sirolimus that mirror those of HIV-negative participants not on ART. The exact effects of sirolimus on NNRTI or NRTI levels are not well described, but all participants, even those on PI-based regimens with inconsistent drug levels, had stable, sustained viral control during sirolimus treatment [14].

2.2.6 Rationale for stability of virologic and immunologic measurements while on suppressive ART

HIV-specific CD8+ cells frequently decline after initiation of ART [**57]** but recent unpublished data from ACTG 5281 suggest that these T-cell frequencies stabilize on suppressive ART and demonstrate little change over 14 weeks of observation [J. Jacobson, personal communication]. Furthermore, no significant changes in the levels of cell-associated RNA in patients on suppressive ART have been observed when measured at intervals 2 weeks to 2 months apart [J. Mellors, personal communication]. Although the prevalence of detectable low level residual plasma viremia varies between studies incorporating the use of the single-copy RNA assay, stability of residual viremia on suppressive ART has been demonstrated in several studies [**58].** These findings support the assumption that there would be no change or zero mean change in these measurements in a placebo arm if one were to be incorporated.

2.2.7 Rationale for evaluation of anal dysplasia

The most important risk factor for HPV-related anogenital dysplasia in HIV-infected individuals is persistent HPV infection. If sirolimus reduces the amount or the presence of oncogenic HPV DNA, it is possible that regression of HPV-associated lesions will follow. Anal disease is being assessed because of ease of evaluation.

3.0 STUDY DESIGN

A5337 is a phase I/II open-label, single-arm, pilot study of sirolimus therapy for 20 weeks in 30 HIV-infected individuals suppressed on non-PI continuous ART for ≥24 months prior to study entry with CD4+ cell count **≥350** cells/mm^3. Continuous ART is defined as active therapy for the 24-month period prior to study entry with no treatment interruption longer than **7** consecutive days.

There is a 12-week pre-sirolimus lead-in period to define the steady-state level of HIV-1 pre-intervention, given planned paired analysis of primary outcomes before and after sirolimus treatment. Study entry is considered the time at which the 12 week pre-sirolimus period commences. Sirolimus therapy will be initiated at study week 12. In order to achieve therapeutic levels, therapy will be initiated with lead-in dose of 0.025 mg/kg/day for participants on a non-PI, non-NNRTI regimen (e.g., raltegravir) and for those on a non-PI, RPV-based regimen, and with 0.05 mg/kg/day for participants on an

NNRTI regimen with the exception of RPV. Doses will be adjusted based on trough blood sirolimus concentrations to achieve target concentrations between 5 and 10 ng/mL as described in section 5.1, Table **4.**

Sirolimus levels will be checked by FDA-approved clinical laboratory assays in a Clinical Laboratory Improvement Amendments (CLIA) certified laboratory. For safety and ease of dosing, **participants** will be recruited and followed only at medical centers that have rapid sirolimus testing available via liquid chromatography/tandem mass spectrometry (LC tandem MS), which enables accurate and consistent measures of levels across study sites. Sites without this capability are permitted to ship to Quest Diagnostic Laboratories for LC tandem MS testing. Results from local laboratories or from Quest should be available ideally within 72 hours. As there is more than one commercial assay available, interpretation will be based on the local site laboratory result. Results which are quickly available after sample receipt ensures accurate and consistent measures of levels across study sites. In addition, samples will be stored weekly for ARV drug level testing. Given the relatively small size of this pilot study and the stable plasma viral loads from previous sirolimus monotherapy studies in **participants** on ART, there are no plans to alter ART doses during the study. Drug level testing will be performed post study. Safety outcomes will be defined as the rate of new Grade ≥3 AEs, including signs/symptoms, lab toxicity or clinical event, that is definitely, probably or possibly related to study treatment, as judged by the core team, or by change (confirmed >50% decline or to <300 cells/mm^3) in CD4+ cell counts while on sirolimus.

4.0 SELECTION AND ENROLLMENT OF **PARTICIPANTS**

4.1 Inclusion Criteria

4.1.1 HIV-1 infection, documented by any licensed rapid HIV test or HIV enzyme or chemiluminescence immunoassay (E/CIA) test kit at any time prior to study entry and confirmed by a licensed Western blot or a second antibody test by a method other than the initial rapid HIV and/or E/CIA, or by HIV-1 antigen, plasma HIV-1 RNA viral load.

WHO (World Health Organization) and CDC (Centers for Disease Control and Prevention) guidelines mandate that confirmation of the initial test result must use a test that is different from the one used for the initial assessment. A reactive initial rapid test should be confirmed by either another type of rapid assay or an E/CIA that is based on a different antigen preparation and/or different test principle (e.g., indirect versus competitive), or a Western blot or a plasma HIV-1 RNA viral load.

4.1.2 Currently on continuous ART for ≥24 months prior to study entry. This is defined as continuous active therapy for the 24-month period prior to study entry with no treatment interruption longer **than 7 consecutive days.**

4.1.3 CD4+ cell count ≥**350** cells/mm^3 obtained within 60 days prior to study entry at any US laboratory that has a CLIA certification or its equivalent, or at any

network-approved non-US laboratory that operates in accordance with Good Clinical Laboratory Practices (GCLP) and participates in appropriate external quality assurance programs.

4.1.4 Plasma HIV-1 RNA below the level of quantification (e.g., <20, <40, <50, or <75 copies/mL depending on the assay) for ≥24 months by an FDA-approved assay at any US laboratory that has a CLIA certification or its equivalent, or at any network-approved non-US laboratory that operates in accordance with GCLP and participates in appropriate external quality assurance programs. Participants must have at least one documented HIV-1 RNA below the level of quantification obtained 12-24 months prior to screening **HIV-1 RNA** and one HIV-1 RNA less than the level of quantification obtained within 12 months prior to the screening HIV-1 RNA **by an FDA-approved assay** (see section 4.1.5)

<u>NOTE:</u> One month = 30 days.

Two plasma HIV-1 RNA measurements above the limit of quantification but <**500** copies/mL **("two blips")** in the 24 months prior to screening are allowed if directly **preceded and** followed by HIV-1 RNA below assay limit**. The screening value may serve as the subsequent below-assay-limit value following a blip.**

4.1.5 Plasma HIV-1 RNA level of <40 copies/mL obtained by the Abbott real time assay or <20 copies/mL by the Roche COBAS TaqMan HIV-1 Test, Version 2.0 assay within 60 days prior to study entry at any laboratory that has a CLIA certification or its equivalent.

4.1.6 For females of reproductive potential (defined as women who have not been postmenopausal for at least 24 consecutive months or documentation that the woman has undergone hysterectomy, bilateral oophorectomy, or salpingectomy), negative serum or urine pregnancy test within 48 hours prior to study entry.

<u>NOTE:</u> **Participant-**reported history is acceptable documentation of hysterectomy and bilateral oophorectomy, tubal ligation, tubal micro-inserts, vasectomy and menopause.

4.1.7 Females of reproductive potential who are participating in sexual activity that could lead to pregnancy must agree to initiate effective contraceptives before sirolimus therapy, continue use during sirolimus therapy and maintain use for at least 12 weeks after sirolimus therapy has been stopped.

Female **participants** and/or their male partners MUST agree to use appropriately at least one of the following:

- Condoms (male or female) with or without a spermicidal agent
- Diaphragm or cervical cap with spermicide
- Intrauterine device (IUD)

- Tubal ligation
- Hormone-based contraceptive

NOTE:

- Sexual activity with an infertile partner is not sexual activity that can lead to pregnancy.
- Females on hormone-based contraceptives at study entry must have been on the same method for at least 90 days prior to study entry.

4.1.8 Men and women ≥18 years of age.

4.1.9 Ability and willingness of **participant** or legal guardian/representative to provide informed consent.

4.1.10 Laboratory evaluations obtained within 60 days prior to entry by any US laboratory that has a CLIA certification or its equivalent, or at any network-approved non-US laboratory that operates in accordance with GCLP and participates in appropriate external quality assurance programs.

- White blood cell (WBC) ≥3000/mm^3
- Platelet count ≥125,000/mm^3
- ANC >1300/mm^3
- Aspartate aminotransferase (AST) <1.25 x ULN
- Alanine aminotransferase (ALT) <1.25 x ULN
- Calculated creatinine clearance (CrCl) ≥60 mL/min as estimated by the Cockcroft-Gault equation:
  For men, (140 – age in years) x (body weight in kg) ÷ (serum creatinine in mg/dL x 72) = CrCl (mL/min)*
  *For women, multiply the result by 0.85 = CrCl (mL/min)
  NOTE: A program to assist in calculations is available on the DMC website at: http://www.fstrf.org/ACTG/ccc.html
- Fasting or non-fasting triglyceride level ≤350 mg/dL
- Fasting or non-fasting LDL <160 mg/dL
- Urine protein to urine creatinine ratio **≤**1 **g/g** from random urine collection

4.2 Exclusion Criteria

4.2.1 Serious illness requiring systemic treatment and/or hospitalization until **participant** either completes therapy or is clinically stable on therapy in the opinion of the site investigator for at least 30 days prior to study entry.

4.2.2 Documentation of any CDC Category C AIDS-indicator condition or oropharyngeal candidiasis (thrush) within 90 days prior to study entry. (http://www.cdc.gov/mmwr/preview/mmwrhtml/00018871.htm)

4.2.3 Intended modification of ART during the study.

4.2.4 Latent tuberculosis (TB) infection defined as a positive purified protein derivative (PPD) ≥5 mm or positive interferon-gamma release assay (IGRA) at any time in the past or evidence of latent TB on the screening chest x-ray without subsequent isoniazid or equivalent antibiotic prophylaxis.

NOTE: Prophylaxis must have been completed at least 48 weeks prior to study entry.

4.2.5 TB disease within 48 weeks prior to study entry requiring treatment. **Participants** with a history of active TB must have completed treatment at least 48 weeks prior to study entry.

4.2.6 History of or current (within 90 days prior to study entry) active hepatitis B (HBV) infection defined as positive HBV surface antigen test or positive HBV DNA in **participants** with isolated HBcAb positivity.

4.2.7 HCV RNA-positive within 90 days prior to study entry.

NOTE: **Participants** who are HCV antibody negative within 90 days prior to study entry are eligible for the study. Those who are not taking HCV therapy and who are HCV antibody-positive but HCV RNA negative within 90 days prior to study entry are eligible for the study.

4.2.8 Previously diagnosed myelodysplasia syndrome.

4.2.9 History of lymphoproliferative disease prior to study entry.

4.2.10 Clinically significant lung disease on the screening chest x-ray that, in the opinion of the site investigator, places the **participant** at increased risk of lung toxicity (e.g.,history of pulmonary fibrosis, interstitial lung disease or pulmonary lymphoproliferative disease).

4.2.11 Any prior or current diagnosis of solid tumor or hematologic malignancies, **excluding a history of fully excised or resolved non-melanoma skin cancers without evidence of prior or current metastasis.**

4.2.12 History of congestive heart failure as defined by physician documentation in the medical record at any time prior to screening that required medication for heart failure or that required medical management within 2 years prior to study entry.

4.2.13 Detectable Epstein-Barr virus (EBV) in blood by polymerase chain reaction (PCR) within 90 days prior to study entry at any US laboratory that has a CLIA certification or its equivalent.

4.2.14 Active infection other than HIV that required receipt of systemic antibiotic therapy by intravenous infusion within 90 days prior to study entry.

4.2.15 Life-threatening fungal infection that in the opinion of the site investigator requires treatment within 48 weeks prior to study entry.

4.2.16 Herpes-zoster or varicella-zoster viral infection requiring treatment within 90 days prior to study entry or currently on suppressive therapy.

4.2.17 History of major hypersensitivity reaction to macrolide drugs including angioedema, anaphylaxis, drug-induced dermatitis or hypersensitivity vasculitis.

4.2.18 Currently pregnant or breastfeeding, or planning to become pregnant prior to or during the study.

4.2.19 Use of immunomodulators (e.g., interleukins, interferons and cyclosporine), HIV vaccine, systemic cytotoxic chemotherapy, or investigational therapy within 90 days prior to study entry.

4.2.20 Active drug or alcohol use or dependence that in the opinion of the site investigator would interfere with adherence to study requirements.

4.2.21 Vaccination (e.g., pneumococcal polysaccharide/influenza vaccine) within 14 days prior to study entry.

NOTE: If **participants** receive influenza vaccination for routine clinical care during or prior to the screening visit, they may be rescreened 14 days after vaccination.

4.2.22 On a PI-based ART or cobicistat-boosted regimen within 90 days prior to study entry or plans to change to a PI-based or cobicistat-boosted regimen during the study.

NOTE: Prior PI-based or cobicistat-boosted regimens are allowed.

4.2.23 Anal or perianal administration of anti-HPV therapies (e.g., imiquimod, 5FU, veregen) for 90 days prior to study entry or plans to initiate anti-HPV therapies during the study.

4.3 Study Enrollment Procedures

4.3.1 Prior to implementation of this protocol and any subsequent full version amendments, each site must have the protocol and the protocol consent form approved, as appropriate, by their local institutional review board (IRB)/ethics committee (EC) and any other applicable regulatory entity (RE). Upon receiving final approval, sites will submit all required protocol registration documents to the DAIDS Protocol Registration Office (PRO) at the Regulatory Support Center (RSC). The DAIDS PRO will review the submitted protocol registration packet to ensure that all of the required documents have been received. Protocol activation may be required before each site can enroll any participants.

Site-specific informed consent forms (ICFs) will be reviewed and approved by the

DAIDS PRO and sites will receive an Initial Registration Notification from the DAIDS PRO that indicates successful completion of the protocol registration process. A copy of the Initial Registration Notification should be retained in the site's regulatory files.

Upon receiving final IRB/EC and any other applicable RE approvals for an amendment, sites should implement the amendment immediately. Sites are required to submit an amendment registration packet to the DAIDS PRO at the RSC. The DAIDS PRO will review the submitted protocol registration packet to ensure that all the required documents have been received. Site-specific ICFs will not be reviewed and approved by the DAIDS PRO and sites will receive an Amendment Registration Notification when the DAIDS PRO receives a complete registration packet. A copy of the Amendment Registration Notification should be retained in the site's regulatory files.

For additional information on the protocol registration process and specific documents required for initial and amendment registrations, refer to the current version of the DAIDS Protocol Registration Manual.

Once a candidate for study entry has been identified, details will be carefully discussed with the participant. The participant (or, when necessary, the legal guardian if the participant is under guardianship) will be asked to read and sign the approved protocol consent form.

For participants from whom a signed informed consent has been obtained, an ACTG Screening Checklist must be entered through the Data Management Center (DMC) Participant Enrollment System.

For candidates from whom informed consent has been obtained, but who are deemed ineligible or who do not enroll into the protocol, an ACTG Screening Failure Results form must be completed and keyed into the database.

4.3.2 Protocol Activation

Prior to enrollment, sites must complete the Protocol Activation Checklist found on the ACTG Member website. This checklist must be approved prior to any screening of participants for enrollment.

4.3.3 **Participant** Registration

Participants who meet eligibility criteria for A5337 will be registered to A5337 according to standard ACTG DMC procedures.

4.4 Coenrollment Guidelines

- A5128, “Plan for Obtaining Informed Consent to Use Stored Human Biological Materials (HBM) for Currently Unspecified Analyses”: Coenrollment in A5128 is encouraged and does not require permission from the A5337 protocol chairs.

- A5321, Decay of HIV-1 Reservoirs in Subjects on Long-Term Antiretroviral Therapy: The ACTG HIV Reservoirs Cohort Study, and/or A5322, Long-Term Follow-up of Older HIV-infected Adults in the ACTG: Addressing Issues of Aging, HIV Infection and Inflammation, do not require permission from the A5337 protocol chairs provided that sites can remain within site-specific blood volume limits
- **A5332 (REPRIEVE), Randomized Trial to Prevent Vascular Events in HIV, is allowed as long as participants are not enrolled in any of the REPRIEVE immunology substudies.**
- **A5351s, Effect of Immune-Modulatory Interventions on Cytomegalovirus Replication during Suppressive Antiretroviral Therapy. Coenrollment in A5351s is encouraged and does not require permission from the A5337 protocol chairs.**
- For specific questions and approval for coenrollment in other studies, sites should first check the A5337 PSWP or contact the protocol team via e-mail as described in the Study Management section.

5.0 STUDY TREATMENT

5.1 Regimens, Administration and Duration

Eligible **participants** will be stratified at study entry by class of ART regimen as follows:

Participants taking a non-PI, non-NNRTI containing regimen, and those on a non-PI, RPV-based regimen, will initially receive:

- Sirolimus 0.025 mg/kg/day administered orally once daily with or without food. See Table 2.

Participants taking an NNRTI containing regimen (with the exception of RPV) will initially receive:

- Sirolimus 0.05 mg/kg/day administered orally once daily with or without food. See Table 3.

In order to achieve therapeutic levels, therapy will be initiated with the dosage noted above and dosing will be subsequently adjusted based on trough blood sirolimus concentrations to achieve target concentrations between 5 and 10 ng/mL (see **Table 4).** A detailed dosing table based on the adjustment algorithm in **Table 4** can be found on the A5337 PSWP.

Table 2. Sirolimus Dosing at 0.025mg/kg			
**Weight**	**Initial daily dose**	**25% increase***	**25% reduction***
**50 to 70 kg**	**1.5 mg**	**2 mg**	**1 mg**
**71 to 90 kg**	**2.0 mg**	**2.5 mg**	**1.5 mg**
**91 to 110 kg****	**2.5 mg**	**3 mg**	**2.0 mg**

***Dose adjustment based on sirolimus trough level.**
****For participants weighing >110 kg, the sirolimus dose needs to be clarified by the team.**

Table 3. Sirolimus Dosing at 0.05mg/kg			
**Weight**	**Initial daily dose**	**25% increase***	**25% reduction***
**50 to 70 kg**	**3 mg**	**4 mg**	**2 mg**
**71 to 90 kg**	**4 mg**	**5 mg**	**3 mg**
**91 to 110 kg****	**5 mg**	**6.5 mg**	**4 mg**

***Dose adjustment based on sirolimus trough level**
****For participants weighing >110 kg, the sirolimus dose needs to be clarified by the team.**

Table 4. Sirolimus Dose Adjustment Algorithm	
**Sirolimus level**	**Dose adjustment***
**<5 ng/mL**	**Increase dose by 25%,**
**5-10 ng/mL**	**Continue current dose**
**>10, <15 ng/mL**	**Reduce dose by 25%**
**>15, <20/ng/mL**	**Hold dose for 48 hours, then decrease dose by 25%**
**>20/ng/mL**	**Hold dose until next level check, if <12 ng/mL, then restart study drug at 50% prior dose**

***Round dose up to closest sirolimus 0.5mg tablet. Sirolimus tablets cannot be split, crushed or chewed.**

Participants will initiate study treatment at week 12 and continue for 20 weeks. Participants will be followed off-treatment for an additional 12 weeks for a total of 44 weeks. Treatment must be initiated within 72 hours of week 12 (day 0) evaluations (see section 6.2.3).

5.2 Study Product Formulation and Preparation

Sirolimus tablets are obtained from Greenstone LLC. The inactive ingredients include sucrose, lactose, polyethylene glycol 8000, calcium sulfate, microcrystalline cellulose, pharmaceutical glaze, talc, titanium dioxide,

magnesium stearate, povidone, poloxamer 188, polyethylene glycol 20,000, glyceryl monooleate, carnauba wax, di-alpha tocopherol and other ingredients.

Sirolimus tablets 0.5 mg will be provided. These tablets also contain yellow iron (ferric) oxide and brown iron (ferric) oxide. They should be stored at 20° to 25°C [USP Controlled Room Temperature] (68° to 77°F). Dispense in a tight, light-resistant container as defined in the USP.

5.3 Pharmacy: Product Supply, Distribution, and Accountability

5.3.1 Study Product Acquisition/Distribution

Sirolimus will be available through the NIAID Clinical Research Products Management Center (CRPMC). The site pharmacist should obtain the study product(s) for this protocol by following the instructions in the manual *Pharmacy Guidelines and Instructions for DAIDS Clinical Trials Networks.*

Any study product not provided by the study must comply with the NIAID (DAIDS) policy that outlines the process for authorizing the use of study products not marketed in the US in NIAID (DAIDS)-supported and/or –sponsored clinical trials.

5.3.2 Study Product Accountability

The site pharmacist is required to maintain complete records of all study products received from the NIAID CRPMC and subsequently dispensed. All unused study products in US CRSs must be returned to the NIAID CRPMC (or as otherwise directed by the sponsor) after the study is completed or terminated. The procedures to be followed are in the manual *Pharmacy Guidelines and Instructions for DAIDS Clinical Trials Networks.*

5.4 Concomitant Medications

Whenever a concomitant medication or study agent is initiated or a dose changed, investigators must review the concomitant medication's and study agent's most recent package insert, Investigator's Brochure, or updated information from DAIDS to obtain the most current information on drug interactions, contraindications, and precautions.

Additional drug information may be found on the updated ACTG **Precautionary and Prohibited Medications** Database located at:
http://tprc.pharm.buffalo.edu/home/di_search/

5.4.1 Required Medications

Continuous ART, defined as active therapy with no treatment interruption longer than **7** consecutive days and a total duration off ART of no more than 7 days, is required through week 44. Changes in the regimen are allowed as long as a PI-based or cobicistat-boosted regimen is not initiated.

5.4.2 Prohibited Medications

These medications should be discontinued prior to study entry week 0 and are prohibited through week 32:

- Verapamil or diltiazem
- Protease inhibitors
- Medications known to be potent CYP3A inhibitors, (e.g., ritonavir, cobicistat, ketoconazole, or erythromycin)
- Anal or perianal administration of anti-HPV therapies (e.g., imiquimod, 5FU, veregen)
- Use of immunomodulators such as interleukins, interferons, and cyclosporin, HIV vaccine, systemic cytotoxic chemotherapy or investigational therapy
- Tacrolimus, mycophenalate mofetil, TNF-modulating medications, plaquenil or monoclonal antibodies targeting lymphocytes
- HPV vaccine
- Standard-of-care vaccines (e.g., pneumococcal polysaccharide or influenza vaccine within 14 days prior to study entry or 14 days prior to any post-entry study visit)

5.4.3 Precautionary Medications

A list of precautionary medications can be found on the A5337 PSWP.

6.0 CLINICAL AND LABORATORY EVALUATIONS

6.1 Schedule of Events

Evaluation	Screening	Entry	Sirolimus Initiation	Post-Entry Evaluations							Premature Treatment or Study Discontinuation	Confirmation of CD4 Decrease	Confirmation of Virologic Failure
	Week	0	12	13	14	16	20	24	32	44			
Days Post-Sirolimus Initiation			0	4	11	28	56	84	140	224			
Window	Within 60 days prior to entry		±1 week	±1 day (same days as PK)			±1 week (same days as PK)			±2 weeks			
Documentation of HIV	X												
Medical & Medication History	X	X											
Clinical Assessment	X	X	X	X	X	X	X	X	X	X	X		
Complete Physical Exam	X												
Targeted Physical Exam		X	X		X	X	X	X	X		X	X	X
Hematology	X	X	X	X	X	X	X	X	X	X	X		
Liver Function Tests	X	X	X	X	X	X	X	X	X	X	X		
Blood Chemistry	X	X	X	X	X	X	X	X	X	X	X		
Calculated Creatinine Clearance	X	X	X	X	X	X	X	X	X	X	X		
Urinalysis	X		X			X	X	X					
Pregnancy Testing	X	X	X	if indicated									
Hepatitis Serology (see section 6.3.5)	X												
Assessment for Reactivation of Latent EBV	X						X	X	X	X	X		
CD4+/CD8+	X	X	X		X	X	X	X	X	X	X	X	

Evaluation	Screening	Entry	Sirolimus Initiation	Post-Entry Evaluations							Premature Treatment or Study Discontinuation	Confirmation of CD4 Decrease	Confirmation of Virologic Failure
	Week	0	12	13	14	16	20	24	32	44			
Days Post-Sirolimus Initiation			0	4	11	28	56	84	140	224			
Window	Within 60 days prior to entry		±1 week	±1 day (same days as PK)			±1 week (same days as PK)			±2 weeks			
Cryopreserved PBMCs for HIV-1 Gag-specific CD8+ and CD4+ cell Immunity		X	X	X		X		X	X	X	X		
Stored Plasma for Inflammatory and Coagulation Markers		X	X	X		X		X	X	X	X		
Cryopreserved PBMCs for Markers of T-cell Activation and Proliferation		X	X	X		X		X	X	X	X		
Cryopreserved PBMCs for Exploratory Studies		X	X	X		X	X	X	X	X	X		
Stored Plasma for Antibody Quantitation		X	X	X				X	X	X	X		
Plasma HIV-1 RNA	X	X	X			X	X	X	X	X	X		X
HIV-1 Drug Resistance Genotyping (see section 6.3.7)													X
Cryopreserved PBMCs for **Inducible RNA** Assay			X					X	X	X	X		
Cryopreserved PBMCs for Cell-Associated RNA and DNA		X	X	X		X		X	X	X	X		
Stored Plasma for Residual Viremia by Single-Copy RNA Assay		X	X	X		X		X	X	X	X		
**Stored Whole Blood for Gene Expression Assay**			**X**	**X**		**X**		**X**	**X**	**X**			
Stored Plasma and **PBMCs** for Future Unspecified Testing		X	X	X		X		X	X	X	X		
Tuberculosis Test (see section 6.3.11)	X												
Chest Radiograph	X												
Anal Swabs/Anal Pap Test			X						X				
**Oral Swabs**		**X**	**X**	**X**		**X**		**X**	**X**	**X**	**X**		

6.1.1 Pharmacokinetic Evaluations

See section 6.3.12 for additional details.

Pharmacokinetic (PK) Sampling															
Evaluation	Weeks 12-16										Weeks 18-32				
	Week	12	13	13	14	14	15	15	16	16	18	20	24	28	32
	Day	0	4	7	11	14	18	21	25	28					
Window		±1 day (timed with other study visits as in section 6.1									±1 week (timed with other study visits as in section 6.1)				
PK Whole Blood Samples for Trough Sirolimus Real-time Monitoring (see section 6.3.12			X	X	X	X	X	X	X	X	X	X	X	X	X
Stored Plasma for ARV PK (see section 6.3.12)		X	X		X					X	X		X	X	X

6.2 Timing of Evaluations

6.2.1 Screening Evaluations

Screening evaluations must occur before any study medications, treatments or interventions.

Screening evaluations to determine eligibility must be completed within 60 days prior to study entry, unless otherwise specified.

In addition to data being collected on **participants** who enroll into the study, demographic, clinical, and laboratory data on screening failures will be captured in a Screening Failure Results form and entered into the ACTG database.

6.2.2 Entry Evaluations

Entry evaluations, including collection of all entry specimens, must occur at least 48 hours after screening evaluations, unless otherwise specified, and prior to the **participant's** starting any study medications, treatments, or interventions.

6.2.3 Post-Entry Evaluations

Prior to starting sirolimus, laboratory measurements from the week 12 visit must be assessed to ensure that the criteria detailed in section 4.1.6 and 4.1.10 continue to be satisfied**,** as well as week 12 CD4+ cell count >300 cells/mm^3. If any criterion is not satisfied, the study team should be promptly contacted and sirolimus should not be started. In addition, concomitant medications must be re-assessed at the week 12 visit to ensure that the **participant** has not received any prohibited medications since study entry. If so, the study team should be promptly contacted and sirolimus should not be started.

Participants must begin study treatment within 72 hours after receipt of results from the study week 12 evaluations.

Site staff is encouraged to contact **participants** at least 14 days prior to scheduled study visits (not PK-only visits) to remind **participants** not to receive any vaccinations prior to their visit. A reminder can also be given that prohibited medications are not allowed at any time while on study.

For stored samples, all sites must have established procedures for regular reconciliation and verification of specimens, which must be followed throughout the study per the Lab Processing Chart (LPC). Collection of sample aliquots is of particular importance at the pre-sirolimus time points (entry and week 12) and at the study week 32 time point. In the event that the required volume or number of sample aliquots is not obtained at any time point, designated site clinic and/or lab staff must immediately inform the study team, who will provide guidance on how to respond to the problem.

In addition to following this guidance from the study team, designated site clinic and lab staff will work together to document the problem, take appropriate corrective and preventive action, and document all action taken. Reconciliation must be performed for all specimen types that are received by the laboratory and stored in the LDMS.

For other evaluations, if an evaluation is not completed at a scheduled visit, effort should be made to obtain the evaluation as soon as possible. If unsuccessful, complete the evaluation at the next study visit if not already included as part of the regularly scheduled visit.

NOTE: Sites must contact the A5337 core team prior to the scheduled week 12 visit, ideally at least 2 weeks before, for confirmation that a **participant** may begin study treatment based on availability of stored samples at study entry. This confirmation must be documented in the source document prior to initiation of sirolimus.

On-Treatment Evaluations (weeks 12 through 32)
The window for week 12 is ±1 week and for weeks 13 through 16 is ±1 day as visits will be timed with the PK evaluations. The window for weeks 20 through 32 is ±1 week.

NOTE: Starting with week 12, days should be counted from the first date of actual sirolimus dosing. Day 0 is the date of the first dose of sirolimus.

Post-Treatment Evaluations (week 44)
The week 44 visit is the final study visit. The window for the week 44 visit is ±2 weeks.

Event Driven Evaluations
Confirmation visit to confirm CD4+ cell count <300 cells/mm^3 or >50% CD4+ decrease from study entry value or HIV-1 RNA >200 copies/mL (see sections 7.1.4 and 7.1.5) should be scheduled within 1 week of receipt of the abnormal lab value. If confirmed CD4+ cell count <300 cells/mm^3 or >50% CD4+ decrease from study entry value or HIV-1 RNA >200 copies/mL, the study team should be promptly contacted and sirolimus should not be started or should be stopped if already started.

6.2.4 Discontinuation Evaluations

Evaluations for Registered **Participants** Who Do Not Start Study Treatment
Site personnel should notify the protocol team (actg.corea5337@fstrf.org) within 48 hours of any **participant** who is registered and does not begin treatment at week 12. All case report forms must be completed and keyed for the period up to and including week 12. **Participants** who do not start study treatment at week 12 will be taken off the study and will be replaced after completing all week 12 evaluations listed on the SOE.

Premature Treatment Discontinuation Evaluations

Participants who prematurely discontinue the study treatment prior to week 32 will have the Premature Treatment Discontinuation visit per the SOE, performed as soon as possible, and then continue study follow-up through week 44. **Participants** should have all PK sampling performed until the next scheduled dosing visit; then PK sampling should be discontinued. **Participants** will be followed on study/off study treatment.

Site personnel should notify the protocol core team (actg.corea5337@fstrf.org) within 48 hours of any **participant** who prematurely discontinues the study treatment.

Premature Study Discontinuation Evaluations

Participants who prematurely discontinue the study will have the Premature Study Discontinuation visit per the SOE, performed as soon as possible prior to being taken off the study.

Site personnel should notify the protocol core team (actg.corea5337@fstrf.org) within 48 hours of any **participant** who prematurely discontinues the study.

6.3 Instructions for Evaluations

All clinical and laboratory information required by this protocol is to be present in the source documents. Sites must refer to the Source Document Guidelines on the DAIDS Web site for information about what must be included in the source document: http://www.niaid.nih.gov/labsandresources/resources/daidsclinrsrch/documents/sourcedocappndx.pdf

All stated evaluations are to be recorded on the CRF and keyed into the database unless otherwise specified. This includes events that meet the International Council on Harmonisation (ICH) definitions for a serious adverse event:

- Results in death
- Life-threatening
- Requires inpatient hospitalization or prolongation of existing hospitalization
- Results in persistent or significant disability/incapacity
- Congenital anomaly/birth defect
- Other important medical event (may not be immediately life-threatening or result in death or hospitalization but may jeopardize the patient or may require intervention to prevent one of the events listed above.

To grade diagnoses, signs and symptoms, and laboratory results, sites must refer to the DAIDS Table for Grading the Severity of Adult and Pediatric Adverse Events (DAIDS AE Grading Table), **Version 2.0, November 2014** which can be found on the DAIDS RSC Web site: http://rsc.tech-res.com/safetyandpharmacovigilance/.

6.3.1 Documentation of HIV-1

Section 4.1.1 specifies assay requirements for HIV-1 documentation. HIV-1 documentation is not recorded on the CRF.

6.3.2 Medical History

The medical history must include all diagnoses within the 30 days prior to study entry identified by the ACTG criteria for clinical events and other diagnoses, as well as the following diagnoses, regardless of when the diagnosis was made, and recorded on a CRF. For current criteria, refer to the appendix identified on the study CRF.

- AIDS-defining conditions
- Bone fractures (verbal history accepted)
- Coronary heart disease
- Cancer
- Myelodysplastic, myeloproliferative or lymphoproliferative disorders
- Diabetes
- Tuberculosis
- Non-tuberculus mycobacterial infection
- Chronic hepatitis B
- Chronic hepatitis C
- Human papilloma virus (HPV; including cervical dysplasia/neoplasia and anogenital warts)
- Kidney disease
- Obstructive or restrictive lung disease
- Interstitial lung disease
- Endocrine disorders including fertility disorders
- Thyroid disease
- Treatment for HPV-related disease

Any allergies to any medications and their formulations must also be documented. **Document the pre-ART HIV-1 RNA level and CD4+ count, if available. If pre-ART HIV-1 RNA level or CD4+ count documentation is not available, then collect and record what the participant remembers. Document the date of first undetectable HIV-1 RNA level prior to sustained viral load suppression (may be estimated if exact date not available) and the date(s) of any previous virologic failure on ART. The medical history evaluation will be assessed at the screening and entry visit and recorded on the CRFs at the study entry visit.**

6.3.3 Medication History

A medication history must be present, including start and stop dates. The table below lists the medications that must be included in the history.

Medication Category	Timeframe
Antiretroviral therapy	Complete history<br>(start and stop dates required only for ART taken within past 2 years **and date of first use of ART)**
Immune-based therapy<br>(e.g., interleukins, interferons, cyclosporine, TNF-modulating drugs/monoclonal antibodies)	Complete history
HIV-1-related vaccines	Complete history
Drugs for treatment or prevention of opportunistic infections	Complete history
Prescription and non-prescription drugs	All cardiac or renal medications taken currently and within 12 months prior to study entry (including HMG-CoA reductase inhibitors, calcium channel blockers)<br><br>All other medications within 3 months prior to study entry
Dietary supplements	Within 3 months prior to study entry
Any prohibited medications. See section 5.4.2.	30 days prior to study entry

6.3.4 Clinical Assessments

Complete Physical Exam

A complete physical examination to include at a minimum an examination of the skin, head, mouth, and neck; auscultation of the chest; cardiac exam; abdominal exam; examination of the lower extremities for edema. The complete physical exam will also include signs and symptoms, diagnoses and vital signs (temperature, pulse, respiration rate, pulse oximetry and blood pressure).

Targeted Physical Exam

A targeted physical examination to include vital signs (temperature, pulse, respiration rate, pulse oximetry and blood pressure); examination of the skin, head, mouth, and neck; auscultation of the chest; cardiac exam; and is to be driven by any previously identified or new signs or symptoms and diagnoses that the **participant** has experienced since the last visit. Assessment of new pulmonary symptoms, including cough, shortness of breath and dyspnea on exertion will be included in the targeted exam.

Height
Height will be recorded at entry.

Weight
Weight will be recorded at entry and **all subsequent study visits.**

Signs and Symptoms
At entry, all grades of signs and symptoms that occurred within 30 days before entry must be recorded; post-entry, all grades of Grade ≥2 must be recorded. Record **on the CRF and key within 48 hours** all signs and symptoms regardless of grade that led to a change in study treatment or a change in ART. All Grade 3 or higher sign/symptom, any sign/symptom regardless of grade that led to a change in study treatment, or that met ICH, EAE or SAE guidelines, are defined by the protocol as reportable events that will require more detailed event reporting and keying within 48 hours.

Diagnoses
After entry, record all diagnoses identified by the ACTG criteria for clinical events and other diseases. Diagnoses must be recorded and keyed within 48 hours of the evaluation.

Concomitant Medications
After entry, record new or discontinued concomitant prescription and nonprescription drugs since the last visit.

ART Medication Modifications
At entry, record all ongoing ART. After entry, record all modifications in ART, including initial doses and/or missed doses (defined as more than 2 consecutive doses), inadvertent and deliberate interruption, and discontinuation.

Study Treatment Modifications
Record **on the CRF and key within 48 hours** all modifications in sirolimus dosing, including initial doses and/or missed doses, inadvertent and deliberate interruption and **permanent** discontinuation.

6.3.5 Laboratory Evaluations

Refer to the A5337 Laboratory Processing Chart (LPC) posted on the PSWP for processing, shipping, and storage information.

The screening and entry protocol-required laboratory values, regardless of grade, must be recorded. For post-entry assessments, record **on the CRF and key within 48 hours,** lipid values, glucose, hematology, liver function tests, calculated CrCl, serum creatinine values regardless of grade and all other Grade ≥3 laboratory values. In addition, record **on the CRF and key within 48 hours** all laboratory values regardless of grade that led to a change in study treatment or a change in ART. All Grade 3 or higher laboratory values, any laboratory value regardless of

grade that led to a change in study treatment, or that met ICH, EAE or SAE guidelines, are defined by the protocol as reportable events that will require more detailed event reporting and keying within 48 hours.

Hematology
Hemoglobin, hematocrit, white blood cell count (WBC) with differential, absolute neutrophil count (ANC), platelets.

Liver Function Tests
Total bilirubin, direct bilirubin, indirect bilirubin, albumin, AST (SGOT), ALT (SGPT), alkaline phosphatase, creatine kinase (CK).

Blood Chemistries
Glucose, triglycerides, cholesterol (total, LDL), electrolytes (e.g., sodium, potassium, chloride, bicarbonate), lactate dehydrogenase (LDH), creatinine, blood urea nitrogen (BUN), total protein, albumin, globulin (globulin may need to be calculated from the total protein and albumin). Lipid levels may be drawn non-fasting (see section 7.2.5 for management of elevated levels).

Calculated CrCl
Estimated each time that a serum creatinine level is determined. To estimate calculated CrCl, use the Cockcroft and Gault equation (see section 4.1.10).

Urinalysis
Random urinalysis for urine protein to urine creatinine ratio.

Pregnancy Test
For women with reproductive potential: At screening (before chest x-ray) negative serum or urine pregnancy test with a sensitivity range of ≤25 mIU/mL, 48 hours prior to study entry and again at week 12 with results available before initiation of study drug, and at any time during the study when pregnancy is suspected.

Hepatitis Serology
For **participants** with known HBV immunity, prior documentation of positive HBsAb is acceptable. If documentation is not available, HBsAb, HBsAg, and HBcAb will be obtained at screening. Negative HBV tests (i.e., negative HBsAb, HBsAg, and HBcAb) performed within 6 months prior to enrollment need not be repeated. If the last negative test result is more than 6 months prior to enrollment, then testing should be performed at screening. Results must be available prior to study entry so that **participants** who have active HBV can be excluded from participating in the study.

Participants who have positive HBcAb but negative HBsAg and HBsAb (isolated HBcAb positive status) must have HBV DNA PCR performed and confirmed as negative for **participant** to be eligible for A5337. HBV DNA PCR will not be provided by A5337.

HCV antibody test (and HCV RNA if the HCV Ab test is positive) should be obtained at screening. Results must be available prior to study entry.

Assessment for Reactivation Latent EBV
Performed on serum or plasma using a clinical quantitative PCR-based test at a laboratory that has CLIA certification or its equivalent (see section 7.1.6) and results from all testing recorded on a CRF.

6.3.6 Immunologic Studies

CD4+/CD8+
Obtain absolute CD4+/CD8+ count and percentages within 60 days prior to entry and per the SOE at any US laboratory that possesses a CLIA certification or its equivalent, or at any network-approved non-US laboratory that operates in accordance with GCLP and participates in appropriate external quality assurance programs.

During the study, all laboratories must possess a CLIA certification or equivalent and must be certified for protocol testing by the DAIDS Immunology Quality Assurance (IQA) Program.

Cryopreserved PBMCs for HIV-1 Gag-specific CD8+ and CD4+ cell Immunity
PBMC for flow cytometric testing of the frequency of HIV-1 Gag-specific and other non-specific responses in CD8+ and CD4+ cells by intracellular staining for IFN-gamma will be stored for analysis. In addition, HLA genotyping will be performed (entry visit only).

Stored Plasma for Inflammatory and Coagulation Markers
Plasma for soluble markers of inflammation (including IL-6, IL-7, IL-15) will be stored for analyses.

Cryopreserved PBMCs for Markers of T-cell Activation and Proliferation
Testing for T-cell activation and proliferation (% CD4+ and CD8+ cells CD38+/HLA-DR+, CD25+, PD-1+, PD-L1, Ki67+) will be performed concomitantly to the primary immunologic objectives.

Cryopreserved PBMCs for Exploratory Studies
B cell phenotyping, rearrangements and class switching determination will also be performed on cryopreserved cells as an exploratory objective.

Stored Plasma for Antibody Quantitation
Plasma will be stored for HIV-1-specific antibody quantitation including the use of assays that measure antibody levels, avidity and the breadth of antibody responses in peptide microarrays.

6.3.7 Virologic Studies

Plasma HIV-1 RNA
Screening HIV-1 RNA must be performed within 60 days prior to study entry by the Abbott RealTime HIV-1 assay or Roche COBAS TaqMan HIV-1 Test, Version 2.0 assay at any laboratory that possesses a CLIA certification or its equivalent.

After screening, all plasma HIV-1 RNAs should be performed by the Abbott RealTime HIV-1 assay at any laboratory that possesses a CLIA certification or its equivalent. Shipping should be done in an expeditious manner, preferably the day the sample is obtained.

HIV-1 Drug Resistance Genotyping
Resistance genotyping will be performed in those individuals with confirmed HIV-1 RNA >200 copies/mL on two consecutive assessments using the sample from the first of the two time points with HIV-1 RNA >200 copies/mL. Upon confirmation of virologic failure, a sample from the first time point having a viral load at least 1000 copies/mL will be sent for HIV-1 drug resistance genotyping.

6.3.8 Viral Reservoir Studies

Cryopreserved PBMCs for **Inducible RNA** Assay
The quantitative **inducible RNA** assay will be performed on resting CD4+ cells obtained from cryopreserved PBMC.

Cryopreserved PBMCs for Cell-Associated RNA and DNA
The cell-associated HIV-1 RNA and DNA will be performed on cryopreserved PBMC.

NOTE: Extraction, purification and quantification of cell-associated RNA and DNA will take place simultaneously on the same cryopreserved PBMC aliquot.

Stored Plasma for Residual Viremia by Single-Copy RNA Assay
Refer to the LPC for blood volumes.

6.3.9 Gene Expression Studies
Whole blood will be stored for gene expression assays.

6.3.10 Stored Samples for Future Unspecified Assays

Plasma **and PBMCs** will be collected, processed, and stored for future unspecified immunologic and exploratory aims, if the allowed maximum blood volume is not exceeded.

6.3.11 Procedures

Tuberculosis Test

Purified protein derivative tuberculin skin testing (TST) will be performed at screening for **participants** with either no prior or negative TB testing. **Participants** with prior positive TB screening tests do not need to have repeat TB testing. TST sites will be evaluated for induration between 48 and 72 hours after the PPD dose is administered. An induration of ≥5 mm will be considered positive. TB screening may also be performed by blood tests (also called interferon-gamma release assays or IGRAs), and is the screening of choice for individuals who have received Bacillus Calmette–Guérin (BCG) vaccine. TB results do not need to be recorded on a CRF.

Chest Radiograph

At screening (after pregnancy test) a posterior-anterior (PA) and lateral chest x-ray will be performed and reviewed by site staff for evidence of lung disease including active or latent tuberculosis. Chest x-ray results do not need to be recorded on a CRF.

Anal Swabs/Anal Pap Test

Refer to the A5337 Anal Swab Instruction Sheet posted on the PSWP for processing and shipping information.

Anal swabs will be collected for HPV DNA quantification. Specimens will be frozen and stored.

Anal Pap tests will be performed at a laboratory that has CLIA certification or its equivalent and the result will be recorded on a CRF. No specimens need to be collected if anal Pap tests were done as part of routine medical care outside the study ±4 weeks of the scheduled study visit and tested in a CLIA certified laboratory, but results need to be recorded.

Oral Swabs

Oral swabs will be collected to evaluate whether the use of sirolimus is associated with more frequent reactivation or suppression of HHV. Refer to the A5337 LPC posted on the PSWP for processing and shipping information.

6.3.12 Pharmacokinetic Samples

PK Monitoring of Sirolimus

Trough sirolimus levels (whole blood) will be collected 4 days after administration of the first dose of sirolimus and will be analyzed and reported in real time. Trough samples will then be drawn twice a week (approximately days 4 and 7 of each week) for up to 4 weeks until two consecutive drug levels fall within 5-10 ng/mL. Once two consecutive levels fall within 5-10 ng/mL (which may be achieved before the 4-week time point after sirolimus is initiated), trough samples will be drawn every 4 weeks until study week 32 (see section 6.1.1).

The windows for PK sampling are ±1 day through week 16 and ±1 week for weeks 18 through 32.

Stored Plasma for PK Exposure Estimates of ARVs
In addition to real-time sirolimus testing, stored plasma will be collected for batched analysis of ARV drug concentrations.

PK Dosing Restrictions
Participants having PK sampling should be instructed to hold their morning doses of sirolimus and ARVs so a trough sample can be collected. Once trough samples have been collected, **participants** can take their normal morning doses of sirolimus and ARVs. For **participants** taking EFV, the evening dose can be taken and the time of the last dose recorded when **participants** come to the clinic for PK blood draws.

PK Data Collection
For sirolimus and ARV PK collection visits, food intake, and date, time and amount of the last three doses of sirolimus and ARVs will be recorded on the CRF.

7.0 CLINICAL MANAGEMENT ISSUES

Criteria for **participant** management, dose interruptions, modifications and discontinuation of treatment will be mandated only for toxicities attributable to sirolimus.

The grading system for drug toxicities is located in the Division of AIDS Table for Grading the Severity of Adult and Pediatric Adverse Events, **Version 2.0, November 2014**, which can be found on the DAIDS RSC Web site: http://rsc.tech-res.com/safetyandpharmacovigilance/.

NOTE: The protocol core team must be notified by e-mail at actg.corea5337@fstrf.org within 48 hours regarding toxicities that result in a change in study treatment (including permanent discontinuation) during the study-defined 20-week treatment period.

7.1 Toxicity

7.1.1 Grade 1 or 2 Toxicity

Participants who develop a Grade 1 or 2 AE or toxicity felt to be related to study drug may continue study treatment. If a **participant** chooses to discontinue study treatment, the site should notify the protocol core team at actg.corea5337@fstrf.org within 48 hours and follow the **participant** on study/off study treatment.

7.1.2 Grade 3 Toxicity

- If the investigator has compelling evidence that the AE has NOT been caused by the study drug, dosing may continue. **Participants** who develop a Grade 3 AE or toxicity, except as stated in the following sections, should have sirolimus

withheld, and the A5337 core team consulted. The **participant** should be reevaluated weekly until the AE returns to Grade $\leq$2, at which time the study drug may be reintroduced at the discretion of the investigator or according to standard practice.

- If the same Grade 3 AE recurs within 4 weeks, the study drug must be permanently discontinued if the investigator considers the AE related to study drug. However, if the same Grade 3 AE recurs after 4 weeks, the management scheme outlined above may be repeated.
- **Participants** experiencing Grade 3 AEs requiring permanent discontinuation of study drug should be followed weekly until resolution of the AE and should be encouraged to complete the early discontinuation evaluations and continue in on-study follow-up.

7.1.3 Grade 4 Toxicity

- **Participants** who develop a Grade 4 symptomatic AE or toxicity will have study drug permanently discontinued and should notify the protocol core team at actg.corea5337@fstrf.org within 48 hours. **Participants** experiencing Grade 4 AEs requiring permanent discontinuation of study drug should be followed weekly until resolution of the AE or return to baseline and encouraged to complete the early discontinuation evaluations and continue on-study follow-up.

- **Participant**s with Grade 4 asymptomatic laboratory abnormalities may continue study drug if the investigator has compelling evidence that the toxicity is NOT related to the study drug.

7.1.4 CD4+ Decrease

Study drug will be discontinued in **participants** who exhibit CD4+ cell count decrease to <300 cells/mm^3 or decreases >50% from study entry value on two consecutive assessments.

7.1.5 Detectable HIV-1 RNA

Study drug will be discontinued in **participants** who have HIV-1 RNA >200 copies/mL on two consecutive assessments.

7.1.6 Detectable EBV in Blood

Sirolimus will be discontinued if EBV DNA is detected.

7.1.7 CMV viremia

CMV viremia without evidence of end-organ involvement will not require study drug termination.

7.2 Management of Specific Toxicities

7.2.1 Hepatotoxicity

Liver function test (LFT) monitoring will be performed as detailed in section 6.0. In the event that there is an increase in ALT or AST to >5 x ULN, sirolimus will be stopped and LFT blood testing will be repeated every 2 weeks until the values have returned to normal range.

7.2.2 Pulmonary Toxicity

All potential **participants** will have a chest x-ray and pulse oximetry measured prior to starting sirolimus therapy. Respiratory symptoms such as shortness of breath or coughing will be monitored at each study visit. If **participants** develop serious unexplained cough or shortness of breath suggestive of interstitial lung disease, they will be told not to take the next dose of study medication. An additional evaluation should be performed at the discretion of the site investigator, which may include pulse oximetry, physical examination, imaging including chest x-ray, chest computed tomography (CT scan) and possible referral to a pulmonologist or other specialist.

NOTE: Sirolimus will be held for Grade 2 dyspnea. At the discretion of the site investigator, in consultation with the protocol team, study treatment may be withheld or discontinued for Grade 1 pulmonary toxicity. Once the dyspnea has resolved sirolimus may be restarted with caution. If dyspnea recurs, sirolimus must be discontinued.

7.2.3 Myelosuppression

Sirolimus will be held for neutrophil toxicity (ANC <1000 cells/μL) and thrombocytopenia (<100,000/mm^3) until repeat testing confirms these results. At the discretion of the site investigator and in consultation with the protocol team, study treatment may be permanently discontinued.

7.2.4 Nephrotoxicity

Any **participant** with a calculated CrCl <50 mL/min or a reduction in CrCl by 30% or more should have the value confirmed within 7 days. Sirolimus will be discontinued if confirmed calculated CrCl decreases by >30% after initiation of treatment or falls below 50 mL/min/1.73 m^2.

Urine protein to creatinine ratios will also be checked per the SOE. Sirolimus will be discontinued if the ratio is >1 **g/g**.

7.2.5 Hypertriglyceridemia/Hyperlipidemia

Elevated triglycerides or lipid levels Grade ≥3 from a non-fasting blood draw should be repeated after an 8-hour fast. Only levels done in a fasting state should be used

to determine toxicity management. **Participants** with asymptomatic Grade <3 triglyceride, total cholesterol, or LDL cholesterol elevations may continue study drugs, at the discretion of the site investigator. See sections 7.1.2 and 7.1.3 for management of Grade 3 and 4 toxicities.

7.3 Management of HPV-Related Disease

Management of anal HPV-related disease is at the discretion of the **participant's** primary care physician, but if at all possible, treatment of anal dysplasia should be delayed until the end of the study.

7.4 Pregnancy Outcomes and Obstetrical History Reporting

Female **participants** who become pregnant while on study must discontinue study medication and study evaluations, but will be encouraged to remain on study so that pregnancy outcome data can be collected. Women will remain on study and complete the premature study discontinuation visit 1 week after stopping treatment; the off-study CRF must be completed at the end of the pregnancy. The outcome and the AEs for the participant and infant will be recorded on an outcome CRF. If a woman does not wish to remain on study follow-up at the time of study discontinuation, site staff should request permission to contact her regarding pregnancy and infant outcomes at the end of pregnancy. If the information is obtained, pregnancy outcomes for the participant and infant will be submitted.

Pregnancies that occur on study in participants receiving ART should be reported by the CRS to the Antiretroviral Pregnancy Registry. More information is available at www.apregistry.com. Phone: 800-258-4263; Fax: 800-800-1052.

8.0 CRITERIA FOR DISCONTINUATION

8.1 Permanent and Premature Treatment Discontinuation

- Drug-related toxicity (see section 7.1 and 7.2).
- Requirement for prohibited concomitant medications (see section 5.4).
- Completion of treatment as defined in the protocol.
- Pregnancy or breastfeeding.
- Request by **participant** to terminate treatment.
- Clinical reasons believed life threatening by the physician, even if not addressed in the toxicity section of the protocol.
- Any two consecutive CD4+ cell counts <300 mm^3 or >50% decrease from study entry value.
- Any two consecutive HIV-1 RNA levels >200 copies/mL.
- **Participant** repeatedly noncompliant missing >3 doses of study drug a week for 2 or more weeks.
- **Participant** misses two consecutive PK monitoring blood draws.

- **Participant** misses two consecutive clinic visits.

8.2 Premature Study Discontinuation

- ART is permanently discontinued.
- Request by the **participant** to withdraw.
- Request of the primary care provider if s/he thinks the study is no longer in the best interest of the **participant**.
- **Participant** judged by the investigator to be at significant risk of failing to comply with the provisions of the protocol as to cause harm to self or seriously interfere with the validity of the study results.
- At the discretion of the IRB, NIAID, Office for Human Research Protections (OHRP), and other government agencies as part of their duties to ensure that research **participants** are protected or the industry supporter or its designee.

9.0 STATISTICAL CONSIDERATIONS

9.1 General Design Issues

A5337 is a phase I/II open-label, single-arm, pilot study to evaluate the safety, immunologic, virologic and pharmacologic effects of sirolimus in HIV-infected participants receiving effective ART who have HIV-1 RNA <40 copies/mL and CD4+ T-cell counts ≥**350** cells/mm^3. Each **participant** will receive 20 weeks of sirolimus starting 12 weeks after study entry, followed by an additional 12 weeks off sirolimus, for a total duration of 44 weeks. Sirolimus will be dose-adjusted based on frequent measurements of sirolimus drug levels to achieve desired target levels. Accrual of the targeted 30 **participants** is anticipated to take 8-12 months. Note that the primary outcome measures are through week 32 while the study has an additional 12 weeks of post-treatment follow-up.

9.2 Outcome Measures

9.2.1 Primary Outcome Measures

9.2.1.1 Safety

Occurrence of a new Grade ≥3 AE, including signs/symptoms, lab toxicity or clinical event, that is definitely, probably or possibly related to study treatment, as judged by the core team, or by change (confirmed >50% decline or to <300 cells/mm^3) in CD4+ cell counts while on sirolimus.

9.2.1.2 Efficacy – Immunologic

Frequency of HIV-1 Gag-specific CD8+ cells by intracellular staining for IFN-gamma at baseline and at week 32 (20 weeks on sirolimus).

9.2.1.3 Efficacy – Virologic

CD4+ T-cell-associated HIV-1 RNA and plasma HIV-1 RNA by SCA at baseline and at week 32 (20 weeks on sirolumus)

9.2.2 Secondary Outcome Measures

9.2.2.1 CD4+ cell counts at baseline and at weeks 14, 16, 20, 24, 32 (2, 4, 8, 12, 20 weeks on sirolimus) and 44

9.2.2.2 HIV-1 RNA levels by conventional assay at baseline and at weeks 16, 20, 24, 32 (4, 8, 12, 20 weeks on sirolimus) and 44

9.2.2.3 HIV-1-specific CD4+ cell responses and HIV-1-specific CD8+ cell responses (other than gag) at baseline and at weeks 13, 16, 24, 32 (1, 4, 12, 20 weeks on sirolimus) and 44

9.2.2.4 T-cell activation and proliferation (% CD4+ and CD8+ cells CD38+/HLA-DR+, CD25+, PD-1+, Ki67+ and PD-L1 expression) at baseline and at weeks 13, 16, 24, 32 (1, 4, 12, 20 weeks on sirolimus) and 44

9.2.2.5 HIV-1 DNA levels in CD4+ cells at baseline and at weeks 13, 16, 24, 32 (1, 4, 12, 20 weeks on sirolimus) and 44

9.2.3 Exploratory Outcome Measures

9.2.3.1 Ex vivo flow cytometric phenotyping of activation/proliferation in CD4 and CD8 central memory (CM), transitional memory (TM), effector memory (EM) and naïve (N) populations as defined by CCR7 and CD27 expression

9.2.3.2 Downstream signaling of homeostatic proliferation-regulating cytokines, such as IL-7 and IL-15, in CD4 and CD8 memory subsets and correlate preferential induction of phosphorylation of STAT-5 with changes in reservoir size

9.2.3.3 Soluble markers including IL-6, IL-7 and IL-15

9.2.3.4 HIV-1 specific antibody levels, the breadth of antibody responses and B-cell phenotype, rearrangements and class switching

9.2.3.5 HPV typing and quantification of HPV DNA from rectal swabs at week 12 (baseline) and week 32 (20 weeks on sirolimus) and pathologic evaluation for anal dysplasia from anal Pap tests prior to and after sirolimus administration.

9.2.3.6 Pharmacokinetic exposure of sirolimus and ARVs in the context of concomitant use

9.2.3.7 Measurements of CMV, EBV, HSV and HHV in oral swabs prior to and after sirolimus administration

9.3 Randomization and Stratification

There is no randomization in this study. Eligible **participants** will be registered at study entry and stratified by ART regimen which determines initial dose of treatment**.**

9.4 Sample Size and Accrual

Regarding the assessment of safety/toxicity (see section 9.2.1.1), the sample size of 30 sirolimus-treated **participants** will provide >90% probability of observing a sirolimus-related adverse event that would occur in 8% or more of treated **participants.**

The primary efficacy analyses will evaluate paired baseline and post-sirolimus therapy immunologic and virologic measurements to detect changes; these measures are assumed stable on long-term suppressive ART (see section 2.2.6). Allowing 17% sample size adjustment for lost to follow-up and treatment discontinuation for the planned as-treated analyses (i.e., n=25 **participants** assumed evaluable), the study will have 80% power to detect a 0.59 standard deviation (SD) effect size based on a two-sided, alpha=0.05, 1-arm t-test. This effect size translates (under assumed normality) into a probability of 0.72 that a **participant** receiving sirolimus will have an observed increase in HIV-specific CD8 immunity (see section 9.2.1.2). Similarly, power would be 80% to detect a corresponding decrease in cell-associated RNA (see section 9.2.1.3). The null hypothesis is that the probability is 0.5 (equally likely to observe increase or decrease). For residual viremia (SCA, see section 9.2.1.3), effect size is slightly larger due to anticipated left-censoring below assay limits. From numerical simulation based on a 1-arm t-test (imputing half the lower limit for SCA results below assay limits), power is estimated to be 80% to detect an underlying effect size of 0.67 SD; this 80% power corresponds to assuming that the probability is 0.5 (50%) for a baseline (pre-entry, entry) SCA to be below assay limit compared to 0.75 (75%) after treatment. The assumption that 50% of SCA results will be below assay limits at baseline is supported by findings from ACTG A5276s in which 56% of 668 SCA measurements after 192 and 208 weeks of suppressive ART on 334 **participants** were below assay limits (<1 copy/mL). Analyses will also jointly evaluate efficacy measurements at the multiple post-treatment time points (e.g., the change from baseline to the average of the weeks 16, 24, and 32 measurements [4, 12, 20 weeks on sirolimus]), which could increase statistical power.

9.5 Monitoring

Accrual, baseline characteristics, conduct of the study (including premature treatment and study discontinuations, and reasons, and summaries of sirolimus dosages and trough levels), interruptions of ART >2 days, virologic failures (defined as HIV-1 RNA >200 copies/mL on two consecutive assessments), two consecutive CD4 counts below 300 cells/mm^3 (or >50% decrease from study entry) and all reported toxicities and events will be monitored during the study with reports sent to the core team on a regular basis. In addition,

assessment of the availability of stored samples for planned assays will be reviewed regularly. The core protocol team will review the individual safety data frequently (at least monthly) to assess relation of all reported toxicities and AEs to study treatment, including in this assessment the site investigator's opinion on their relation to study treatment as reported on the case report forms.

At the earlier of a) approximately 6 months after the first **participant** begins study treatment and b) after 12 **participants** have reached 12 weeks of study treatment, a Study Monitoring Committee (SMC) will be convened to review the study data and progress according to ACTG Standard Operating Procedures. The SMC will review accrual, **participant** characteristics, AE summaries (and listings detailing each team decision as to the relationship of the AE to study treatment), sirolimus dosages and trough levels (in particular for any **participant** with a Grade 3 or higher AE), CD4+ cell counts and HIV-1 RNA levels/suppression over time, off-treatment and off-study rates (and reasons) and completeness of sample availability. In addition, sirolimus use in all **participants** will be stopped and an SMC safety review will be promptly convened if there are any Grade 4 AEs or greater than three Grade 3 AEs attributed to study treatment in two or more **participants** per section 9.2.1.1. An SMC review will also be triggered if more than 25% of **participants** (evaluating at least 12 **participants**, e.g., greater than 3 of 12) discontinue study treatment due to AEs or other side effects attributable to study drug; the therapeutic duration of the trial might be reduced to 12 weeks or otherwise modified following SMC review. Subsequent SMC reviews will be approximately every 6 months. In addition, the core team or the SMC, at any time it thinks appropriate, may ask for the SMC to independently review all available safety data.

9.6 Analyses

Baseline will defined as the average of entry and week 12 measurements (or will be based on one if the other is missing).

9.6.1 Primary Analyses

For the primary safety analysis, ≥Grade 3 AEs attributed to study treatment and confirmed CD4+ declines (see section 9.2.1.1) will be summarized. In addition, all other reported AEs ≤Grade 2 attributed to study treatment as well as all reported events ≥Grade 2 (regardless of treatment relatedness) will be summarized. All **participant**s who have been exposed to study treatment will be included in the analysis.

For the primary immunologic (see section 9.2.1.2) and virologic (see section 9.2.1.3) efficacy outcomes, post-treatment changes will be evaluated comparing measurements pre-treatment (averaged) to the measurement 20 weeks after starting study treatment, testing the null hypothesis of no difference pre-treatment versus post-treatment using a paired t-test. Log-transformations may be applied for the virologic outcomes. To address anticipated left-censoring of SCA measurements, a value one half the lower assay limit will be imputed. If feasible with the modest sample size, censored-data longitudinal data methods that statistically address the

issue of left-censoring (SCA measurements below assay limits) will also be used to evaluate and estimate treatment effects **[59].** Mean and quartile plots will summarize the immunologic and virologic measurements at each visit. Because the aim of this pilot study is to investigate the biologic effects of sirolimus, the analyses will be as-treated, limited to **participants** who have data at baseline and week 32 (20 weeks on sirolimus) and who remained on study treatment and ART (and without virologic failure) through week 32. A supplemental analysis will be based on changes from baseline to the last available time point for a **participant** while on study treatment and ART (and without virologic failure).

If there is evidence that there is a significant difference between the week 0 and week 12 levels of the primary immunologic and virologic efficacy measurements, based on paired t-tests between these two time points, then the primary analysis will instead be based on a comparison of slopes pre- versus post-treatment (see 9.6.2). Both sets of results, from the paired t-test analyses and the comparison of slopes analyses, will be reported in the presentation of the study findings.

9.6.2 Secondary Analyses

Additional analyses of safety will summarize the magnitude of CD4+ cell changes baseline to post-treatment time points (mean, quartiles). Analyses will also summarize the number of **participants** who exhibit on two consecutive measurements HIV RNA >200 copies/mL on conventional assay. Tolerability will be assessed by the number of **participants** who prematurely discontinue study treatment and the reasons for discontinuation.

Analyses of secondary virologic and immunologic outcomes will parallel the primary analyses outlined in section 9.6.1. Graphical approaches will summarize the virologic and immunologic measurements over time. Supplemental analyses will compare the change in the slope-over-time pre-treatment (pre-entry to start of treatment) versus post-treatment (start of treatment through week 20 while on treatment) and also may examine the change from baseline to the average of the week 16, 24 and 32 (4, 12, 20 weeks on sirolimus) measurements. Soluble markers (e.g., IL-6 levels) will be log-transformed.

Analyses will also summarize changes in immunologic and virologic measures after discontinuation of study treatment.

Associations between changes in the various immunologic and virologic measurements will be evaluated using scatterplots and correlations coefficients.

The relationship between anal HPV, particularly HPV 16, and immunological measurements of activation and virological measurements of HIV reservoir size will be correlated.

The effect of study treatment on anal dysplasia will be described by evaluating the change in anal Pap tests after study therapy. Pap tests will be categorized as

normal or abnormal according to standard clinical criteria.

10.0 PHARMACOLOGY PLAN

10.1 Pharmacology Study Design

Pharmacology Objective: To assess the pharmacokinetic (PK) interactions between sirolimus therapy and ARV drug levels.

Sirolimus trough drug levels will be drawn on **participants** 4 days after the initial dose until two consecutive sirolimus levels are within the goal range of 5-10 ng/mL. Once two consecutive levels fall within 5-10 ng/mL (which may be achieved before the 4-week time point after sirolimus is initiated), trough samples will be drawn every 4 weeks until week 32 (see section 6.1.1). Monitoring will be done by individual sites that have or use a laboratory that has a CLIA certification or its equivalent. For safety and ease of dosing, **participants** will be recruited and followed only at medical centers that have rapid sirolimus testing available or are able to ship samples to Quest laboratory for rapid turnaround of results based on liquid chromatography tandem mass spectrometry analysis which enables accurate and consistent measures of levels across study sites. Results should be available within 72 hours if shipped to Quest or local laboratory. Sirolimus dose will not be adjusted until study drug has been taken for a minimum of 1 week.

In addition, samples will be collected and stored for batched analysis of ARV measurements following study completion. ARV trough levels will be collected just prior to initiating sirolimus to serve as baseline evaluations. These samples will be collected just prior to morning dosing. **Participants** will be asked to hold their morning doses to allow trough collections.

10.2 Primary and Secondary Data, Modeling and Data Analysis

Real-time analysis for sirolimus will be used for **participant** monitoring only. Batched sample analysis of ARVs will be performed post-hoc using standardized analytical methods. This monitoring will allow the assessment of the PK of sirolimus therapy on ARV drug levels and evaluate the effects of ARVs on sirolimus levels. These analyses will inform potential required dose adjustments if further clinical trials are planned. However, these levels will not be used to dictate dose adjustment or clinical changes.

Batched analysis of ARVs will be carried out using LC tandem MS. As **participants** will be taking various non-PI ARVs, analysis for ARVs will focus on the most common ARVs prescribed to optimize comparisons (e.g., efavirenz, tenofovir, integrase inhibitors). ARV levels will be compared prior to and following sirolimus initiation and to historical ARV PK data. Sirolimus levels will be compared across ARV regimens and to historical data, if available.

11.0 DATA COLLECTION AND MONITORING AND ADVERSE EVENT REPORTING

11.1 Records to Be Kept

Case report forms (CRFs) will be provided for each **participan**t. **Participants** must not be identified by name on any CRFs. **Participants** will be identified by the patient identification number (PID) and study identification number (SID) provided by the ACTG DMC upon registration.

11.2 Role of Data Management

11.2.1 Instructions concerning the recording of study data on CRFs will be provided by the ACTG DMC. Each CRS is responsible for keying the data in a timely fashion.

11.2.2 It is the responsibility of the ACTG DMC to assure the quality of computerized data for each ACTG study. This role extends from protocol development to generation of the final study databases.

11.3 Clinical Site Monitoring and Record Availability

11.3.1 Site monitors under contract to the NIAID will visit participating clinical research sites to review the individual **participant** records, including consent forms, CRFs, supporting data, laboratory specimen records, and medical records (physicians' progress notes, nurses' notes, **participants'** hospital charts), to ensure protection of study **participants**, compliance with the protocol and accuracy and completeness of records. The monitors also will inspect sites' regulatory files to ensure that regulatory requirements are being followed and sites' pharmacies to review product storage and management.

11.3.2 The site investigator will make study documents (e.g., consent forms, drug distribution forms, CRFs) and pertinent hospital or clinic records readily available for inspection by the local IRB, the site monitors, industry supporter, the NIAID and the OHRP, for confirmation of the study data.

11.4 Expedited Adverse Event Reporting to DAIDS

11.4.1 Adverse Event Reporting to DAIDS

Requirements, definitions and methods for expedited reporting of Adverse Events (AEs) are outlined in Version 2.0 of the DAIDS EAE Manual, which is available on the RSC website at http://rsc.tech-res.com/safetyandpharmacovigilance/.

The DAIDS Adverse Events Reporting System (DAERS), an internet-based reporting system, must be used for expedited AE reporting to DAIDS. In the event of system outages or technical difficulties, expedited AEs may be submitted via the DAIDS EAE Form. For questions about DAERS, please contact **NIAID CRMS Support** at

CRMSSuport@niaid.nih.gov. Site queries may also be sent from within the DAERS application itself.

Sites where DAERS has not been implemented will submit expedited AEs by documenting the information on the current DAIDS EAE Form. This form is available on the RSC website: http://rsc.tech-res.com/safetyandpharmacovigilance/. For questions about EAE reporting, please contact the RSC (DAIDSRSCSafetyOffice@tech-res.com).

11.4.2 Reporting Requirements for this Study

The SAE Reporting Category, as defined in Version 2.0 of the DAIDS EAE Manual, will be used for this study from the time of enrollment through week 44. After this time, the SUSAR Reporting Category will be used.

The study agent for which expedited reporting is required is sirolimus.

All cancers except basal cell cancer of the skin are to be reported as expedited events.

11.4.3 Grading Severity of Events

The Division of AIDS Table for Grading the Severity of Adult and Pediatric Adverse Events (DAIDS AE Grading Table), **Version 2.0, November 2014**, must be used and is available on the DAIDS RSC Web site at http://rsc.tech-res.com/safetyandpharmacovigilance/.

11.4.4 Expedited AE Reporting Period

The EAE reporting period for this study is the entire study duration for an individual participant (from study enrollment until study completion or discontinuation of the participant from study participation for any reason).

After the protocol-defined AE reporting period, unless otherwise noted, only suspected unexpected serious adverse reactions (SUSARs) as defined in Version 2.0 of the EAE Manual will be reported to DAIDS if the study staff become aware of the events on a passive basis (e.g., from publicly available information).

12.0 PARTICIPANTS

12.1 Institutional Review Board (IRB) Review and Informed Consent

This protocol, the informed consent document (Appendix I) and any subsequent modifications will be reviewed and approved by the IRB responsible for oversight of the study. A signed consent form will be obtained from the **participant** (or legal guardian or person with power of attorney for **participants** who cannot consent for themselves). The

consent form will describe the purpose of the study, the procedures to be followed, and the risks and benefits of participation. A copy of the consent form will be given to the **participant** or legal guardian and this fact will be documented in the **participant's** record.

12.2 **Participant** Confidentiality

All laboratory specimens, evaluation forms, reports, and other records that leave the site will be identified by coded number only to maintain **participant** confidentiality. All records will be kept locked. All computer entry and networking programs will be done with coded numbers only. Clinical information will not be released without written permission of the **participant**, except as necessary for monitoring by the ACTG, IRB, NIAID, OHRP, other government agencies as part of their duties to ensure that research **participants** are protected and industry supporter or designee.

12.3 Study Discontinuation

The study may be discontinued at any time by the ACTG, IRB, NIAID, industry supporter, OHRP, or other government agencies as part of their duties to ensure that research **participants** are protected.

13.0 PUBLICATION OF RESEARCH FINDINGS

Publication of the results of this trial will be governed by ACTG policies. Any presentation, abstract, or manuscript will be made available for review by the industry supporter prior to submission.

14.0 BIOHAZARD CONTAINMENT

As the transmission of HIV and other blood-borne pathogens can occur through contact with contaminated needles, blood, and blood products, appropriate blood and secretion precautions will be employed by all personnel in the drawing of blood and shipping and handling of all specimens for this study, as currently recommended by the Centers for Disease Control and Prevention and the National Institutes of Health.

All dangerous goods and materials, including diagnostic specimens and infectious substances, must be transported using packaging mandated by CFR 42 Part 72. Please refer to instructions detailed in the International Air Transport Association (IATA) Dangerous Goods Regulations.

15.0 REFERENCES

1. Deeks SG, Autran B, Berkhout B, et al. Towards an HIV cure: a global scientific strategy. Nat Rev Immunol 2012;12:607-14.
2. Isotani S, Hara K, Tokunaga C, Inoue H, Avruch J, Yonezawa K. Immunopurified mammalian target of rapamycin phosphorylates and activates p70 S6 kinase alpha in vitro. J Biol Chem 1999;274:34493-8.
3. Gamper CJ, Powell JD. All PI3Kinase signaling is not mTOR: dissecting mTOR-dependent and independent signaling pathways in T cells. Front Immunol 2012;3:312.
4. Battaglia M, Stabilini A, Roncarolo MG. Rapamycin selectively expands CD4+CD25+FoxP3+ regulatory T cells. Blood 2005;105:4743-8.
5. Wiederrecht GJ, Sabers CJ, Brunn GJ, Martin MM, Dumont FJ, Abraham RT. Mechanism of action of rapamycin: new insights into the regulation of G1-phase progression in eukaryotic cells. Prog Cell Cycle Res 1995;1:53-71.
6. Keating R, Hertz T, Wehenkel M, et al. The kinase mTOR modulates the antibody response to provide cross-protective immunity to lethal infection with influenza virus. Nat Immunol 2013;14:1266-76.
7. **Highlights of prescribing information for rapamune (sirolimus) oral solution and tablets, initial U.S. approval 1999:** http://www.accessdata.fda.gov/drugsatfda_docs/label/2015/021083s058,021110s075lbl.pdf
8. Ponticelli C. The pros and the cons of mTOR inhibitors in kidney transplantation. Expert Rev Clin Immunol 2014;10:295-305.
9. Pidala J, Kim J, Jim H, et al. A randomized phase II study to evaluate tacrolimus in combination with sirolimus or methotrexate after allogeneic hematopoietic cell transplantation. Haematologica 2012;97:1882-9.
10. Kubiak DW, Koo S, Hammond SP, et al. Safety of posaconazole and sirolimus coadministration in allogeneic hematopoietic stem cell transplants. Biol Blood Marrow Transplant 2012;18:1462-5.
11. Rosenbeck LL, Kiel PJ, Kalsekar I, et al. Prophylaxis with sirolimus and tacrolimus +/- antithymocyte globulin reduces the risk of acute graft-versus-host disease without an overall survival benefit following allogeneic stem cell transplantation. Biol Blood Marrow Transplant 2011;17:916-22.
12. Cutler C, Antin JH. Sirolimus immunosuppression for graft-versus-host disease prophylaxis and therapy: an update. Curr Opin Hematol 2010;17:500-4.
13. Ho VT, Aldridge J, Kim HT, et al. Comparison of Tacrolimus and Sirolimus (Tac/Sir) versus Tacrolimus, Sirolimus, and mini-methotrexate (Tac/Sir/MTX) as acute graft-versus-host disease prophylaxis after reduced-intensity conditioning allogeneic peripheral blood stem cell transplantation. Biol Blood Marrow Transplant 2009;15:844-50.

14. Krown SE, Roy D, Lee JY, et al. Rapamycin with antiretroviral therapy in AIDS-associated Kaposi sarcoma: an AIDS Malignancy Consortium study. J Acquir Immune Defic Syndr 2012;59:447-54.

15. Di Benedetto F, Di Sandro S, De Ruvo N, et al. First report on a series of HIV patients undergoing rapamycin monotherapy after liver transplantation. Transplantation 2010;89:733-8.

16. Stallone G, Schena A, Infante B, et al. Sirolimus for Kaposi's sarcoma in renal-transplant recipients. N Engl J Med 2005;352:1317-23.

17. Donia M, McCubrey JA, Bendtzen K, Nicoletti F. Potential use of rapamycin in HIV infection. Br J Clin Pharmacol 2010;70:784-93.

18. Oswald-Richter K, Grill SM, Leelawong M, Unutmaz D. HIV infection of primary human T cells is determined by tunable thresholds of T cell activation. Eur J Immunol 2004;34:1705-14.

19. Roy J, Paquette JS, Fortin JF, Tremblay MJ. The immunosuppressant rapamycin represses human immunodeficiency virus type 1 replication. Antimicrob Agents Chemother 2002;46:3447-55.

20. Gilliam BL, Heredia A, Devico A, et al. Rapamycin reduces CCR5 mRNA levels in macaques: potential applications in HIV-1 prevention and treatment. AIDS 2007;21:2108-10.

21. Heredia A, Amoroso A, Davis C, et al. Rapamycin causes down-regulation of CCR5 and accumulation of anti-HIV beta-chemokines: an approach to suppress R5 strains of HIV-1. Proc Natl Acad Sci U S A 2003;100:10411-6.

22. Mulampaka SN, Dixit NM. Estimating the threshold surface density of Gp120-CCR5 complexes necessary for HIV-1 envelope-mediated cell-cell fusion. PLoS One 2011;6:e19941.

23. Sieg SF, Rodriguez B, Asaad R, Jiang W, Bazdar DA, Lederman MM. Peripheral S-phase T cells in HIV disease have a central memory phenotype and rarely have evidence of recent T cell receptor engagement. J Infect Dis 2005;192:62-70.

24. Lederman MM, Calabrese L, Funderburg NT, et al. Immunologic failure despite suppressive antiretroviral therapy is related to activation and turnover of memory CD4 cells. J Infect Dis 2011;204:1217-26.

25. Henrich T, Hu Z, Li J, et al. Long-Term Reduction in Peripheral Blood HIV-1 Reservoirs Following Reduced-Intensity Conditioning Allogeneic Stem Cell Transplantation. Journal of Infectious Diseases 2013; 207:1694-1702.

26. Stock PG, Barin B, Murphy B, et al. Outcomes of kidney transplantation in HIV-infected recipients. N Engl J Med 2010;363:2004-14.

27. Samonakis DN, Cholongitas E, Triantos CK, et al. Sustained, spontaneous disappearance of serum HCV-RNA under immunosuppression after liver transplantation for HCV cirrhosis. J Hepatol 2005;43:1091-3.

28. Nashan B, Gaston R, Emery V, et al. Review of cytomegalovirus infection findings with mammalian target of rapamycin inhibitor-based immunosuppressive therapy in de novo renal transplant recipients. Transplantation 2012;93:1075-85.

29. Havenith SH, Yong SL, van Donselaar-van der Pant KA, van Lier RA, Ten Berge IJ, Bemelman FJ. Everolimus-Treated Renal Transplant Recipients Have a More Robust CMV-Specific CD8+ T-Cell Response Compared With Cyclosporine- or Mycophenolate-Treated Patients. Transplantation 2013;95:184-91.

30. Molinolo AA, Marsh C, El Dinali M, et al. mTOR as a molecular target in HPV-associated oral and cervical squamous carcinomas. Clin Cancer Res 2012;18:2558-68.

31. Tinker AV, Ellard S, Welch S, et al. Phase II study of temsirolimus (CCI-779) in women with recurrent, unresectable, locally advanced or metastatic carcinoma of the cervix. A trial of the NCIC Clinical Trials Group (NCIC CTG IND 199). Gynecol Oncol 2013;130:269-74.

32. Coppock JD, Wieking BG, Molinolo AA, Gutkind JS, Miskimins WK, Lee JH. Improved clearance during treatment of HPV-positive head and neck cancer through mTOR inhibition. Neoplasia 2013;15:620-30.

33. Mhatre M, McAndrew T, Carpenter C, Burk RD, Einstein MH, Herold BC. Cervical intraepithelial neoplasia is associated with genital tract mucosal inflammation. Sex Transm Dis 2012;39:591-7.

34. Rukasz D, Krajewska M, Augustyniak-Bartosik H, et al. Effective treatment of Kaposi sarcoma with everolimus in a patient with membranous glomerulonephritis. Intern Med J 2015;45:230-1.

35. Detroyer D, Deraedt K, Schoffski P, et al. Resolution of diffuse skin and systemic Kaposi's sarcoma in a renal transplant recipient after introduction of everolimus: a case report. Transpl Infect Dis 2015;17:303-7.

36. Krown SE, Roy D, Lee JY, et al. Rapamycin with antiretroviral therapy in AIDS-associated Kaposi sarcoma: an AIDS Malignancy Consortium study. J Acquir Immune Defic Syndr 2012;59:447-54.

37. Bissler JJ, McCormack FX, Young LR, et al. Sirolimus for angiomyolipoma in tuberous sclerosis complex or lymphangioleiomyomatosis. N Engl J Med 2008;358:140-51.

38. van den Eertwegh AJ, Karakiewicz P, Bavbek S, et al. Safety of Everolimus by Treatment Duration in Patients With Advanced Renal Cell Cancer in an Expanded Access Program. Urology 2013;81:143-9.

39. Huyghe E, Zairi A, Nohra J, Kamar N, Plante P, Rostaing L. Gonadal impact of target of rapamycin inhibitors (sirolimus and everolimus) in male patients: an overview. Transpl Int 2007;20:305-11.

40. Boobes Y, Bernieh B, Saadi H, Raafat Al Hakim M, Abouchacra S. Gonadal dysfunction and infertility in kidney transplant patients receiving sirolimus. Int Urol Nephrol 2010;42:493-8.

41. Bererhi L, Flamant M, Martinez F, Karras A, Thervet E, Legendre C. Rapamycin-induced oligospermia. Transplantation 2003;76:885-6.

42. Skrzypek J, Krause W. Azoospermia in a renal transplant recipient during sirolimus (rapamycin) treatment. Andrologia 2007;39:198-9.

43. Lee YR, Yang IH, Lee YH, et al. Cyclosporin A and tacrolimus, but not rapamycin, inhibit

MHC-restricted antigen presentation pathways in dendritic cells. Blood 2005;105:3951-5.

44. Araki K, Turner AP, Shaffer VO, et al. mTOR regulates memory CD8 T-cell differentiation. Nature 2009;460:108-12.

45. Baan CC, van der Mast BJ, Klepper M, et al. Differential effect of calcineurin inhibitors, anti-CD25 antibodies and rapamycin on the induction of FOXP3 in human T cells. Transplantation 2005;80:110-7.

46. Coenen JJ, Koenen HJ, van Rijssen E, Hilbrands LB, Joosten I. Rapamycin, and not cyclosporin A, preserves the highly suppressive CD27+ subset of human CD4+CD25+ regulatory T cells. Blood 2006;107:1018-23.

47. Dyavar Shetty R, Velu V, Titanji K, et al. PD-1 blockade during chronic SIV infection reduces hyperimmune activation and microbial translocation in rhesus macaques. J Clin Invest 2012;122:1712-6.

48. Wagner TA; Proliferation of Cells With HIV Integrated Into Regulatory Genes Is a Mechanism of Persistence, CROI 2014, http://www.sciencemag.org/content/343/6176/1188.full

49. Maldarelli F, Wu X, Su L, et al. HIV latency: Specific HIV integration sites are linked to clonal expansion and persistence of infected cells. Science 2014 Jul 11;345 (6193):179-83. doi:10.1126/science. 1254194. Epub 2014 Jun 26.

50. Velu V, Titanji K, Zhu B, et al. Enhancing SIV-specific immunity in vivo by PD-1 blockade. Nature 2009;458:206-10.

51. Haidinger M, Poglitsch M, Geyeregger R, et al. A versatile role of mammalian target of rapamycin in human dendritic cell function and differentiation. J Immunol 2010;185:3919-31.

52. Malat GE, Ranganna KM, Sikalas N, Liu L, Jindal RM, Doyle A. High frequency of rejections in HIV-positive recipients of kidney transplantation: a single center prospective trial. Transplantation 2012;94:1020-4.

53. Stock PG, Barin B, Hatano H, Rogers RL, Roland ME, Lee TH, Busch M, Deeks SG; for Solid Organ Transplantation in HIV Study Investigators. Reduction of HIVpersistence following transplantation in HIV-infected kidney transplant recipients. Am J Transplant. 2014 Apr 3. doi: 10.1111/ajt.12699. [Epub ahead of print]

54. Marfo K, Greenstein S. Antiretroviral and immunosuppressive drug-drug interactions in human immunodeficiency virus-infected liver and kidney transplant recipients. Transplant Proc 2009;41:3796-9.

55. Jain AK, Venkataramanan R, Fridell JA, et al. Nelfinavir, a protease inhibitor, increases sirolimus levels in a liver transplantation patient: a case report. Liver Transpl 2002;8:838-40.

56. Casazza JP, Betts MR, Picker LJ, Koup RA. Decay kinetics of human immunodeficiency virus-specific CD8+ T cells in peripheral blood after initiation of highly active antiretroviral therapy. J Virol 2001;75:6508-16.

57. Palmer S, Maldarelli F, Wiegand A, et al. Low-level viremia persists for at least 7 years in patients on suppressive antiretroviral therapy. Proc Natl Acad Sci U S A 2008;105:3879-84.

58. Gandhi RT, Zheng L, Bosch RJ, et al. The effect of raltegravir intensification on low-level residual viremia in HIV-infected patients on antiretroviral therapy: a randomized controlled trial. PLoS Med 2010;7.

59. Hughes JP. Mixed effects models with censored data with application to HIV RNA levels. Biometrics 1999;55:625-9.

APPENDIX I
SAMPLE INFORMED CONSENT

DIVISION OF AIDS
AIDS CLINICAL TRIALS GROUP (ACTG)
SAMPLE INFORMED CONSENT
For protocol A5337

Safety and Efficacy of Sirolimus for HIV Reservoir Reduction in Individuals on Suppressive Antiretroviral Therapy

SHORT TITLE FOR THE STUDY: (A5337) Sirolimus Study

INTRODUCTION

When a person becomes infected with HIV (the virus that causes AIDS), his/her immune system (the system that helps fight infection) is weakened (partly because the number of CD4+ cells goes down). Despite successful treatment with antiretroviral therapy (ART), latent reservoirs (infected cells that are not actively producing HIV) remain present in the blood and contribute to ongoing immune system activation and inflammation in the body. Ongoing inflammation may contribute to HIV persisting in cells in your body even though you are taking medications to treat HIV.

You are being asked to take part in this research study because you:

- are infected with HIV
- have been on continuous anti-HIV medicines with no detectable HIV for 24 months or longer
- have a CD4+ cell count of **350** or greater

This study is sponsored by the National Institutes of Health (NIH). The doctor in charge of this study at this site is: (insert name of Principal Investigator). Before you decide if you want to be a part of this study, we want you to know about the study.

This is a consent form. It gives you information about this study. The study staff will talk with you about this information. You are free to ask questions about this study at any time. If you agree to take part in this study, you will be asked to sign this consent form. You will get a copy to keep.

WHY IS THIS STUDY BEING DONE?

The purpose of this study is to find out about the safety of sirolimus in individuals with HIV infection who are also being treated with ART. We want to learn whether sirolimus will decrease inflammation and immune activation in the body, whether sirolimus will change the level of HIV in your blood, and how sirolimus interacts with ART in the blood. Sirolimus is approved by the Food and Drug Administration (FDA) to prevent organ rejection in patients aged 13 years and older receiving kidney transplants. Sirolimus has also been used for the prevention of

complications after stem cell transplants and as a treatment for certain kinds of cancers in HIV-infected patients.

WHAT DO I HAVE TO DO IF I AM IN THIS STUDY?

If you decide to take part in this research study, you will be asked to sign this consent form and schedule a screening visit to determine if you can join the study. If you enter the study, you will have laboratory and clinical evaluations performed and then be followed for 12 weeks before you start taking sirolimus. The reason for the 12-week delay in starting sirolimus is to provide an estimate of the level of HIV-1 in your body while you are taking your anti-HIV medications. After this, you will be seen in the clinic about 15 times.

Screening visit

- Your HIV infection will be confirmed. If there is no record available, you will have another HIV test. You may have to sign a separate consent form before having this test. A separate consent form may be given to you by the staff at your site.
- You will have a physical exam and will be asked about your health and medicines you have taken in the past or are taking now.
- You will have blood drawn for routine safety blood tests and to measure your CD4+ cell count (cells that fight infection) and HIV-1 viral load (the amount of virus in your blood). You will be given the test results when they are available.
- You will have blood drawn to test for hepatitis and for the Epstein-Barr virus (EBV), a virus of the herpes family. You will be given the test results when they are available.
- If you are a woman able to become pregnant, you will be asked to give a urine or blood sample to see if you are pregnant. You will not be able to enroll in this study if you are pregnant. You will be told the result of the test when it becomes available.
- You will be asked to provide a urine sample.
- You will have a skin test or blood test to test for tuberculosis unless you have had a positive tuberculosis test in the past. If you have the skin test, you will get a small shot that contains purified protein derivative (PPD). The needle is gently placed under the top layer of skin, causing a bump (welt) to form. This usually goes away in a few hours. After 48-72 hours, you must return to have the area checked to see if you have had a strong reaction to the test.
- You will have a chest x-ray.

If you do not enroll into the study

If you decide not to take part in this study or if you do not meet the eligibility requirements, we will still use some of your information. As part of this screening visit, some demographic (for example, age, sex, race), clinical (for example, disease condition, diagnosis), and laboratory (for example, CD4+ cell count, viral load) information is being collected from you so that ACTG researchers may help determine whether there are patterns or common reasons why people do not join a study.

Entry visit

If you qualify for the study, you will come to the clinic after the screening visit for the first entry visit. You will have a brief physical exam and be asked about your medical history and any medicines you have taken. You will have blood drawn for routine tests, CD4+ cell count, viral

load, and for samples to be stored for future testing for this study. If you are a woman able to become pregnant, you will be asked to give a urine or blood sample to see if you are pregnant. You will not be able to enroll in this study if you are pregnant. You will be told the result of the test when it becomes available.

A swab of your mouth will be taken that you can do yourself. You should not drink hot beverages (e.g., tea or coffee) 1 hour before you collect the sample. You will be given a cotton swab and asked to rub the tip repeatedly over the inside of both cheeks, along the upper and lower gum lines, inside and outside of the teeth, around the hard palate and across the soft palate (or roof of your mouth), and with the back of your throat (back wall) as you can tolerate.

Week 12 post-entry visit
Approximately 12 weeks after entry, you will come in for a visit before starting sirolimus therapy. During this visit:

- You will have a brief physical exam and blood drawn for routine safety blood tests, CD4+ cell count and viral load and some of your blood will be stored for future testing for this study.
- If you are a woman able to become pregnant, you will be asked to give a urine or blood sample to see if you are pregnant. You will not be able to enroll in this study if you are pregnant. You will be told the result of the test when it becomes available.
- You will be asked to provide a urine sample**.**
- You will have an anal swab and anal Pap test. A doctor or nurse will insert a swab (like a long Q-tip) into your anus. The end of the swab will be rubbed against the skin inside the anus. This sample will be tested for human papillomavirus (HPV). You will not receive the result of the HPV test as the specimen will be frozen and stored. A second swab or a soft brush will be inserted into your anus for an anal Pap test. This sample will be tested for pre-cancers of the anus. These are areas that have been damaged by HPV and might turn into cancer someday. You will receive the result of the Pap test. If you have had a recent Pap test, we can use those results so this test will not have to be repeated.
- **You will take a swab of your mouth.**

On Study Visits After Entry
You will begin taking sirolimus by mouth once a day for the next 20 weeks, in addition to the anti-HIV medicines your doctor has prescribed. You may take the sirolimus medication with or without food. Sirolimus will be supplied by the study; the anti-HIV medicines will not be supplied by the study.

During the study visits:

- Four days after you take your first dose of sirolimus, blood will be drawn to measure sirolimus levels in your blood. Blood samples will then be drawn twice a week (approximately days 4 and 7 of each week) for up to 4 weeks until two levels in a row show an adequate sirolimus level. Your dose may need to be adjusted up or down depending on the test results. Once the sirolimus level is at a steady level, blood samples will be drawn once every two weeks until 6 weeks of sirolimus treatment, after which samples will be drawn every 4 weeks.
- You will have a brief physical exam performed at five of the visits.

- You will have blood drawn for routine safety blood tests at seven of the visits and for CD4+ cell count for six of the visits and some of your blood will be stored for future testing for this study.
- You will have blood drawn to check your viral load at five of the visits.
- You will have blood drawn to test for EBV at five of the visits.
- You will have a urine sample collected at three of the visits.
- You will have another anal swab and anal Pap test at week 32.
- **You will take a swab of your mouth five other times during the study.**

Immunologic/Virologic Failure
If your CD4+ T-cell count is below 300 or it drops to less than half of what it was when you entered the study, or if your viral load is over 200, you will have another test done to check the findings. If your CD4+ T cell count is still down and/or your viral load is still up and you are still taking study drug, you will be required to stop the study drug. You will be followed on study off treatment until the final study visit at week 44. You will have the sirolimus test done one more time at your next study visit, and then stop having these tests.

Pregnancy
If you become pregnant, you will have to stop taking the study drug but we will ask you to stay in the study to be followed on study/off treatment until study completion. If you do not wish to continue to be followed on study/off treatment, the study staff will ask your permission to contact you regarding the outcome of your pregnancy.

Premature Treatment/Study Discontinuation
If you stop taking the study drug before the study-defined 20-week treatment period, you will be asked to return to the clinic to complete some evaluations.

- You will have a brief physical exam and blood drawn for routine safety blood tests, CD4+ cell count, viral load and EBV test, and some of your blood will be stored for future testing for this study.
- **You will take a swab of your mouth.**

Other
If you agree, some of your blood that is left over after all required study testing is done may be stored (with usual protectors of identity) and used for future ACTG-approved HIV-related research.

Please indicate below “yes” or “no” and initial and date whether you approve the use of these stored samples. Note that you can withdraw your consent for research on stored specimens at any time you want and the specimens will be discarded. Your refusal or withdrawal of consent for the storage of these samples will not affect your study participation since storage of leftover samples is not a requirement for the study.

______________________ Yes, I agree. ____________________ No, I do not agree.

If you agree, we would like to draw one extra tube of blood for future tests that are not known at this time. It will be stored (with usual protectors of identity). We will do this whenever we store blood for required study testing.

Please indicate below "yes" or "no" and initial and date whether you approve the use of these stored samples. Note that you can withdraw your consent for research on stored specimens at any time you want and the specimens will be discarded. Your refusal or withdrawal of consent for the extra blood draw will not affect your study participation since this the extra blood draw is not a requirement for the study.

______________________ Yes, I agree. ____________________ No, I do not agree

Future Use of Stored Samples

If you agree to let researchers store and use your leftover samples for future research, the following will happen:

- **After all routine tests required for your care are finished, instead of discarding your leftover samples we will save them in what is called a "tissue bank" for possible future research. We also will collect and save information from your medical record, including things like laboratory results.**
- **We may give your leftover samples and certain medical information about you (for example, diagnosis, blood pressure, age if less than 85) to other scientists working with the AIDS Clinical Trials Group or other companies such as data centers including to a government health research database, but we will not give them your name, address, phone number, or any other information that would identify you. Results from tests done with your leftover samples will not be given to you or your doctor. Researchers from this study, as well as other researchers from other places and institutions will have access to these leftover samples and associated health information for future research studies. However, only approved and qualified researchers will have access to your de-identified information.**
- **Sometimes leftover samples are used for genetic research (about diseases that are passed on in families). Even if we use the sample for genetic research, we will not put the results in your medical record. The research will not change the care you receive. Your sample and any information about you will be kept until it is used up or destroyed. It may be used to develop new drugs, tests, treatments or products. In some instances these may have potential commercial value. Your personal health information cannot be used for additional research without additional approval from either you or a review committee.**
- **Your leftover samples will be kept indefinitely. If you decide later that you do not want your leftover samples and information to be used for future research, you can notify the investigator in writing at [insert address], and we will destroy any remaining identifiable leftover samples and information if they are no longer needed for your care. However, if any research has already been done using portions of your leftover samples, the data will be kept and analyzed as part of those research studies.**

- **You will not receive any direct benefit for allowing the use and storage of leftover samples for future research.**

- **You will not receive any results from any of the tests done with your samples and related health information.**

- **You will not be contacted in the future for any results of studies done using your leftover samples and information.**

What risks are involved with donating samples for research?

A federal law, called the Genetic Information Nondiscrimination Act (GINA), generally makes it illegal for health insurance companies, group health plans, and most employers to discriminate against you based on your genetic information. This law generally will protect you in the following ways:

- **Health insurance companies and group health plans may not request your genetic information that we get from this research.**
- **Health insurance companies and group health plans may not use your genetic information when making decisions regarding your eligibility or premiums.**
- **Employers with 15 or more employees may not use your genetic information that we get from this research when making a decision to hire, promote, or fire you or when setting the terms of your employment.**

Be aware that this new federal law does not protect you against genetic discrimination by companies that sell life insurance, disability insurance, or long-term care insurance, nor does it protect you against genetic discrimination by all employers.

What about confidentiality?

Allowing the use and storage of leftover samples may involve a loss of privacy, but information about you will be handled as confidentially as possible. Study data will be physically and electronically secured. As with any use of electronic means to store information, there is a risk of breach of security. Your name will not be used in any published reports from research performed using your sample. The AIDS Clinical Trials Group data manager and select staff members will have access to information about you but they will not release any identifying information about you to researchers using your leftover samples.

Genetic information that results from this study does not have medical or treatment importance at this time. So, researchers have decided that there will be no sharing of results with you or your doctor. However, there is a risk that information about taking part in genetic studies using your leftover samples and information may influence insurance companies and/or employers regarding your health. To further safeguard your privacy, genetic information obtained in this study will not be placed in your medical record. Moreover, there is a chance in genetic research that even with all the security measures in place, someone using your samples, genetic information, and the databases may still find out who you are or "re-identify you." This person may also find out

information about you, your family, or people with similar genetic makeup. However, these risks today are very small but may increase with time since science and technology are developing rapidly.

Taking part in a genetic study may also have a negative impact or unintended consequences on family or other relationships. If you do not share information about taking part in this study, you will reduce this risk. Although your name will not be with the sample, it will have other facts about you such as the sequence of certain genes in your cells. These facts are important because they will help us learn how HIV persists based on these facts. Thus it is possible that study finding could one day help people of the same race, ethnicity, or sex as you. However, it is also possible through these kinds of studies that genetic traits might come to be associated with your group. In some cases, this could reinforce harmful stereotypes.

______________________ Yes, I agree. ____________________ No, I do not agree.

HOW MANY PEOPLE WILL TAKE PART IN THIS STUDY?

About 30 people will take part in this study

HOW LONG WILL I BE IN THIS STUDY?

You will be in this study for about 44 weeks (11 months).

WHY WOULD THE DOCTOR TAKE ME OFF THIS STUDY EARLY?

The study doctor may need to take you off the study early without your permission if:

- You stop your anti-HIV medicines or you need to take anti-HIV medicines that are not allowed on the study.
- The study is stopped or canceled.
- Your study doctor thinks the study is no longer in your best interest.
- Your study doctor thinks you may to be at significant risk of failing to comply with the study requirements as to cause harm to yourself or seriously interfere with the validity of the study results.
- Your primary care physician requests you be taken off the study.

The study doctor may also need to take you off the study drug without your permission if:

- Continuing the study medicine may be harmful to you.
- You need a treatment that you may not take while on the study.
- You become pregnant or are breastfeeding.
- Your CD4 count or your viral load worsens.
- You have detectable EBV in your blood.
- You are not able to take the study medicine as required by the study.
- You miss sirolimus level testing 2 times in a row.

- You miss more than 3 doses of sirolimus a week for 2 or more weeks.
- You miss 2 consecutive clinic visits.

If you must stop taking the study drug before the study is over, the study doctor will ask you to continue to be part of the study and return for some study visits and procedures.

If you must permanently stop taking sirolimus before your study participation is over, the study staff will discuss other options that may be of benefit to you. After you have completed your study participation, the study will not be able to continue to provide you with sirolimus that you received on the study. If continuing to take this or similar drugs/agents would be of benefit to you, the study staff will discuss how you may be able to obtain them.

WHAT ARE THE RISKS OF THE STUDY?

The drugs used in this study may have side effects, some of which are listed below. Please note that these lists do not include all the side effects seen with these drugs. These lists include the more serious or common side effects with a known or possible relationship. It is very important that you tell your study doctor of any changes in your medical condition while taking part in the study. At any time during the study, if you believe you are experiencing any of these side effects, you have the right to ask questions on possible and/or known risks.

There is a risk of serious and/or life threatening side effects when non-study medications are taken with the study drugs. For your safety, you must tell the study doctor or nurse about all medications you are taking before you start the study and you must ask approval for taking any new medication while you are on the study. Also, you must tell the study doctor or nurse before enrolling in any other clinical trials while on this study.

<u>Sirolimus (Rapamune)</u>

The following serious side effects may have been associated with the use of sirolimus:

- Increased risk of getting infections
- Increased risk of getting certain cancers
- Serious allergic reactions. Tell your doctor or get medical help right away if you get any of the following symptoms of an allergic reaction:
  - -Swelling of your face, eyes, or mouth, hands, feet, ankles, or lower legs
  - -Trouble breathing or wheezing
  - -Throat tightness
  - -Chest pain or tightness
  - -Feeling dizzy or faint
  - -Rash or peeling of your skin
  - -Itching
- Poor wound healing
- Increased risk for viral infection
- Lung or breathing problems. This can sometimes lead to death. **Tell your doctor if you have a new or worsening cough, difficulty breathing or any new breathing problems.**
- Unusual bleeding or bruising

- Other changes in blood test results that may show problems with the liver, kidneys, cholesterol and triglycerides (lipids or fat) levels, low red blood cell count (anemia), low platelet count (cells that help blood to clot)

Common side effects with the use of sirolimus include:

- Swelling (edema). Fluid may collect in your hands and feet and in various tissues of your body.
- Hypertension (high blood pressure)
- Abdominal pain
- Diarrhea
- Headache
- Fever
- Urinary tract infection
- Nausea
- Arthralgia (joint pain)
- **High blood sugar (diabetes)**

Potential Impact on Fertility

Sirolimus may decrease sperm counts while you are taking the drug. However, sperm counts have been shown to increase to normal levels several months after sirolimus is stopped. Sirolimus has also been associated with the absence of menstruation (monthly period) that develops in some women while they are taking the drug, and there has been at least one report of a female patient who had permanent loss of menstruation after she stopped taking sirolimus. Fertility problems are usually identified 5 to 12 months after patients start taking the drug, and you will not be asked to take sirolimus for more than 4 months. The long-term effects of sirolimus on fertility and the ability to become pregnant after taking sirolimus are not well understood and you may be at risk for temporary or long-term infertility if you enroll in this study and take study medication.

Immunologic/Virologic Failure

You may have a decrease in your CD4+ cell count and/or an increase in your viral load.

Risk of Blood Draw

Taking blood may cause some discomfort, bleeding, bruising and/or swelling where the needle enters the body and in rare cases it may result in fainting. There is a small risk of infection.

Anal Swabs

The swabs can be uncomfortable, and occasionally there can be some bleeding.

Oral Swabs

You may feel some discomfort or experience a gagging sensation when the back of your throat is touched with the swab, but the test only lasts a few seconds.

ARE THERE RISKS RELATED TO PREGNANCY?

It is not known if the drug or drug combinations in this study harm unborn babies. If you are a woman and having sex that could lead to pregnancy, you must agree not to become pregnant.

If you are a woman and participating in sexual activity that could lead to pregnancy, you and/or your male partner must use one form of birth control that you discuss with the study staff. You must start one method of birth control before you start taking your study drug and while you are taking your study drug. You must continue to use this method until 12 weeks after you stop the study drug.

- Condoms (male or female) with or without a spermicidal agent. Condoms are recommended because their appropriate use is the only contraceptive method effective for preventing HIV transmission.
- Diaphragm or cervical cap with spermicide
- Intrauterine device (IUD)
- Hormone-based contraceptive
- Tubal ligation

If you can become pregnant, you must have a pregnancy test before you enter this study and before you start taking sirolimus. The test must show that you are not pregnant. If you think you may be pregnant at any time during the study, tell your study staff right away. The study staff will talk to you about your choices. In addition, pregnancy complications and/or pregnancy outcomes that will not include any information that can identify you will be reported to the Antiretroviral Pregnancy Registry.

ARE THERE BENEFITS TO TAKING PART IN THIS STUDY?

This study is intended to gather information about how sirolimus acts on your immune system, not to treat your HIV infection. If you take part in this study, it is likely that there will be no direct benefit to you but information learned from this study may lead to the development of other studies that may help others who have HIV.

WHAT OTHER CHOICES DO I HAVE BESIDES THIS STUDY?

Instead of being in this study you have the choice of:

- Treatment with prescription drugs available to you.
- Treatment with experimental drugs, if you qualify.
- No treatment

Please talk to your doctor about these and other choices available to you. Your doctor will explain the risks and benefits of these choices.

WHAT ABOUT CONFIDENTIALITY?

We will do everything we can to protect your privacy. In addition to the efforts of the study staff to help keep your personal information private, we have gotten a Certificate of Confidentiality from the U.S. Federal Government. This certificate means that researchers cannot be forced to tell people who are not connected with this study, such as the court system, about your participation. Also, any publication of this study will not use your name or identify you personally.

People who may review your records include the ACTG, Office for Human Research Protections (OHRP) or other government agencies as part of their duties (insert name of site) IRB (a group that protects the rights and well-being of people in research), National Institutes of Health (NIH), study staff, study monitors, the drug company supporting this study and their designees. Having a Certificate of Confidentiality does not prevent you from releasing information about yourself and your participation in the study.

Even with the Certificate of Confidentiality, if the study staff learns of possible child abuse and/or neglect or a risk of harm to yourself or others, we will be required to tell the proper authorities.

A description of this clinical trial will be available on www.clinicaltrials.gov, as required by U.S. law. This website will not include information that can identify you. At most, the website will include a summary of the results. You can search this website at any time.

WHAT ARE THE COSTS TO ME?

Taking part in this study may lead to added costs to you and your insurance company. In some cases it is possible that your insurance company will not pay for these costs because you are taking part in a research study. There will be no cost to you for the sirolimus, study-related visits, physical examinations, laboratory tests, or other study procedures. Anti-HIV medicines will not be provided by the study.

WILL I RECEIVE ANY PAYMENT?

[Sites please indicate whether you will provide payment to participants]

WHAT HAPPENS IF I AM INJURED?

If you are injured as a result of being in this study, you will be given immediate treatment for your injuries. The cost for this treatment will be charged to you or your insurance company. There is no program for compensation either through this institution or the National Institutes of Health. You will not be giving up any of your legal rights by signing this consent form.

WHAT ARE MY RIGHTS AS A RESEARCH **PARTICIPANT**?

Taking part in this study is completely voluntary. You may choose not to take part in this study or leave this study at any time. Your decision will not have any impact on your participation in other studies conducted by NIH and will not result in any penalty or loss of benefits to which you are otherwise entitled.

We will tell you about new information from this or other studies that may affect your health, welfare, or willingness to stay in this study. If you want the results of the study, let the study staff know.

WHAT DO I DO IF I HAVE QUESTIONS OR PROBLEMS?

For questions about this study or a research-related injury, contact:

- name of the investigator or other study staff
- telephone number of above

For questions about your rights as a research **participant,** contact:

- name or title of person on the Institutional Review Board (IRB) or other organization appropriate for the site
- telephone number of above

SIGNATURE PAGE

If you have read this consent form (or had it explained to you), all your questions have been answered and you agree to take part in this study, please sign your name below.

______________________________	______________________________
**Participant's** Name (print)	**Participant's** Signature and Date
______________________________	______________________________
**Participant'**s Legal Representative (print)<br>(As appropriate)	Legal Representative's Signature and Date
______________________________	______________________________
Study Staff Conducting<br>Consent Discussion (print)	Study Staff's Signature and Date
______________________________	______________________________
Witness's Name (print)<br>(As appropriate)	Witness's Signature and Date

ACTG Network Coordinating Center

Social & Scientific Systems, Inc.
8757 Georgia Avenue, 12th Floor
Silver Spring, MD 20910

Phone: *(301) 628-3000*
Fax: *(301) 628-3302*

CLARIFICATION MEMO

DATE: October 4, 2016

TO: ACTG CTU Principal Investigators, CRS Leaders, and CTU/CRS Coordinators

FROM: A5337 Protocol Team

SUBJECT: Clarification Memo #1 for Protocol A5337, Version 2.0, 07/18/16, entitled, "Safety and Efficacy of Sirolimus for HIV Reservoir Reduction in Individuals on Suppressive Antiretroviral Therapy"

This clarification memo does not result in a change in the protocol informed consent document. The Division of AIDS does not require you to forward it to your institutional review board (IRB); however, as always, you must follow your IRB's policies and procedures. If IRB review of clarification memos is required at your site, please submit this document for review.

Each site should file a copy of this clarification memo with the protocol for reference.

The protocol clarifications contained in this memo should be implemented immediately. These updates will be included in the next version of the A5337 protocol if it is amended at a future date.

The following are clarifications (in bold) to protocol A5337, Version 2.0, 07/18/16:

4.2.16 Herpes-zoster or varicella-zoster viral infection requiring treatment within 90 days prior to study entry or currently on suppressive therapy **for herpes-zoster (varicella-zoster)**.

<u>NOTE</u>: Suppressive therapy for herpes simplex viruses 1 or 2 is not exclusionary.

5.1 Regimens, Administration and Duration

Participants taking a non-PI, non-NNRTI containing regimen, and those on a non-PI, RPV-based regimen, will initially receive:

- Sirolimus 0.025 mg/kg/day administered orally once daily with or without food. See Table 2.

Participants taking an NNRTI containing regimen (with the exception of RPV) will initially receive:

- Sirolimus 0.05 mg/kg/day administered orally once daily with or without food. See Table 3.

In order to achieve therapeutic levels, therapy will be initiated with the dosage noted above and dosing will be subsequently adjusted based on trough blood sirolimus concentrations to achieve target concentrations between 5 and 10 ng/mL (see Table 4). A detailed dosing table based on the adjustment algorithm in Table 4 can be found on the A5337 PSWP.

NOTE: If there is a change in ART regimen between entry and week 12, the site must contact the A5337 core team (actg.corea5337@fstrf.org) as soon as possible to reassess the initial sirolimus dose based on current ART regimen/classification at week 12.

6.2.3 Event Driven Evaluations

Confirmation visit to confirm CD4+ cell count <300 cells/mm^3 or >50% CD4+ decrease from study entry value or HIV-1 RNA >200 copies/mL (see sections 7.1.4 and 7.1.5) should be scheduled within 1 week of receipt of the abnormal lab value. If confirmed CD4+ cell count <300 cells/mm^3 or >50% CD4+ decrease from study entry value or HIV-1 RNA >200 copies/mL, the study team should be promptly contacted and sirolimus should not be started or should be stopped if already started.

NOTE: If study entry CD4+ cell count is not available, monitoring for >50% decrease should be done relative to the week 12 CD4+ cell count. If neither entry nor week 12 CD4+ cell count is available, monitoring for >50% decrease should be done relative to the screening CD4+ cell count.

ACTG Network Coordinating Center
Social & Scientific Systems, Inc.
8757 Georgia Avenue, 12th Floor
Silver Spring, Maryland 20910
Phone: (301) 628-3000
FAX: (301) 628-3002

Date: July 27, 2016

To: ACTG CTU Principal Investigators, CRS Leaders, and CTU/CRS Coordinators

From: A5337 Protocol Team

Subject: A5337, FINAL Version 2.0, dated 07/18/16

The amended A5337 protocol (Version 2.0 dated 07/18/16), "Safety and Efficacy of Sirolimus for HIV Reservoir Reduction in Individuals on Suppressive Antiretroviral Therapy," is now ready for download. A summary of changes begins on page 2 of this memo.

All sites that have received protocol registration approval for the previous protocol version (Version 1.0) must submit this protocol amendment to their local institutional review boards or ethics committees (IRBs/ECs) as soon as a possible.

The changes have affected the consent form.

Upon receiving final IRB/EC and any other applicable regulatory entity approval(s) for this amendment (Version 2.0), sites should implement the amendment immediately. Sites are required to submit an amendment registration packet to the DAIDS PRO at the RSC. For additional information on the protocol registration process and specific documents required for initial and amendment registrations, refer to the current version of the DAIDS Protocol Registration Manual and section 4.0 of the protocol document.

The Spanish Informed Consent template for Version 2.0 will be posted as soon as it is available.

Please contact the team (actg.teama5337@fstrf.org) if you have any questions.
We look forward to continuing to work with you on this study!

SUMMARY OF CHANGES
A5337 FINAL Version 2.0, 07/18/16
Safety and Efficacy of Sirolimus for HIV Reservoir Reduction in Individuals on Suppressive Antiretroviral Therapy

The main purpose of this amendment is to relax some of the eligibility criteria to increase enrollment. In addition, the amendment will incorporate all prior changes from Letter of Amendment #1 (06/29/15) and Clarification Memo #1 (09/02/15). These changes, except for deletions, are noted in bold in the protocol document.

1. The Cover Page, Contents, Sites Participating in the Study, Protocol Team Roster, Study Management, Glossary of Protocol-Specific Terms, and References sections have been updated.

2. References to an Investigational New Drug (IND) and the Food and Drug Administration (FDA) have been deleted as A5337 is exempt from IND regulations.

3. “Subject” has been changed to “participant” throughout the protocol.

4. Schema and Study Design Schematic: CD4+ cell count has been changed from ≥400 cells/mm^3 to ≥350 cells/mm^3.

5. Section 1.4.3, Exploratory Objective has been revised to also measure the effects of sirolimus on homeostatic proliferation, gene expression and transcriptional regulation.

6. Section 1.4.7, an Exploratory Objective has been added to evaluate whether sirolimus is associated with more frequent reactivation or suppression of human herpes viruses as measured in collected oral swabs.

7. Section 2.1.4, Sirolimus and cytomegalovirus (CMV) and HPV infection, has been revised to include the rationale for collecting oral swabs.

8. Section 2.1.5, Safety of sirolimus in HIV-uninfected patients has been revised to remove non-melanoma skin cancers from the FDA black box warning.

9. Section 2.2.2, Rationale for other potential mechanisms through which sirolimus may reduce HIV-1 reservoirs, has been revised to provide the rationale for exploring expression of genes in the mTOR pathway.

10. Section 3.0, Study Design
- CD4+ cell count has been changed from ≥400 cells/mm^3 to ≥350 cells/mm^3.
- Continuous ART is defined as active therapy for the 24-month period prior to study entry with no treatment interruption longer than 7 consecutive days.
- The dose adjustment table has been corrected to Table 4.

11. Section 4.1, Inclusion Criteria
- Section 4.1.2: Continuous ART has been clarified as the 24-month period prior to study entry with no treatment interruption longer than 7 consecutive days.
- Section 4.1.3: CD4+ cell count has been changed from ≥400 cells/mm^3 to ≥350 cells/mm^3.

- Section 4.1.4: Plasma HIV-1 RNA measurements have been clarified to allow for low-level viremic events (blips) during the 24-month ART period.
- Section 4.1.10: Urine protein to urine creatinine ratio has been changed from ≤1 gram to ≤1 g/g from random urine collection.

12. Section 4.2.11, Exclusion Criterion has been revised to allow participants with non-melanoma skin cancer.

13. Section 4.3.2, Protocol Activation requirements have been added.

14. Section 4.4, Coenrollment Guidelines, have been updated.

15. Section 5.1, Regimens, Administration and Duration: Sirolimus dosing tables have been updated.

16. Section 5.2, Study Product Formulation and Preparation, has been updated.

17. Section 5.4, Concomitant Medications, has been revised to correct the name and web address of the ACTG Drug Interactions Database.

18. Section 5.4.1, Required Medications, has been revised to define continuous ART as active therapy with no treatment interruption longer than 7 consecutive days.

19. Section 6.1, Schedule of Events
    - Stored serum is no longer required for inflammatory and coagulation markers, or for antibody quantitation.
    - Cryopreserved PBMCs for viral outgrowth assay was changed to inducible RNA assay.
    - Blood collection for gene expression assay was added at weeks 12, 13, 16, 24, 32, 44.
    - Stored serum is no longer required for future unspecified testing.
    - Oral swab was added at entry, weeks 12, 13, 16, 24, 32, 44, and premature discontinuation.

20. Section 6.3, Instructions for Evaluations, has been revised to require AE reporting using the DAIDS Version 2.0, November 2014 toxicity table.

21. Section 6.3.2, Medical History, has been revised to delete the requirement that diagnoses be keyed within 48 hours, and to collect additional data on duration of HIV RNA suppression, start of ART and pre-ART HIV RNA and CD4+ cell count.

22. Section 6.3.3, Medication History, has been revised to collect date of first use of ART.

23. Section 6.3.4, Clinical Assessments
    - Weight is required at all study visits.
    - Signs and symptoms that led to a change in study treatment or a change in ART must be recorded on the CRF and keyed within 48 hours.
    - All study treatment modifications in sirolimus dosing must be recorded on the CRF and keyed within 48 hours.

.

24. Section 6.3.5, Laboratory Evaluations
    - For post-entry assessments, record lipid values, and all laboratory values, regardless of grade that led to a change in study treatment or a change in ART, on the CRF and key within 48 hours.

25. Section 6.3.6, Stored Plasma and Serum for Antibody Quantitation, has been revised to delete the requirement for stored serum.

26. Section 6.3.8, Cryopreserved PBMCs for Inducible RNA Assay: Cryopreserved PBMCs for viral outgrowth assay was changed to inducible RNA assay.

27. Section 6.3.9, Gene Expression Studies, has been added.

28. Section 6.3.10, Stored Samples for Future Unspecified Assays: Stored serum is no longer required.

29. Section 6.3.11, Procedures: Oral swab collection has been added.

30. Section 7.0, Clinical Management Issues, has been revised to require AE reporting using the DAIDS Version 2.0, November 2014 toxicity table.

31. Section 7.2.4, Nephrotoxicity: Urine protein to creatinine ratios has been changed from ≥1 gram to >1 g/g.

32. Section 9.0, Statistical Considerations, has been updated.

33. Section 11.4.1, Adverse Event Reporting to DAIDS: DAERS contact information has been corrected.

34. Section 11.4.3, Grading Severity of Events, has been revised to require AE reporting using the DAIDS Version 2.0, November 2014 toxicity table.

35. APPENDIX I, SAMPLE INFORMED CONSENT

<u>Introduction</u>
The CD4+ cell count requirement for entry has been changed from 400 or greater to 350 or greater.

<u>What do I have to do if I am in this study?</u>
At the entry, week 12 post-entry, during the study, and premature treatment/study discontinuation visits, collection of an oral swab has been added.

<u>Future use of stored samples</u>: Section added that describes the procedures and safeguards for storing leftover samples that will be used for genetic testing.

<u>What are the risks of the study?</u>
- Notation added under serious side effects of sirolimus that a participant should tell his/her doctor of a new or worsening cough, difficulty breathing or any new breathing problems.

- High blood sugar (diabetes) as a common side effect of sirolimus has been added.
- The risks of oral swab collection have been added.

ACTG Network Coordinating Center

Social & Scientific Systems, Inc.
8757 Georgia Avenue, 12th Floor
Silver Spring, MD 20910

Telephone: 301-628-3000
Fax: 301-628-3302

LETTER OF AMENDMENT

DATE: January 26, 2017

TO: ACTG CTU Principal Investigators, CRS Leaders, and CTU/CRS Coordinators

FROM: A5337 Protocol Team

SUBJECT: Letter of Amendment #1 for Protocol A5337, Version 2.0, 07/18/16, entitled "Safety and Efficacy of Sirolimus for HIV Reservoir Reduction in Individuals on Suppressive Antiretroviral Therapy."

The following information impacts the A5337 study and must be forwarded to your institutional review board (IRB)/ethics committee (EC) as soon as possible for their information and review. Sites must follow the guidance of their IRB/EC.

The following information may also impact the Sample Informed Consent. Your IRB/EC will be responsible for determining the process of informing participants of the contents of this Letter of Amendment (LOA).

Sites are still required to submit an LOA registration packet to the DAIDS Protocol Registration Office (PRO) at the Regulatory Support Center. Sites will receive a registration notification for the LOA once the DAIDS PRO verifies that all the required LOA registration documents have been received and are complete. An LOA registration notification from the DAIDS PRO is not required prior to implementing the LOA. A copy of the LOA registration notification along with this letter and any IRB/EC correspondence should be retained in the site's regulatory files.

The main purpose of this amendment is to allow sirolimus study medication to be continued in study participants while awaiting the results of a prompt Safety Monitoring Committee (SMC) review of specific adverse events.

9.5 Monitoring, second paragraph

At the earlier of a) approximately 6 months after the first participant begins study treatment and b) after 12 participants have reached 12 weeks of study treatment, a Study Monitoring Committee (SMC) will be convened to review the study data and progress according to ACTG Standard Operating Procedures. The SMC will review accrual, participant characteristics, AE summaries (and listings detailing each team decision as to the relationship of the AE to study treatment), sirolimus dosages and trough levels (in particular for any participant with a Grade 3 or higher AE), CD4+ cell

counts and HIV-1 RNA levels/suppression over time, off-treatment and off-study rates (and reasons) and completeness of sample availability. In addition, if there are any Grade 4 AEs or greater than three Grade 3 AEs attributed to study treatment in two or more participants per section 9.2.1.1, ~~sirolimus use in all participants will be stopped and~~ **an SMC safety review will be conducted and a conference call held if deemed necessary by the SMC within 7 business days of receiving the safety report from the team. Sirolimus use in participants will continue while the SMC safety review is conducted. The SMC review will include an assessment of the safety of the continued use of sirolimus by study participants as well as deliberations regarding other modifications to the study to assure the safety of the participants.** With respect to monitoring AEs, any lipid laboratory value/abnormality will be considered as a single event, rather than separate events for each individual test (including fasting triglycerides, LDL, and total cholesterol). An SMC review will also be triggered if more than 25% of participants (evaluating at least 12 participants, e.g., greater than 3 of 12) discontinue study treatment due to AEs or other side effects attributable to study drug; the therapeutic duration of the trial might be reduced to 12 weeks or otherwise modified following SMC review. Subsequent SMC reviews will be approximately every 6 months. In addition, the core team or the SMC, at any time it thinks appropriate, may ask for the SMC to independently review all available safety data.
